# Global burden, trends, and inequalities of gastric cancer attributable to high-sodium diets: a 30-year analysis and projections based on the global burden of disease 2021 study

**DOI:** 10.3389/fnut.2025.1683048

**Published:** 2025-12-11

**Authors:** Qiangqiang Tian, Ya Zheng, Yishudong Li, Rui Wu, Yuyu Lin, Zhaofeng Chen

**Affiliations:** 1 Lanzhou University, Lanzhou, Gansu, China; 2 The First Hospital of Lanzhou University, Lanzhou, Gansu, China

**Keywords:** gastric cancer, high-sodium diet, global burden, trend analysis, health disparities, projection models

## Abstract

**Background:**

High-sodium diet is a modifiable risk factor for gastric cancer, contributing substantially to its global burden. However, comprehensive evaluations of temporal, geographic, and sociodemographic patterns remain limited.

**Methods:**

Using Global Burden of Disease (GBD) 2021 data, we systematically assessed gastric cancer mortality and disability-adjusted life years (DALYs) attributable to high-sodium diets across 204 countries and territories from 1990 to 2021. Analytical approaches included joinpoint regression, age-period-cohort models, frontier efficiency, inequality metrics, and Bayesian projections to 2036.

**Results:**

Globally, age-standardized mortality and DALY rates declined significantly from 1990 to 2021 (EAPC for mortality: −2.26%; DALYs: −2.88%). The highest burden was observed in East Asia, with China and Mongolia being most affected. Disparities across sex, region, and sociodemographic index (SDI) persisted, with middle and high-middle SDI countries exhibiting peak age-standardized rates. Reductions were most pronounced in high SDI and high-income Asia-Pacific regions. Inequality, while reduced in absolute terms, remained evident. Projections indicate continued declines, but persistent regional differences.

**Conclusions:**

Despite global progress in reducing age-standardized rates, substantial absolute and relative disparities in gastric cancer burden attributable to high-sodium diets persist, especially in transitioning economies. Targeted policies combining sodium reduction, education, regulation, and improved healthcare access are vital to accelerate progress and achieve more equitable health gains worldwide.

## 1 Introduction

Gastric cancer remains a major global health concern, ranking among the most common malignancies and leading causes of cancer-related mortality worldwide (1). The disease exerts a significant health and socioeconomic burden, particularly in low- and middle-income countries (2). Despite advances in prevention, diagnosis, and treatment, the global incidence and burden of gastric cancer continue to present formidable public health challenges (3). To effectively inform prevention and control efforts, identification and mitigation of modifiable risk factors are essential (4, 5).

Among established risk factors, dietary patterns—especially high sodium intake—have attracted considerable attention (6). Excessive sodium consumption, largely attributed to high salt and processed food intake, is consistently linked to gastric mucosal injury, carcinogenic processes, and increased gastric cancer risk via multiple biological mechanisms (7–9). As a result, both the World Health Organization and national health authorities have prioritized sodium reduction as a key strategy in cancer prevention (10, 11). Nevertheless, the burden of gastric cancer attributable to high-sodium diets exhibits significant geographic and temporal variability, reflecting differences in dietary behaviors, socioeconomic status, healthcare accessibility, and population demographics (12, 13).

Although prior research has emphasized overall trends in gastric cancer incidence and mortality, a lack of comprehensive and contemporary analyses persists regarding the global, regional, and national burden, temporal dynamics, and inequalities of gastric cancer specifically attributable to high-sodium diets. Such analyses are particularly scarce in the context of ongoing demographic shifts and varying stages of national development. Furthermore, underlying drivers—including demographic trends, epidemiological transitions, and inequities across sociodemographic strata—remain insufficiently elucidated. Addressing these knowledge gaps is critical for effectively targeting high-risk populations and regions, assessing the impact of current policies, and guiding evidence-based interventions.

To address these challenges, we utilized the Global Burden of Disease (GBD) 2021 study data to systematically evaluate epidemiological trends in gastric cancer attributable to high-sodium diets across 204 countries and territories from 1990 to 2021. Applying a range of analytical techniques—including joinpoint regression, age-period-cohort modeling, frontier efficiency analysis, and cross-country inequality assessment—our objectives were to (1) characterize the global, regional, and national burden and trends in gastric cancer mortality and disability-adjusted life years (DALYs) attributable to high-sodium diets; (2) quantify the impact of demographic and epidemiological changes; (3) assess inequalities across sociodemographic strata; and (4) generate projections to 2036. The findings aim to inform the refinement of sodium reduction policies and support the implementation of targeted, evidence-based gastric cancer prevention strategies worldwide.

## 2 Methods

### 2.1 Data source

This study was based on data from the Global Burden of Disease (GBD) 2021 project, which systematically quantifies the burden of 371 diseases and injuries, along with associated risk factors, across 204 countries and territories from 1990 to 2021. The GBD integrates information from multiple sources—including vital records, cancer registries, household surveys, and academic literature—standardized through advanced statistical modeling (14). DisMod-MR 2.1, a Bayesian meta-regression platform, and the MR-BRT (Meta-Regression–Bayesian, Regularized, Trimmed) tool were used to address heterogeneity among data sources. Incidence, mortality, and DALYs attributable to high-sodium diets were extracted from the GBD Results Tool (http://ghdx.healthdata.org/gbd-results-tool), and all estimates include 95% uncertainty intervals (UIs) derived from 1,000 posterior simulation draws (15).

### 2.2 Sociodemographic index (SDI)

The Sociodemographic Index (SDI) is a composite metric reflecting socioeconomic development, calculated as the geometric mean of lag-distributed income per capita, average years of schooling for those aged ≥15 years, and the total fertility rate under age 25. Countries were categorized into quintiles (low, low-middle, middle, high-middle, and high) to examine disparities in gastric cancer burden relative to development status (16).

### 2.3 Statistical analysis

#### 2.3.1 Descriptive analysis

We assessed the global, regional, and national burden of gastric cancer attributable to high-sodium diets by reporting age-standardized mortality rates (ASMR), and DALY rates (ASDR) per 100,000 population. Analyses were stratified by sex, 20 age groups, 21 GBD regions, and SDI quintile, with standardization according to the GBD world standard population. All estimates are presented with 95% UIs.

#### 2.3.2 Joinpoint regression analysis

Trends in ASIR, ASDR, and DALY rates from 1990 to 2021 were analyzed using joinpoint regression (Joinpoint Regression Program v5.0.2) to identify periods with significant trend changes (“joinpoints”). Annual percentage changes (APC) and average annual percentage changes (AAPC) were calculated, with statistical significance determined by Monte Carlo permutation tests (17).

#### 2.3.3 Age-period-cohort analysis

Age-period-cohort models based on log-linear Poisson regression and the Integrated Nested Laplace Approximation (INLA) algorithm were employed to disentangle the independent effects of age, period, and birth cohort on the disease burden. Model outputs included net drift (overall annual trend), local drift (age-specific trends), and relative risks by period and cohort (18, 19).

#### 2.3.4 Frontier analysis

Frontier analysis was used to assess country-level effectiveness in reducing age-standardized gastric cancer burden, adjusting for SDI via data envelopment analysis (DEA). An empirical efficiency frontier was constructed for each SDI level and smoothed using LOESS regression (20, 21). The vertical distance from the frontier quantified each country's “effectiveness gap.”

#### 2.3.5 Cross-country inequality analysis

Cross-national inequalities were quantified using the Slope Index of Inequality (SII) and the Concentration Index (CI). SII captures absolute inequality by regressing DALY rates against SDI rank; CI reflects relative inequality, calculated from the area between the concentration curve and line of equality (22).

#### 2.3.6 Predictive analysis

Projections of age-standardized mortality and DALY rates from 2022 to 2036 were generated using a Bayesian age-period-cohort (BAPC) model with second-order random walk smoothing (23). Analyses were stratified by sex, SDI quintile, and region, and validated using internal cross-validation against historical data (24).

### 2.4 Software and visualization

All statistical analyses were performed in R (v4.4.2) using packages including “ggplo2,” “apc,” “INLA,” “frontier,” and “ineq.” Joinpoint regression utilized the Joinpoint Regression Program (v5.0.2), while spatial visualizations were produced with “sf” and “tmap”.

## 3 Results

### 3.1 Global, regional, and national burden

**Figure 1:**
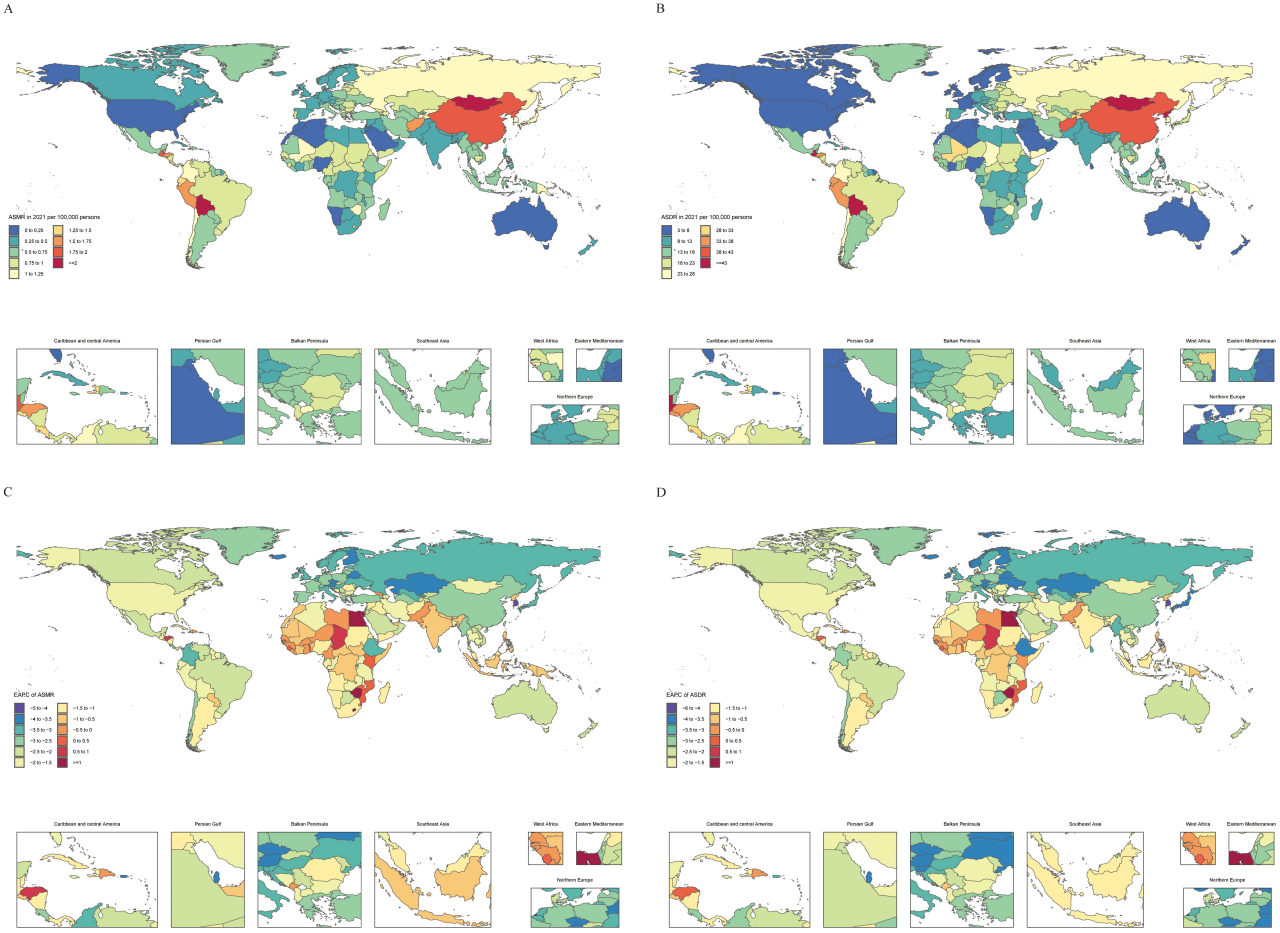
Differences in gastric cancer attributable to high-sodium diet across regions, 1990–2021. **(A)** Age-standardized mortality rate (ASMR) in 204 countries and territories in 2021; **(B)** Age-standardized disability-adjusted life-year rate (ASDR) in 204 countries and territories in 2019; **(C)** Estimated annual percentage change (EAPC) of ASMR in 204 countries and territories from 1990 to 2021; **(D)** EAPC of ASDR in 204 countries and territories from 1990 to 2021. ASR, age-standardized rate; ASMR, age-standardized mortality rate; ASDR, age-standardized DALY rate; EAPC, estimated annual percentage change; DALYs, disability-adjusted life-years; SDI, Sociodemographic Index.

Globally, the absolute number of gastric cancer deaths attributable to high-sodium diets increased substantially from 1990 to 2021, while crude DALY rates displayed a modest decline. Age-standardized mortality (ASMR) and DALY (ASDR) rates, however, fell significantly over the study period; burden consistently remained higher in males than females (see Figure 1; Supplementary Tables 1, 2). The highest absolute and age-standardized burdens were observed in East Asia, with China reporting the largest death and DALY counts, and Mongolia exhibiting the greatest age-standardized rates (see Table 1; Supplementary Table 3). Across SDI quintiles, middle SDI countries accounted for the largest number of deaths and DALYs in 2021, although the highest age-standardized rates were seen in high-middle SDI countries (Table 1).

**Table 1:** The case number and ASR of mortality and DALYs of gastric cancer attributable to high-sodium diet in 1990 and 2021 for both sexes by SDI quintiles and by GBD regions, with EAPC from 1990 to 2021.

**Location**	**Deaths**					**DALYS**				
	**Number (95 % UIs).1990**	**Number (95 % UIs).2021**	**ASR (95 % UIs).1990**	**ASR (95 % UIs).2021**	**EAPC (95 % CI) 1990–2021**	**Number (95 % UIs).1990**	**Number (95 % UIs).2021**	**ASR (95 % UIs).1990**	**ASR (95 % UIs).2021**	**EAPC (95 % CI) 1990–2021**
Global	67,845 (0 to 339,513)	75,661 (0 to 372,194)	1.74 (0 to 8.74)	0.89 (0 to 4.37)	−2.26 (−2.35 to −2.18)	1,845,617 (0 to 9,206,158)	1,804,592 (0 to 8,884,379)	44.53 (0 to 222.31)	20.78 (0 to 102.38)	−2.56 (−2.64 to −2.47)
High SDI	13,706 (0 to 69,403)	12,209 (0 to 61,722)	1.24 (0 to 6.27)	0.54 (0 to 2.73)	−2.72 (−2.75 to −2.7)	321,496 (0 to 1,625,678)	230,574 (0 to 1,154,960)	29.89 (0 to 151.09)	11.6 (0 to 58.09)	−3.11 (−3.13 to −3.08)
High-middle SDI	24,048 (0 to 122,047)	23,439 (0 to 114,553)	2.44 (0 to 12.41)	1.18 (0 to 5.79)	−2.43 (−2.56 to −2.31)	653,552 (0 to 3,320,830)	551,396 (0 to 2,681,101)	63.6 (0 to 322.96)	28.14 (0 to 136.92)	−2.76 (−2.9 to −2.63)
Middle SDI	23,250 (0 to 115,303)	28,816 (0 to 141,923)	2.3 (0 to 11.48)	1.11 (0 to 5.43)	−2.47 (−2.6 to −2.35)	667,339 (0 to 3,270,306)	712,582 (0 to 3,527,047)	59.02 (0 to 290.91)	25.82 (0 to 127.58)	−2.79 (−2.91 to −2.68)
Low-middle SDI	4,881 (0 to 24,751)	8,255 (0 to 41,816)	0.82 (0 to 4.11)	0.59 (0 to 3)	−0.95 (−1 to −0.91)	145,053 (0 to 737,564)	226,298 (0 to 1,148,265)	21.39 (0 to 108.46)	14.78 (0 to 75.09)	−1.13 (−1.16 to −1.09)
Low SDI	1,904 (0 to 9,916)	2,894 (0 to 15,443)	0.86 (0 to 4.48)	0.6 (0 to 3.18)	−1.14 (−1.19 to −1.08)	56,794 (0 to 295,505)	82,603 (0 to 441,281)	22.47 (0 to 116.97)	14.71 (0 to 78.41)	−1.41 (−1.47 to −1.36)
High-income Asia Pacific	6,059 (0 to 29,937)	5,864 (0 to 29,532)	3.09 (0 to 15.26)	1.09 (0 to 5.43)	−3.44 (−3.48 to −3.39)	151,938 (0 to 743,670)	99,017 (0 to 495,285)	74.62 (0 to 365.66)	22.57 (0 to 112.13)	−3.92 (−3.97 to −3.87)
High-income North America	1,414 (0 to 7,466)	1,482 (0 to 7,675)	0.4 (0 to 2.1)	0.23 (0 to 1.17)	−1.88 (−1.92 to −1.83)	31,635 (0 to 165,388)	32,631 (0 to 166,471)	9.34 (0 to 48.84)	5.52 (0 to 28.1)	−1.73 (−1.77 to −1.68)
Western Europe	6,132 (0 to 32,392)	4,050 (0 to 21,331)	1.04 (0 to 5.47)	0.41 (0 to 2.1)	−3 (−3.1 to −2.9)	127,536 (0 to 667,811)	76,171 (0 to 392,855)	22.85 (0 to 119.24)	8.93 (0 to 45.98)	−2.96 (−3.05 to −2.88)
Australasia	113 (0 to 630)	138 (0 to 788)	0.49 (0 to 2.7)	0.25 (0 to 1.41)	−2.1 (−2.22 to −1.99)	2,603 (0 to 14,210)	2,809 (0 to 15,483)	11.25 (0 to 61.37)	5.72 (0 to 31.14)	−2.16 (−2.26 to −2.05)
Andean Latin America	522 (0 to 2,645)	988 (0 to 4,999)	2.67 (0 to 13.48)	1.71 (0 to 8.65)	−1.64 (−1.79 to −1.5)	13,410 (0 to 68,084)	23,128 (0 to 116,684)	62.37 (0 to 316.56)	38.45 (0 to 193.86)	−1.79 (−1.93 to −1.64)
Tropical Latin America	1,356 (0 to 6,802)	1,988 (0 to 10,206)	1.58 (0 to 7.95)	0.78 (0 to 4.01)	−2.36 (−2.42 to −2.31)	35,940 (0 to 179,652)	49,223 (0 to 252,502)	37.33 (0 to 186.56)	18.88 (0 to 96.83)	−2.33 (−2.39 to −2.27)
Central Latin America	1,316 (0 to 6,625)	2,295 (0 to 11,895)	1.7 (0 to 8.53)	0.93 (0 to 4.83)	−2.18 (−2.26 to −2.1)	33,796 (0 to 170,528)	57,297 (0 to 297,890)	38.56 (0 to 194.24)	22.41 (0 to 116.52)	−2 (−2.09 to −1.92)
Southern Latin America	685 (0 to 3,455)	752 (0 to 3,767)	1.52 (0 to 7.61)	0.85 (0 to 4.26)	−1.69 (−1.8 to −1.58)	16,278 (0 to 82,265)	16,599 (0 to 82,996)	35 (0 to 176.91)	19.43 (0 to 97.04)	−1.73 (−1.84 to −1.62)
Caribbean	236 (0 to 1,223)	311 (0 to 1,671)	0.94 (0 to 4.83)	0.58 (0 to 3.1)	−1.48 (−1.55 to −1.4)	5,805 (0 to 30,338)	7,608 (0 to 40,990)	21.93 (0 to 114.63)	14.24 (0 to 76.67)	−1.31 (−1.41 to −1.21)
Central Europe	2,259 (0 to 11,339)	1,600 (0 to 7,858)	1.54 (0 to 7.69)	0.71 (0 to 3.49)	−2.58 (−2.67 to −2.5)	55,099 (0 to 277,909)	34,860 (0 to 170,909)	36.72 (0 to 185.31)	16.71 (0 to 81.88)	−2.62 (−2.71 to −2.54)
Eastern Europe	6,323 (0 to 32,996)	3,241 (0 to 16,645)	2.25 (0 to 11.72)	0.92 (0 to 4.74)	−3.11 (−3.21 to −3.01)	173,733 (0 to 903,204)	79,240 (0 to 402,533)	61.63 (0 to 320.72)	23.54 (0 to 119.38)	−3.4 (−3.52 to −3.28)
Central Asia	998 (0 to 4,972)	723 (0 to 3,725)	2.13 (0 to 10.58)	0.9 (0 to 4.6)	−2.54 (−2.64 to −2.44)	28,790 (0 to 143,224)	20,435 (0 to 105,882)	57.97 (0 to 288.43)	23.08 (0 to 119.44)	−2.8 (−2.88 to −2.73)
North Africa and Middle East	1,257 (0 to 7,341)	1,977 (0 to 11,966)	0.75 (0 to 4.4)	0.45 (0 to 2.75)	−1.58 (−1.64 to −1.51)	37,024 (0 to 214,621)	54,759 (0 to 325,676)	19.66 (0 to 114.77)	11 (0 to 65.92)	−1.83 (−1.9 to −1.77)
South Asia	3,631 (0 to 18,683)	6,499 (0 to 32,700)	0.62 (0 to 3.21)	0.45 (0 to 2.25)	−0.95 (−1.03 to −0.86)	112,571 (0 to 573,788)	180,806 (0 to 908,078)	17.02 (0 to 87.29)	11.45 (0 to 57.6)	−1.19 (−1.26 to −1.11)
Southeast Asia	2,264 (0 to 11,385)	3,572 (0 to 18,178)	0.91 (0 to 4.61)	0.56 (0 to 2.89)	−1.71 (−1.78 to −1.65)	66,676 (0 to 336,540)	97,670 (0 to 497,997)	23.49 (0 to 118.14)	13.98 (0 to 71.25)	−1.85 (−1.91 to −1.78)
East Asia	31,816 (0 to 155,224)	37,862 (0 to 188,112)	3.77 (0 to 18.42)	1.76 (0 to 8.69)	−2.54 (−2.74 to −2.34)	910,166 (0 to 4,421,005)	906,420 (0 to 4,574,158)	96.58 (0 to 469.07)	41.09 (0 to 206.63)	−2.88 (−3.06 to −2.7)
Oceania	36 (0 to 196)	72 (0 to 381)	1.36 (0 to 7.12)	1.06 (0 to 5.52)	−0.83 (−0.89 to −0.78)	1,065 (0 to 5,908)	2,144 (0 to 11,558)	32.71 (0 to 178.43)	25.52 (0 to 134.98)	−0.82 (−0.89 to −0.76)
Western Sub-Saharan Africa	479 (0 to 2,559)	880 (0 to 4,609)	0.57 (0 to 3.06)	0.49 (0 to 2.54)	−0.28 (−0.37 to −0.2)	13,169 (0 to 70,651)	24,102 (0 to 126,886)	14.02 (0 to 75.09)	11.32 (0 to 59.28)	−0.48 (−0.55 to −0.4)
Eastern Sub-Saharan Africa	658 (0 to 3,325)	852 (0 to 4,457)	0.9 (0 to 4.5)	0.54 (0 to 2.79)	−1.9 (−1.99 to −1.81)	19,716 (0 to 99,793)	24,470 (0 to 129,515)	23.37 (0 to 118.12)	12.94 (0 to 67.73)	−2.21 (−2.32 to −2.11)
Central Sub-Saharan Africa	142 (0 to 838)	262 (0 to 1,544)	0.68 (0 to 4.03)	0.51 (0 to 2.99)	−0.96 (−1 to −0.92)	4,237 (0 to 24,980)	7,864 (0 to 46,176)	16.97 (0 to 100.02)	12.54 (0 to 73.89)	−1.02 (−1.06 to −0.98)
Southern Sub-Saharan Africa	148 (0 to 796)	255 (0 to 1,387)	0.55 (0 to 3)	0.45 (0 to 2.48)	−0.69 (−1.01 to −0.37)	4,431 (0 to 23,681)	7,340 (0 to 39,115)	14.62 (0 to 78.11)	11.52 (0 to 62.18)	−0.75 (−1.08 to −0.43)

ASR, age-standardized rate; DALYs, disability-adjusted life-years; SDI, sociodemographic index; GBD, Global Burden of Diseases, Injuries, and Risk Factors Study; EAPC, estimated annual percentage change; UIs, uncertainty intervals; CI, confidence interval.

### 3.2 Temporal trends

From 1990 to 2021, the global estimated annual percentage change (EAPC) in age-standardized death and DALY rates were −2.26 (95% CI: −2.35, −2.18) and −2.88 (95% CI: −3.06, −2.70), respectively. The high-income Asia-Pacific region achieved the steepest declines, whereas Western Sub-Saharan Africa observed the smallest reductions (Table 1). At the national level, Egypt recorded the steepest rises, while the Republic of Korea had the most pronounced decreases (Supplementary Table 3). Every SDI group experienced a declining trajectory in ASMR and ASDR, with the greatest reductions noted in the high SDI group (Table 1).

### 3.3 Joinpoint analysis

**Figure 2:**
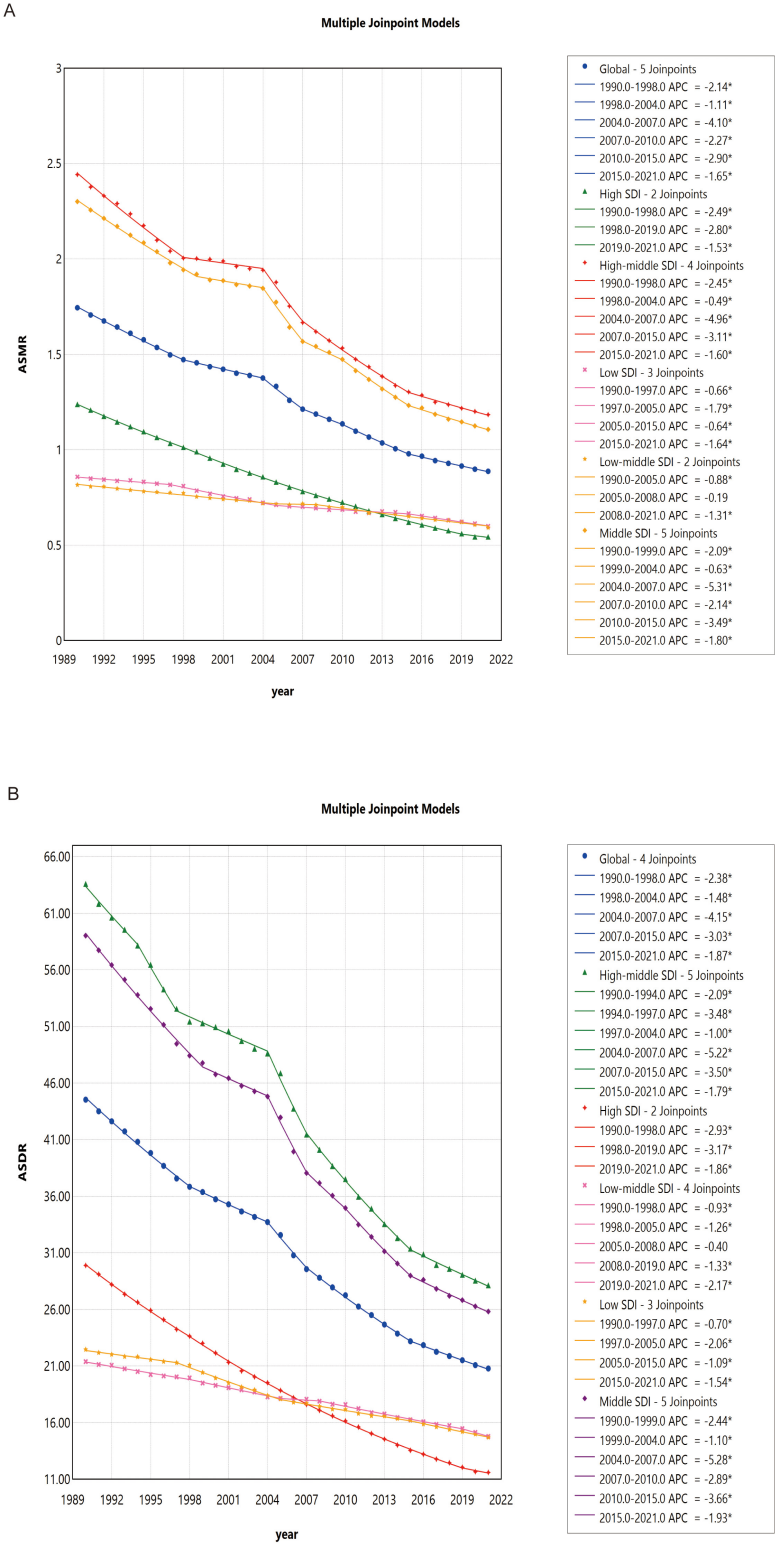
**(A)** Joinpoint regression analysis of age-standardized mortality rates (ASMR) by sex globally and in different Sociodemographic Index (SDI) regions from 1990 to 2021; **(B)** Joinpoint regression analysis of age-standardized DALY rates (ASDR) by sex globally and in different SDI regions from 1990 to 2021. ASR, age-standardized rate; ASMR, age-standardized mortality rate; ASDR, age-standardized DALY rate; DALYs, disability-adjusted life-years; SDI, Sociodemographic Index.

Joinpoint regression confirmed consistently declining mortality and DALY rates for high-sodium diet-related gastric cancer globally from 1990 to 2021, peaking in decline between 2004 and 2007. Although all SDI groups demonstrated decreases, the timing and extent varied by region. Low SDI regions saw steepest declines from 1997–2005; low-middle SDI groups improved most after 2008; middle and high-middle SDI groups had pronounced decreases during 2004–2007. The high SDI group's greatest decline occurred between 1998–2019 (see Figure 2; Supplementary Tables 4, 5). Sex-stratified trends showed similar patterns, and all 21 GBD regions exhibited falling ASMR and ASDR but with heterogeneous time dynamics (Supplementary Figures 1–3, Supplementary Tables 6–9).

### 3.4 Age-period-cohort effects

**Figure 3:**
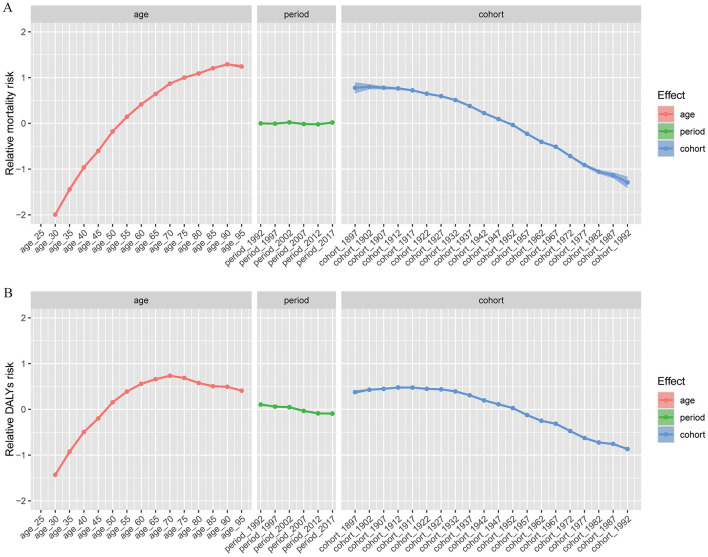
Age-period-cohort effects on the relative risk of gastric cancer attributable to high-sodium diet. **(A)** Effects on mortality; **(B)** Effects on disability-adjusted life-years (DALYs). DALYs, disability-adjusted life-years.

Age-period-cohort modeling (see Figure 3, Supplementary Figure 4) revealed significant effects for age, period, and birth cohort. Relative mortality risk increased steadily with age and peaked for DALYs in the 70–74-year group (Supplementary Figure 5). Males had higher risks than females between ages 40–85 years. Period effects for mortality remained relatively stable after adjustment, with DALY risks gradually decreasing over time. Cohort effects demonstrated higher risk among earlier birth cohorts, declining progressively in more recent cohorts. Nuanced sex differences emerged across periods and cohorts (see Supplementary Table 10).

### 3.5 Inequality

**Figure 4:**
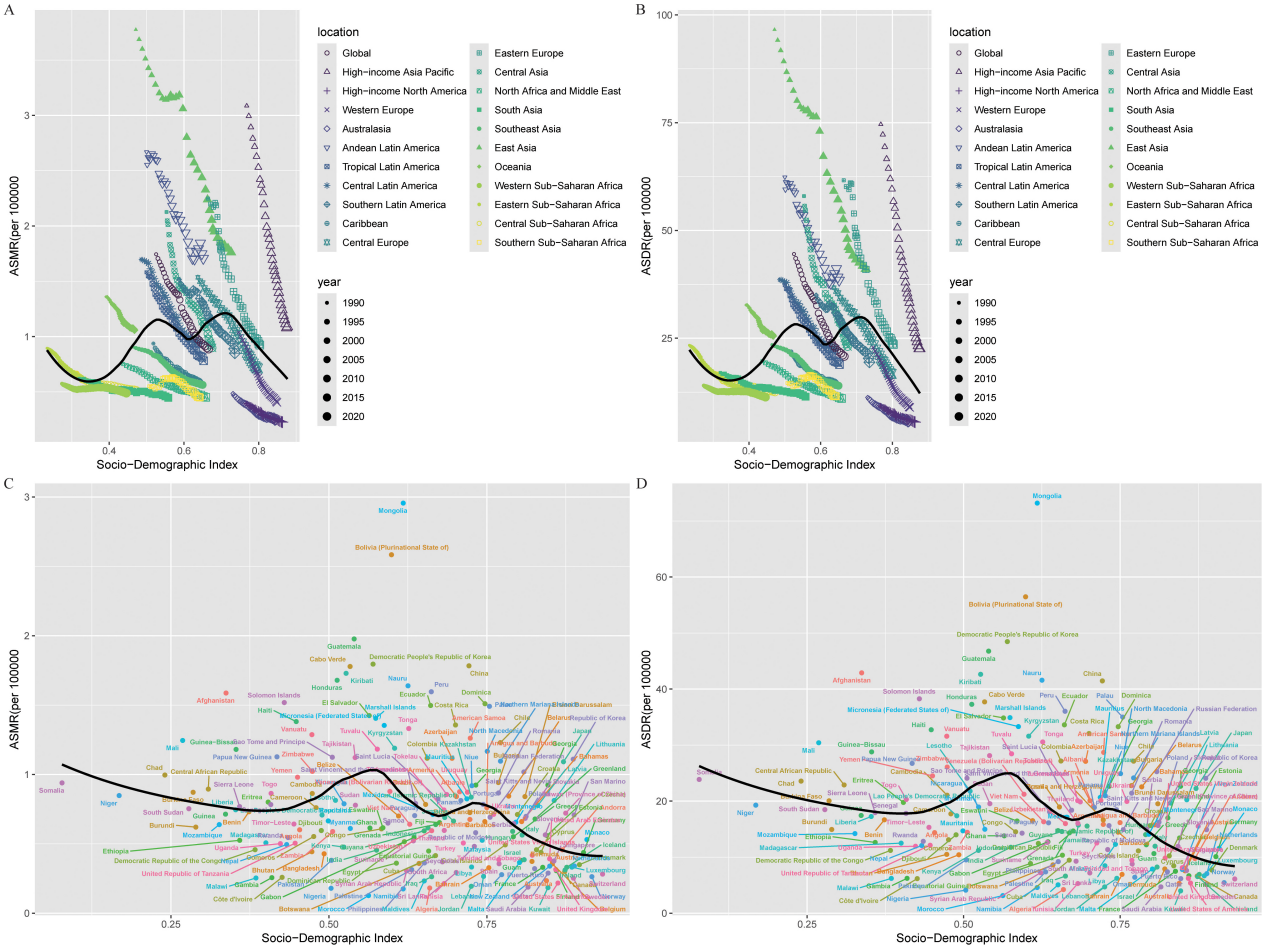
Global burden of gastric cancer attributable to high-sodium diet in relation to sociodemographic index (SDI), 1990–2021. **(A)** Relationship between SDI and ASMR by world regions in 2021; **(B)** Relationship between SDI and ASDR by world regions in 2021; **(C)** Relationship between SDI and ASMR by countries in 2021; **(D)** Relationship between SDI and ASDR by countries in 2021. ASMR, age-standardized mortality rate; ASDR, age-standardized DALY rate; DALYs, disability-adjusted life years; SDI, Sociodemographic Index.

**Figure 5:**
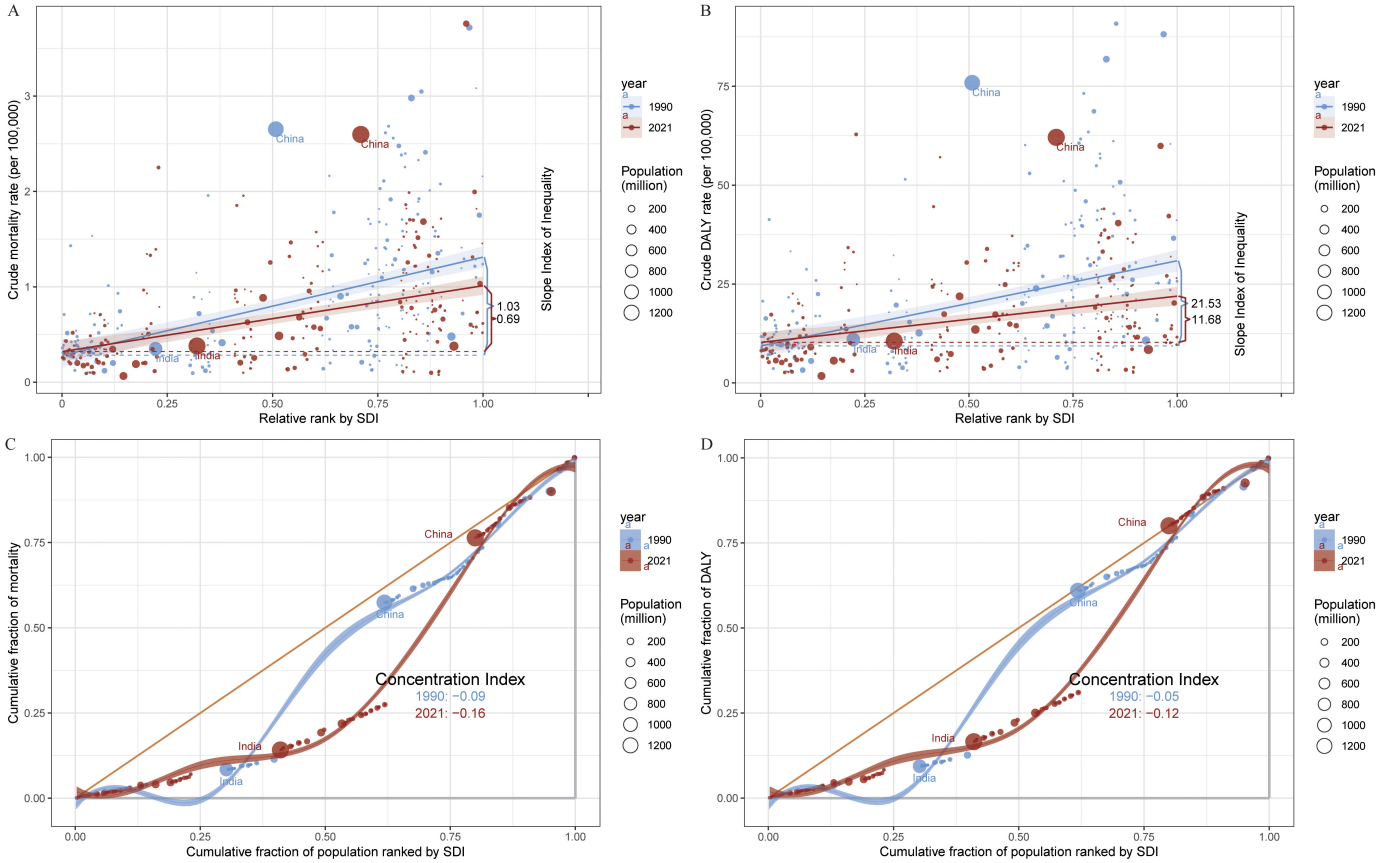
**(A)** SDI-related health inequality regression curves for mortality, 1990 and 2021; **(B)** SDI-related concentration curves for mortality, 1990 and 2021; **(C)** SDI-related health inequality regression curves for DALYs, 1990 and 2021; **(D)** SDI-related concentration curves for DALYs, 1990 and 2021. ASMR, age-standardized mortality rate; ASDR, age-standardized DALY rate; DALYs, disability-adjusted life years; SDI, Sociodemographic Index.

The association between SDI and age-standardized mortality and DALY rates was nonlinear: burden increased with SDI below 0.6, peaked between SDI 0.6–0.8, and declined at higher SDI values (see Figure 4). At the country level, both ASMR and ASDR rose with SDI up to approximately 0.6, then declined. In 2021, the Slope Index of Inequality (SII) for mortality and DALYs was 0.69 and 11.68, respectively, both lower than in 1990, reflecting reduced absolute inequality. However, the Concentration Index (−0.16 for mortality; −0.12 for DALYs) was marginally higher than in 1990, signifying persistent relative disparities across SDI strata (Figure 5).

### 3.6 Frontier analysis

**Figure 6:**
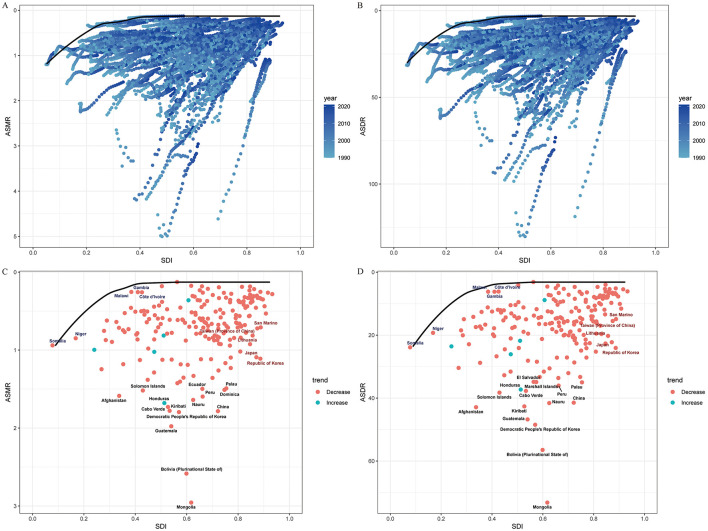
**(A)** Frontier analysis of SDI and high-sodium diet-attributable gastric cancer mortality rate from 1990 to 2021; **(B)** Frontier analysis of SDI and high-sodium diet-attributable gastric cancer mortality rate in 2021; **(C)** Frontier analysis of SDI and high-sodium diet-attributable gastric cancer DALY rate from 1990 to 2021; **(D)** Frontier analysis of SDI and high-sodium diet-attributable gastric cancer DALY rate in 2021. ASMR, age-standardized mortality rate; ASDR, age-standardized DALY rate; DALYs, disability-adjusted life years; SDI, Sociodemographic Index.

Among 204 countries and territories, frontier analysis demonstrated a general decrease in gastric cancer mortality and DALY rates regardless of SDI level, with national rates converging as SDI increased. As of 2021, 15 countries—including Mongolia, Bolivia, and North Korea—had DALY rates significantly exceeding the global efficiency frontier; nations such as Somalia and Malawi closely approached the frontier, indicating greater relative progress. For mortality, Mongolia, Bolivia, and Guatemala had the largest “effectiveness gaps.” High-SDI countries with less favorable performance included the Republic of Korea, Japan, and Lithuania (see Figure 6).

### 3.7 Projections

**Figure 7:**
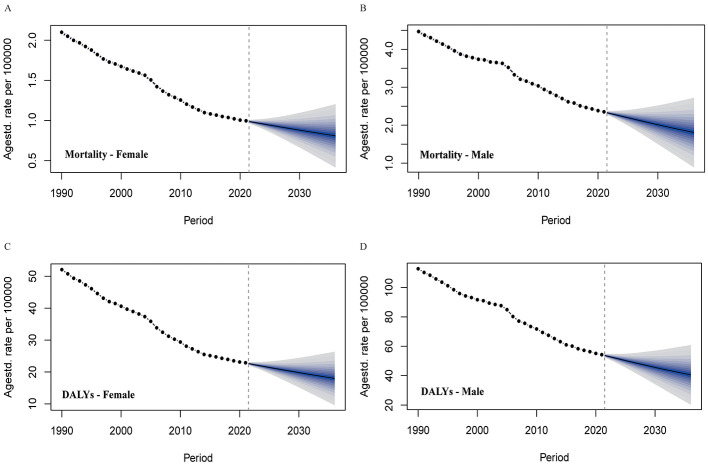
Bayesian age-period-cohort (BAPC) projections for high-sodium diet-attributable gastric cancer by sex, 2022–2036. Mortality rate projections, stratified by **(A)** females and **(B)** males; DALY projections, stratified by **(C)** females and **(D)** males. DALYs, disability-adjusted life-years.

Future projections indicate continued declines in both mortality and DALY rates between 2021 and 2036 (see Figure 7). Male mortality is expected to decrease from 2.35 to 1.81 per 100,000, and female mortality from 1.00 to 0.81 per 100,000. DALY rates are projected to fall from 54.27 to 40.55 per 100,000 in males and 22.89 to 17.99 per 100,000 in females (Supplementary Tables 11, 12). These declines remain robust across age groups, with persistent variation by sex and region over the next 15 years (Supplementary Tables 13, 14; Supplementary Figures 6, 7).

## 4 Discussion

In this comprehensive analysis using the most recent Global Burden of Disease (GBD) 2021 data, we investigated global, regional, and national burden patterns and temporal trends of gastric cancer attributable to high-sodium diet over the past three decades. Employing a diverse suite of analytical approaches, including joinpoint regression, age-period-cohort modeling, frontier analysis, and cross-country inequality assessment, we have provided a nuanced understanding of how high-sodium diet continues to shape the epidemiology of gastric cancer worldwide.

Our results demonstrate that, although the absolute number of deaths from gastric cancer attributable to high-sodium diet has increased globally, age-standardized mortality and DALY rates have declined substantially from 1990 to 2021. This decrease reflects notable progress in gastric cancer prevention, early detection, and treatment in many regions, as well as increasing public awareness and policy initiatives toward reducing sodium intake (25, 26). Contextualizing these findings within the broader epidemiological landscape of gastric cancer, the overall global burden of the disease has also declined over the same period, driven by factors such as improved sanitation, reduced *Helicobacter pylori* (*H. pylori*) infection rates, and declining tobacco use (27, 28). However, the proportion of gastric cancer attributable to high-sodium intake has remained relatively stable, accounting for approximately 10%−15% of cases based on GBD estimates, while *H. pylori* infection continues to be the dominant risk factor (attributable to ~75% of non-cardia gastric cancers) (1). Other contributors, including smoking and low fruit/vegetable intake, interact synergistically with high-sodium exposure, potentially amplifying carcinogenic effects on the gastric mucosa (29). The marked reduction in high-income Asia-Pacific and high SDI regions underscores the effectiveness of sustained public health measures, while the relatively smaller decreases observed in Western Sub-Saharan Africa and lower SDI regions highlight persistent gaps in exposure reduction, healthcare accessibility, and implementation of preventive strategies (30, 31).

Regional and national disparities remain a significant concern. East Asia continues to bear the highest absolute burden, with China contributing the largest numbers of deaths and DALYs, likely reflecting dietary patterns with traditionally higher sodium intake (32). For instance, average daily salt intake in China is estimated at 12.7 g per person—exceeding the WHO-recommended limit of 5 g—often through high consumption of salted preserved foods, sauces, and table salt (33). In Mongolia, which recorded the world's highest age-standardized mortality and DALY rates despite a smaller population, sodium intake is similarly elevated (around 11.06 g/day), compounded by diets rich in salted meats and dairy products, with limited consumption of protective foods like citrus fruits that may mitigate acid-related mucosal damage (Supplementary Table 15; Supplementary Figure 8) (34, 35). These patterns contrast with lower-intake regions, such as parts of Western Europe (6–8 g/day), where greater emphasis on fresh produce and lemon-containing foods may contribute to reduced risk (36). Notably, the efficiency frontier analysis revealed that many high-mortality or high-DALY countries consistently underperform relative to their development status, identifying critical gaps for targeted interventions (37). In high-burden countries like China, efforts to reduce high-sodium diets have included national campaigns such as the “China Healthy Lifestyle for All” initiative, which promotes salt reduction through public education, food labeling regulations, and reformulation of processed foods, alongside collaborations with the food industry to lower sodium in staples like soy sauce (38).

The joinpoint regression and age-period-cohort analyses elucidated the underlying drivers of temporal trends in gastric cancer burden (39). Our findings show that the most significant declines in mortality and DALY rates occurred between 2004 and 2007 in several SDI groups, possibly as a result of policy implementation and improved healthcare infrastructure during those years, including the rollout of WHO-led sodium reduction guidelines and enhanced cancer screening programs in transitioning economies (40). This period coincided with global health initiatives, such as the 2003 WHO Framework Convention on Tobacco Control (which indirectly supported broader NCD prevention) and early national salt reduction strategies in high-burden regions, potentially accelerating declines; however, post-2007 trends showed a slight slowdown or plateau, which may reflect challenges in sustaining momentum amid economic pressures and dietary shifts toward processed foods (41). Regarding the joinpoint regression, it is important to note that GBD results are derived from complex Bayesian modeling, which could introduce smoothing effects that influence the detection of inflection points; nonetheless, this analysis offers strengths over simple graphical trends (as in Figure 2) by providing statistically rigorous quantification of annual percentage changes (APCs) and identifying precise joinpoints through Monte Carlo permutation tests, enabling more robust inference on trend shifts compared to visual inspection alone (42). Age and cohort effects were pronounced, with older age and earlier birth cohorts associated with higher risks, reflecting cumulative long-term dietary exposures and historical lack of preventive measures (43). Specifically, the strong cohort effect may be explained by generational differences in lifetime sodium exposure: earlier cohorts (e.g., born before 1950) likely experienced higher cumulative intake due to traditional high-salt preservation methods and limited awareness of risks, whereas later cohorts benefited from globalization-driven dietary diversification, improved food regulations, and public health campaigns that reduced exposure over time (44). The period effects suggested only modest recent improvements, implying the need for continued reinforcement of sodium reduction policies (45). Furthermore, our sex-stratified analyses indicate that males consistently experience higher burden, aligning with established biological, behavioral, and exposure differences between sexes.

Despite global progress, notable inequalities persist across sociodemographic lines. The nonlinear association between SDI and age-standardized rates—where burden peaks in middle SDI countries—suggests that nations in socio-economic transition face particular challenges (46). While absolute inequality in burden has declined, relative disparities remain marked, indicating that progress is not evenly distributed (47). Countries with rapid economic growth may experience increasing sodium exposure through dietary westernization and processed food consumption before the implementation of robust public health countermeasures (48).

Projections to 2036 are encouraging, indicating continued declines in age-standardized mortality and DALY rates among both sexes and across all age groups and regions. Nevertheless, the projected differences by age, sex, and country highlight the importance of implementing tailored sodium reduction and cancer prevention strategies that address unique local risk profiles, demographic characteristics, and health system capacities. While our analysis focuses on gastric cancer, it is worth noting that high-sodium intake contributes to a broader spectrum of health impacts, including hypertension, cardiovascular diseases, and stroke, which collectively amplify the urgency for sodium reduction policies (49). Integrating these multifaceted benefits into policy frameworks could strengthen advocacy and resource allocation for comprehensive interventions.

This study has several key strengths, including the use of a robust, comprehensive, and standardized global dataset, and the application of multi-faceted analytic approaches that enable insights into both overall patterns and underlying causes of change. However, several limitations warrant consideration. The GBD estimates rely on the quality and completeness of underlying data sources, which vary substantially across countries and regions—particularly in areas with limited cancer registration or vital statistics infrastructure. Potential misclassification or under-reporting could bias burden estimates, although the Bayesian meta-regression methods used help mitigate some of these issues. The attribution of gastric cancer burden to high-sodium diet is based on modeled risk factor-exposure and disease-outcome relationships, which may be subject to residual confounding and ecological fallacy. Lastly, projections inherently depend on the assumption that past trends and relationships will continue, which may not fully capture future policy changes, economic development, or health system improvements.

In conclusion, our study underscores that, while substantial progress has been achieved in reducing the global burden of gastric cancer attributable to high-sodium diet, large disparities remain—particularly in countries at intermediate stages of socio-economic development and in certain high-burden settings. Continued and enhanced efforts to reduce sodium intake—especially in middle and low SDI nations—are urgently needed. Policy action should be multi-pronged, combining food reformulation, public health education, regulatory action, and improved healthcare access. Our findings provide important evidence to guide national and global strategies for gastric cancer prevention and can promote a more equitable reduction of disease burden in the decades to come.

## Data Availability

The original contributions presented in the study are included in the article/Supplementary material, further inquiries can be directed to the corresponding author.

## Supplementary material

Supplementary Figure 1 Joinpoint regression analysis of age-standardized rates (ASR) for mortality and disability-adjusted life-years (DALYs) due to high-sodium diet-attributable gastric cancer, globally and across Sociodemographic Index (SDI) quintiles, from 1990 to 2021: **(A)** Mortality ASR in females; **(B)** Mortality ASR in males; **(C)** DALY ASR in females; **(D)** DALY ASR in males. ASR, age-standardized rate; DALYs, disability-adjusted life-years; SDI, Sociodemographic Index.

Supplementary Figure 2 Joinpoint regression analysis of age-standardized mortality rates (ASR) for high-sodium diet-attributable gastric cancer by sex and world regions, 1990–2021: **(A)** Africa and the Middle East; **(B)** Americas; **(C)** Asia-Pacific; **(D)** Europe. Abbreviation: ASR, age-standardized rate.

Supplementary Figure 3 Joinpoint regression analysis of age-standardized disability-adjusted life-year rates (ASR of DALYs) for high-sodium diet-attributable gastric cancer by sex and world regions, 1990–2021: **(A)** Africa and the Middle East; **(B)** Americas; **(C)** Asia-Pacific; **(D)** Europe. Abbreviations: ASR, age-standardized rate; DALYs, disability-adjusted life-years.

Supplementary Figure 4 Interaction effects between age, period, and cohort on high-sodium diet-attributable gastric cancer burden: **(A, C, E)** Interaction effects on mortality: age-period **(A)**, age-cohort **(C)**, and cohort-period **(E)**; **(B, D, F)** Interaction effects on DALYs: age-period **(B)**, age-cohort **(D)**, and cohort-period **(F)**.

Supplementary Figure 5 Age-period-cohort effects on the relative risks of high-sodium diet-attributable gastric cancer, stratified by sex: **(A)** Mortality in females; **(B)** Mortality in males; **(C)** DALYs in females; **(D)** DALYs in males.

Supplementary Figure 6 Bayesian age-period-cohort (BAPC) model projections of high-sodium diet-attributable gastric cancer mortality in 21 Global Burden of Disease (GBD) regions by sex, 2022–2036.

Supplementary Figure 7 Bayesian age-period-cohort (BAPC) model projections of high-sodium diet-attributable gastric cancer DALYs in 21 Global Burden of Disease (GBD) regions by sex, 2022–2036.

Supplementary Figure 8 **(A)** Pie chart illustrating the distribution of Age-Standardized Mortality Rates (ASMR) for China, Mongolia, and the mean of all other countries. **(B)** Pie chart illustrating the distribution of Age-Standardized DALYs Rates (ASDR) for China, Mongolia, and the mean of all other countries.

Supplementary Table 1 The case number and ASR of mortality and DALYs of gastric cancer attributable to high-sodium diet in 1990 and 2021 for female by SDI quintiles and by GBD regions, with EAPC from 1990 to 2021. ASR, age-standardized rate; DALYs, disability-adjusted life-years; SDI, sociodemographic index; GBD, Global Burden of Diseases, Injuries, and Risk Factors Study; EAPC, estimated annual percentage change; UIs, uncertainty intervals; CI, con?dence interval.

Supplementary Table 2 The case number and ASR of mortality and DALYs of Gastric cancer due to high sodium diet in 1990 and 2021 for Male by SDI quintiles and by GBD regions, with EAPC from 1990 to 2021. ASR, age-standardized rate; DALYs, disability-adjusted life-years; SDI, sociodemographic index; GBD, Global Burden of Diseases, Injuries, and Risk Factors Study; EAPC, estimated annual percentage change; UIs, uncertainty intervals; CI, con?dence interval.

Supplementary Table 3 The case number and ASR of mortality and DALYs of gastric cancer attributable to high-sodium diet in 1990 and 2021 for both sexes by SDI quintiles, by GBD regions and 204 countries and regions, with EAPC from 1990 to 2021. ASR, age-standardized rate; DALYs, disability-adjusted life-years; SDI, sociodemographic index; GBD, Global Burden of Diseases, Injuries, and Risk Factors Study; EAPC, estimated annual percentage change; UIs, uncertainty intervals; CI, con?dence interval.

Supplementary Table 4 The APC and AAPC in ASR of Gastric cancer due to high sodium diet mortality for both sexes from 1990 to 2021.

Supplementary Table 5 The APC and AAPC in ASR of Gastric cancer due to high sodium diet DALYs for both sexes from 1990 to 2021.

Supplementary Table 6 The APC and AAPC in ASR of Gastric cancer due to high sodium diet mortality for female from 1990 to 2021.

Supplementary Table 7 The APC and AAPC in ASR of Gastric cancer due to high sodium diet mortality for male from 1990 to 2021.

Supplementary Table 8 The APC and AAPC in ASR of Gastric cancer due to high sodium diet DALYs for female from 1990 to 2021.

Supplementary Table 9 The APC and AAPC in ASR of Gastric cancer due to high sodium diet DALYs for male from 1990 to 2021.

Supplementary Table 10 RRs of Gastric cancer (due to high sodium diet) mortality and DALYs for female and male due to age, period, and birth cohort effects. RRs, relative risk; CI, confidence interval.

Supplementary Table 11 The predicted ASR of mortality of Gastric cancer attributable to high-sodium diet from 2022 to 2036. ASR, age-standardized rate.

Supplementary Table 12 The predicted ASR of DALYs of Gastric cancer attributable to high-sodium diet from 2022 to 2036. ASR, age-standardized rate; DALYs, disability-adjusted life-years.

Supplementary Table 13 The predicted ASR of mortality of Gastric cancer attributable to high-sodium diet from 2022 to 2036 globally by age.

Supplementary Table 14 The predicted ASR of DALYs of Gastric cancer attributable to high-sodium diet from 2022 to 2036 globally by age.

Supplementary Table 15 Average daily salt intake comparison (g/day per adult, approximate values based on recent estimates).

## References

[1] GBD 2017 Stomach Cancer Collaborators. The global, regional, and national burden of stomach cancer in 195 countries, 1990-2017: a systematic analysis for the Global Burden of Disease study 2017. Lancet Gastroenterol Hepatol. (2020) 5:42–54. doi: 10.1016/S2468-1253(19)30328-0 31648970 PMC7033564

[2] Qin N Fan Y Yang T Yang Z Fan D. The burden of Gastric Cancer and possible risk factors from 1990 to 2021, and projections until 2035: findings from the Global Burden of Disease Study 2021. Biomark Res. (2025) 13:5. doi: 10.1186/s40364-024-00720-8 39773334 PMC11708091

[3] Ning FL Lyu J Pei JP Gu WJ Zhang NN Cao SY . The burden and trend of gastric cancer and possible risk factors in five Asian countries from 1990 to 2019. Sci Rep. (2022) 12:5980. doi: 10.1038/s41598-022-10014-4 35395871 PMC8993926

[4] Yang X Zhang T Zhang H Sang S Chen H Zuo X. Temporal trend of gastric cancer burden along with its risk factors in China from 1990 to 2019, and projections until 2030: comparison with Japan, South Korea, and Mongolia. Biomark Res. (2021) 9:84. doi: 10.1186/s40364-021-00340-6 34784961 PMC8597246

[5] Jiang L Wang A Yang S Fang H Wang Q Li H . The burden of gastric cancer attributable to high sodium intake: a longitudinal study from 1990 to 2019 in China. Nutrients. (2023) 15:5088. doi: 10.3390/nu15245088 38140347 PMC10745903

[6] Zavros Y Merchant JL. The immune microenvironment in gastric adenocarcinoma. Nat Rev Gastroenterol Hepatol. (2022) 19:451–67. doi: 10.1038/s41575-022-00591-0 35288702 PMC9809534

[7] Wu X Chen L Cheng J Qian J Fang Z Wu J. Effect of dietary salt intake on risk of gastric cancer: a systematic review and meta-analysis of case-control studies. Nutrients. (2022) 14:4260. doi: 10.3390/nu14204260 36296944 PMC9609108

[8] Smyth EC Nilsson M Grabsch HI van Grieken NC Lordick F. Gastric cancer. Lancet. (2020) 396:635–48. doi: 10.1016/S0140-6736(20)31288-5 32861308

[9] Poorolajal J Moradi L Mohammadi Y Cheraghi Z Gohari-Ensaf F. Risk factors for stomach cancer: a systematic review and meta-analysis. Epidemiol Health. (2020) 42:e2020004. doi: 10.4178/epih.e2020004 32023777 PMC7056944

[10] Maddineni G Xie JJ Brahmbhatt B Mutha P. Diet and carcinogenesis of gastric cancer. Curr Opin Gastroenterol. (2022) 38:588–91. doi: 10.1097/MOG.0000000000000875 36165035

[11] Kwak JH Eun CS Han DS Kim Y-S Song K-S Choi B-Y . Gastric cancer and the daily intake of the major dish groups contributing to sodium intake: a case-control study in Korea. Nutrients. (2021) 13:1365. doi: 10.3390/nu13041365 33921757 PMC8072798

[12] Kwak JH Eun CS Han DS Kim HJ. Effects of RAD50 SNP, sodium intake, and H. pylori infection on gastric cancer survival in Korea Gastric Cancer. (2024) 27:210–20. doi: 10.1007/s10120-023-01441-x 38070008

[13] D'Elia L Galletti F Strazzullo P. Dietary salt intake and risk of gastric cancer. Cancer Treat Res. (2014) 159:83–95. doi: 10.1007/978-3-642-38007-5_6 24114476

[14] GBD 2019 Diseases and Injuries Collaborators. Global burden of 369 diseases and injuries in 204 countries and territories, 1990-2019: a systematic analysis for the Global Burden of Disease Study 2019. Lancet. (2020) 396:204–22. doi: 10.1016/s0140-6736(20)30925-9 PMC7567026 33069326

[15] GBD GBD 2019 Risk Factors Collaborators Global burden of 87 risk factors in 204 countries and territories 1990-2019: 1990-2019: a systematic analysis for the Global Burden of Disease Study 2019. Lancet. (2020) 396:1223–49. doi: 10.1016/s0140-6736(20)30752-2 33069327 PMC7566194

[16] GBD 2021 Diseases Injuries Collaborators Global incidence prevalence years lived with disability (YLDs) disability-adjusted life-years (DALYs) healthy life expectancy (HALE) for 371 diseases . Lancet. (2024) 403:2133–61. doi: 10.1016/s0140-6736(24)00757-8 38642570 PMC11122111

[17] Kim HJ Fay MP Feuer EJ Midthune DN. Permutation tests for joinpoint regression with applications to cancer rates. Stat Med. (2000) 19:335–51. doi: 10.1002/(SICI)1097-0258(20000215)19:3&lt;335::AID-SIM336&gt;3.3.CO;2-Q 10649300

[18] Rosenberg PS Miranda-Filho A Whiteman DC. Comparative age-period-cohort analysis. BMC Med Res Methodol. (2023) 23:238. doi: 10.1186/s12874-023-02039-8 37853346 PMC10585891

[19] Heo J Jeon SY Oh CM Hwang J Oh J Cho Y. The unrealized potential: cohort effects and age-period-cohort analysis. Epidemiol Health. (2017) 39:e2017056. doi: 10.4178/epih.e2017056 29309721 PMC5790985

[20] Nepomuceno TCC Piubello Orsini L de Carvalho VDH Poleto T Leardini C. The core of healthcare efficiency: a comprehensive bibliometric review on frontier analysis of hospitals. Healthcare. (2022) 10:1316. doi: 10.3390/healthcare10071316 35885842 PMC9318001

[21] Chen YJ Shimizu Bassi G Wang Y Yang YQ. Research hotspot and frontier analysis of traditional Chinese medicine in asthma using bibliometric methods from 1991 to 2021. J Allergy Clin Immunol Glob. (2022) 1:185–97. doi: 10.1016/j.jacig.2022.07.004 37779535 PMC10509992

[22] Hosseinpoor AR Bergen N Schlotheuber A. Promoting health equity: WHO health inequality monitoring at global and national levels. Glob Health Action. (2015) 8:29034. doi: 10.3402/gha.v8.29034 26387506 PMC4576419

[23] Jürgens V Ess S Cerny T Vounatsou P. A Bayesian generalized age-period-cohort power model for cancer projections. Stat Med. (2014) 33:4627–36. doi: 10.1002/sim.6248 24996118

[24] Knoll M Furkel J Debus J Abdollahi A Karch A Stock C. An R package for an integrated evaluation of statistical approaches to cancer incidence projection. BMC Med Res Methodol. (2020) 20:257. doi: 10.1186/s12874-020-01133-5 33059585 PMC7559591

[25] Takasu A Gotoda T Suzuki S Kusano C Goto C Ishikawa H . Daily diet and nutrition risk factors for gastric cancer incidence in a Japanese population. Gut Liver. (2024) 18:602–10. doi: 10.5009/gnl230354 38388181 PMC11249943

[26] Strazzullo P Abate V. Sodium. Adv Nutr. (2025) 16:100409. doi: 10.1016/j.advnut.2025.100409 40086509 PMC12002814

[27] Mamun TI Younus S Rahman MH. Gastric cancer-Epidemiology, modifiable and non-modifiable risk factors, challenges and opportunities: an updated review. Cancer Treat Res Commun. (2024) 41:100845. doi: 10.1016/j.ctarc.2024.100845 39357127

[28] Yang WJ Zhao HP Yu Y Wang J-H Guo L Liu J-Y . Updates on global epidemiology, risk and prognostic factors of gastric cancer. World J Gastroenterol. (2023) 29:2452–68. doi: 10.3748/wjg.v29.i16.2452 37179585 PMC10167900

[29] Karimi P Islami F Anandasabapathy S Freedman ND Kamangar F. Gastric cancer: descriptive epidemiology, risk factors, screening, and prevention. Cancer Epidemiol Biomarkers Prev. (2014) 23:700–13. doi: 10.1158/1055-9965.EPI-13-1057 24618998 PMC4019373

[30] Kronsteiner-Gicevic S Thompson AS Gaggl M Bell W Cassidy A Kühn T. Adding salt to food at table as an indicator of gastric cancer risk among adults: a prospective study. Gastric Cancer. (2024) 27:714–21. doi: 10.1007/s10120-024-01502-9 38630317 PMC11193689

[31] He P Li X Zou D Tang F Chen H Li Y. Environmental factors inducing gastric cancer: insights into risk and prevention strategies. Discov Oncol. (2025) 16:25. doi: 10.1007/s12672-025-01771-5 39786603 PMC11717776

[32] Wang Y Li Y Lu Z Li Z Wang R Wang Z . The global magnitude and temporal trend of hypertensive heart disease burden attributable to high sodium intake from 1990 to 2021. Curr Probl Cardiol. (2025) 50:102931. doi: 10.1016/j.cpcardiol.2024.102931 39566868

[33] Fang K He Y Fang Y Lian Y. Dietary sodium intake and food sources among Chinese adults: data from the CNNHS 2010-2012. Nutrients. (2020) 12:453. doi: 10.3390/nu12020453 32054013 PMC7071264

[34] Yamada C Oyunchimeg D Erdenbat A . Estimation of salt intake and recommendation for iodine content in iodized salt in Mongolia. Asia Pac J Public Health. (2000) 12:27–31. doi: 10.1177/101053950001200106 11200214

[35] Enkhtungalag B Batjargal J Chimedsuren O Tsogzolmaa B Anderson CS Webster J. Developing a national salt reduction strategy for Mongolia. Cardiovasc Diagn Ther. (2015) 5:229–37. doi: 10.3978/j.issn.2223-3652.2015.04.11 26090334 PMC4451311

[36] EFSA EFSA Panel on Nutrition Novel Foods and Food Allergens (NDA) Turck D Castenmiller J de Henauw S Hirsch-Ernst K-I Kearney J . Dietary reference values for sodium. EFSA J. (2019) 17:e05778. doi: 10.2903/j.efsa.2019.5778 32626425 PMC7009309

[37] Contreras Navarro A Gallagher K Griffin S Leydon CL Perry IJ Harrington JM. Systematic review on the impact of salt-reduction initiatives by socioeconomic position to address health inequalities in adult populations. Nutr Rev. (2025) 83:e1090–e100. doi: 10.1093/nutrit/nuae088 38976594 PMC11819476

[38] Zhang P He FJ Li Y Li C Wu J Ma J . Reducing salt intake in China with “Action on Salt China” (ASC): protocol for campaigns and randomized controlled trials. JMIR Res Protoc. (2020) 9:e15933. doi: 10.2196/15933 32271155 PMC7180507

[39] Maruthappu M Painter A Watkins J Williams C Ali R Zeltner T . Unemployment, public-sector healthcare spending and stomach cancer mortality in the European Union, 1981-2009. Eur J Gastroenterol Hepatol. (2014) 26:1222–7. doi: 10.1097/MEG.0000000000000201 25210778

[40] Jo HH Kim N Jang J Choi Y Lee JW. Differences in the effect of physical activity on the prevention of gastric cancer according to sex. J Gastric Cancer. (2025) 25:343–55. doi: 10.5230/jgc.2025.25.e17 40200877 PMC11982501

[41] Hu N McLean R. Targeted approaches: choosing sodium reduction methods based on salt usage habits. Nutrients. (2024) 16:2816. doi: 10.3390/nu16172816 39275134 PMC11397227

[42] Liu X Yu C Bi Y Zhang ZJ. Trends and age-period-cohort effect on incidence and mortality of prostate cancer from 1990 to 2017 in China. Public Health. (2019) 172:70–80. doi: 10.1016/j.puhe.2019.04.016 31220754

[43] He KJ Gong G. Global trends and projections of colorectal, esophageal and stomach cancer burden among youth associated with diet: a analysis of 204 countries and territories from 1990 to 2019 and until 2040. Transl Oncol. (2024) 46:101984. doi: 10.1016/j.tranon.2024.101984 38824874 PMC11170277

[44] Kim J Park S Nam BH. Gastric cancer and salt preference: a population-based cohort study in Korea. Am J Clin Nutr. (2010) 91:1289–93. doi: 10.3945/ajcn.2009.28732 20219954

[45] GBD 2021 Japan Collaborators. Three decades of population health changes in Japan, 1990-2021: a subnational analysis for the Global Burden of Disease Study 2021. Lancet Public Health. (2025) 10:e321–e32. doi: 10.1016/S2468-2667(25)00044-1 40122087 PMC11959113

[46] Tao J Quan J El Helali A Lam WWT Pang H. Global trends indicate increasing consumption of dietary sodium and fiber in middle-income countries: a study of 30-year global macrotrends. Nutr Res. (2023) 118:63–9. doi: 10.1016/j.nutres.2023.07.005 37598558

[47] Inoue M Tsugane S. Epidemiology of gastric cancer in Japan. Postgrad Med J. (2005) 81:419–24. doi: 10.1136/pgmj.2004.029330 15998815 PMC1743301

[48] Godos J Giampieri F Al-Qahtani WH Scazzina F Bonaccio M Grosso G. Ultra-Processed food consumption and relation with diet quality and mediterranean diet in Southern Italy. Int J Environ Res Public Health. (2022) 19:11360. doi: 10.3390/ijerph191811360 36141629 PMC9517140

[49] He FJ Tan M Ma Y MacGregor GA. Salt reduction to prevent hypertension and cardiovascular disease: JACC state-of-the-art review. J Am Coll Cardiol. (2020) 75:632–47. doi: 10.1016/j.jacc.2019.11.055 32057379

