# Supplementary figures and images for: Global burden, trends, and inequalities of gastric cancer attributable to high-sodium diets: a 30-year analysis and projections based on the global burden of disease 2021 study
Source: Front Nutr. 2025 Dec 11;12:1683048. doi: 10.3389/fnut.2025.1683048 (PMC12739881; doi:10.3389/fnut.2025.1683048)

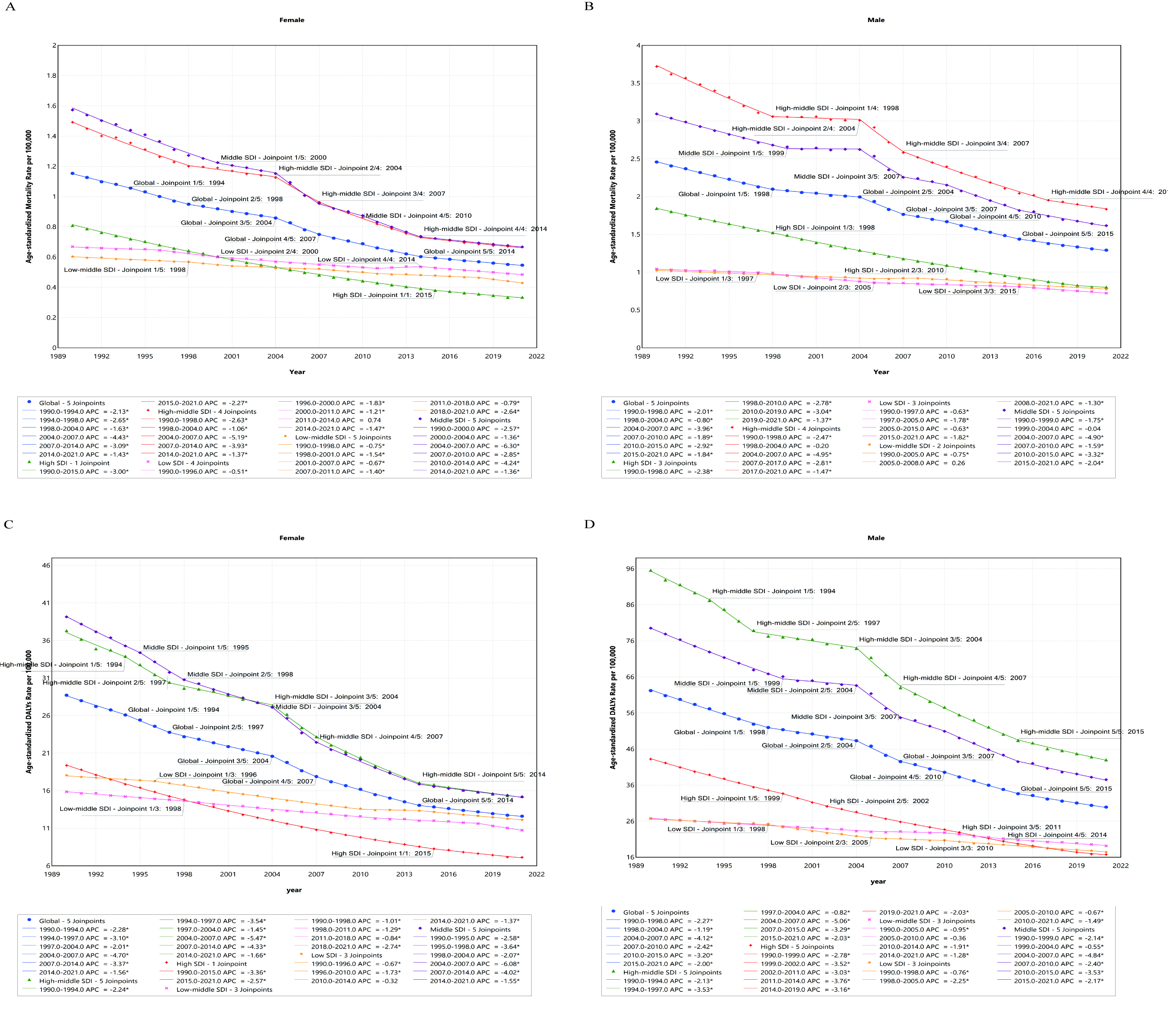

Supplement: Supplementary Figure 1 — Joinpoint regression analysis of age-standardized rates (ASR) for mortality and disability-adjusted life-years (DALYs) due to high-sodium diet-attributable gastric cancer, globally and across Sociodemographic Index (SDI) quintiles, from 1990 to 2021: (A) Mortality ASR in females; (B) Mortality ASR in males; (C) DALY ASR in females; (D) DALY ASR in males. ASR, age-standardized rate; DALYs, disability-adjusted life-years; SDI, Sociodemographic Index. [file Image_1.jpeg]

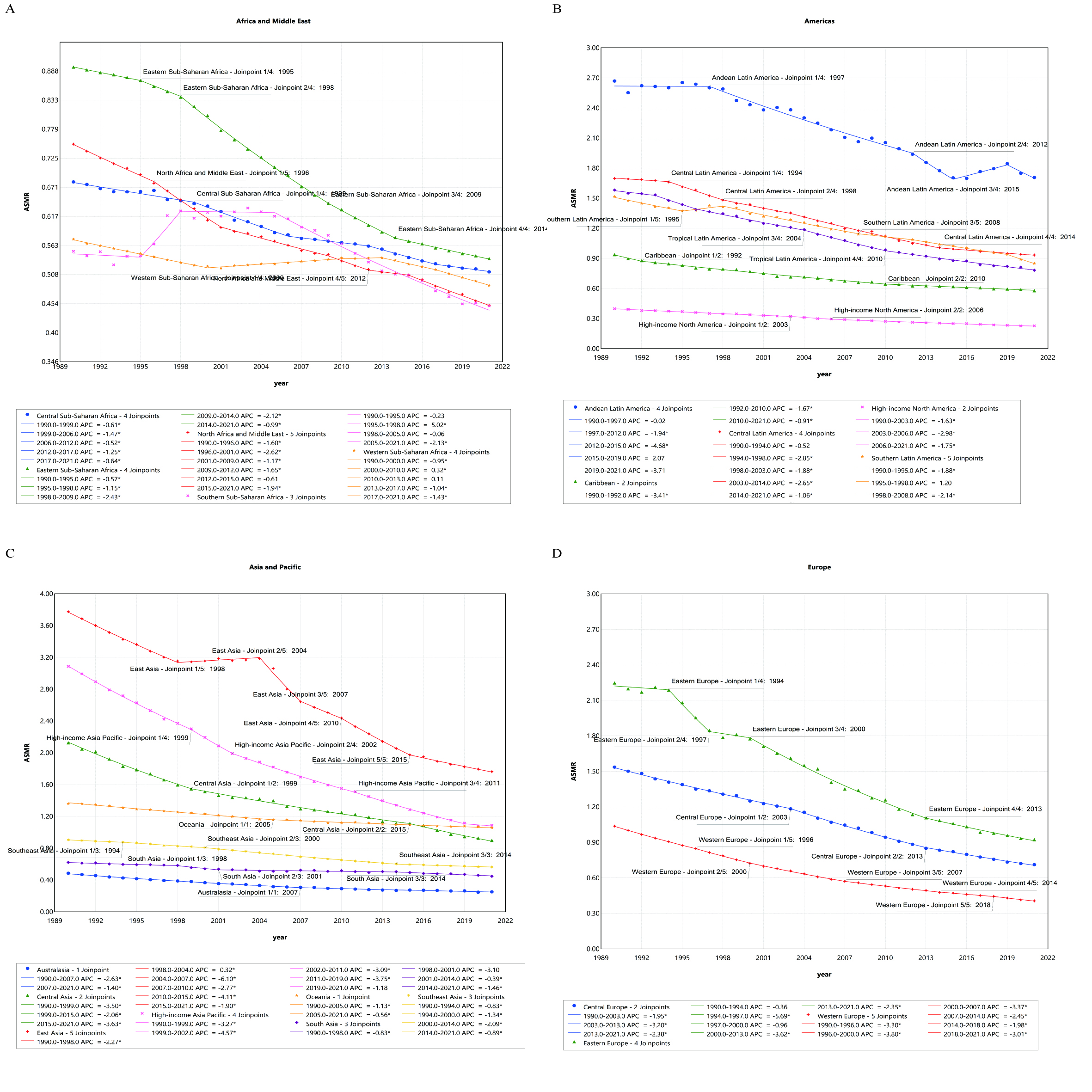

Supplement: Supplementary Figure 2 — Joinpoint regression analysis of age-standardized mortality rates (ASR) for high-sodium diet-attributable gastric cancer by sex and world regions, 1990–2021: (A) Africa and the Middle East; (B) Americas; (C) Asia-Pacific; (D) Europe. Abbreviation: ASR, age-standardized rate. [file Image_2.jpeg]

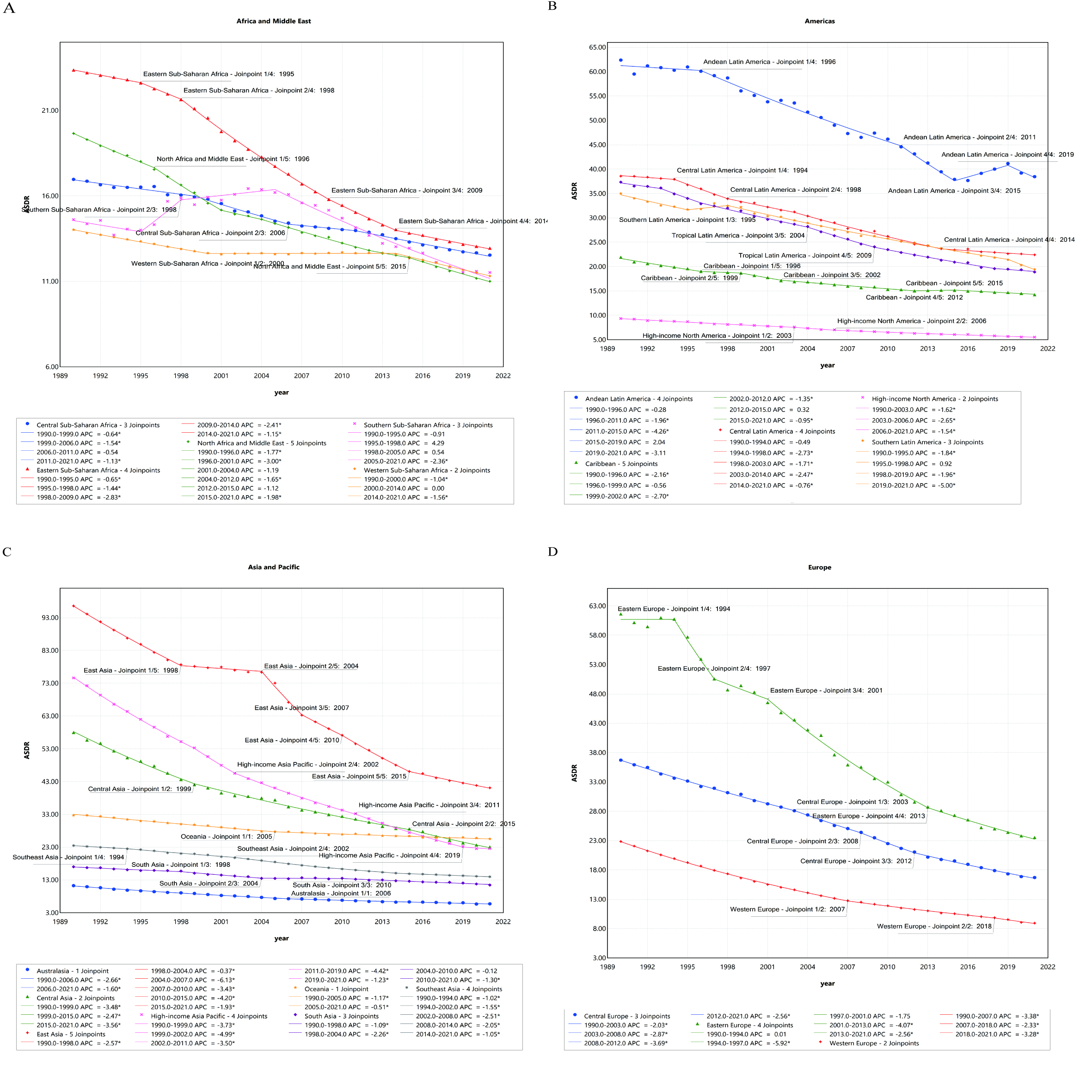

Supplement: Supplementary Figure 3 — Joinpoint regression analysis of age-standardized disability-adjusted life-year rates (ASR of DALYs) for high-sodium diet-attributable gastric cancer by sex and world regions, 1990–2021: (A) Africa and the Middle East; (B) Americas; (C) Asia-Pacific; (D) Europe. Abbreviations: ASR, age-standardized rate; DALYs, disability-adjusted life-years. [file Image_3.jpeg]

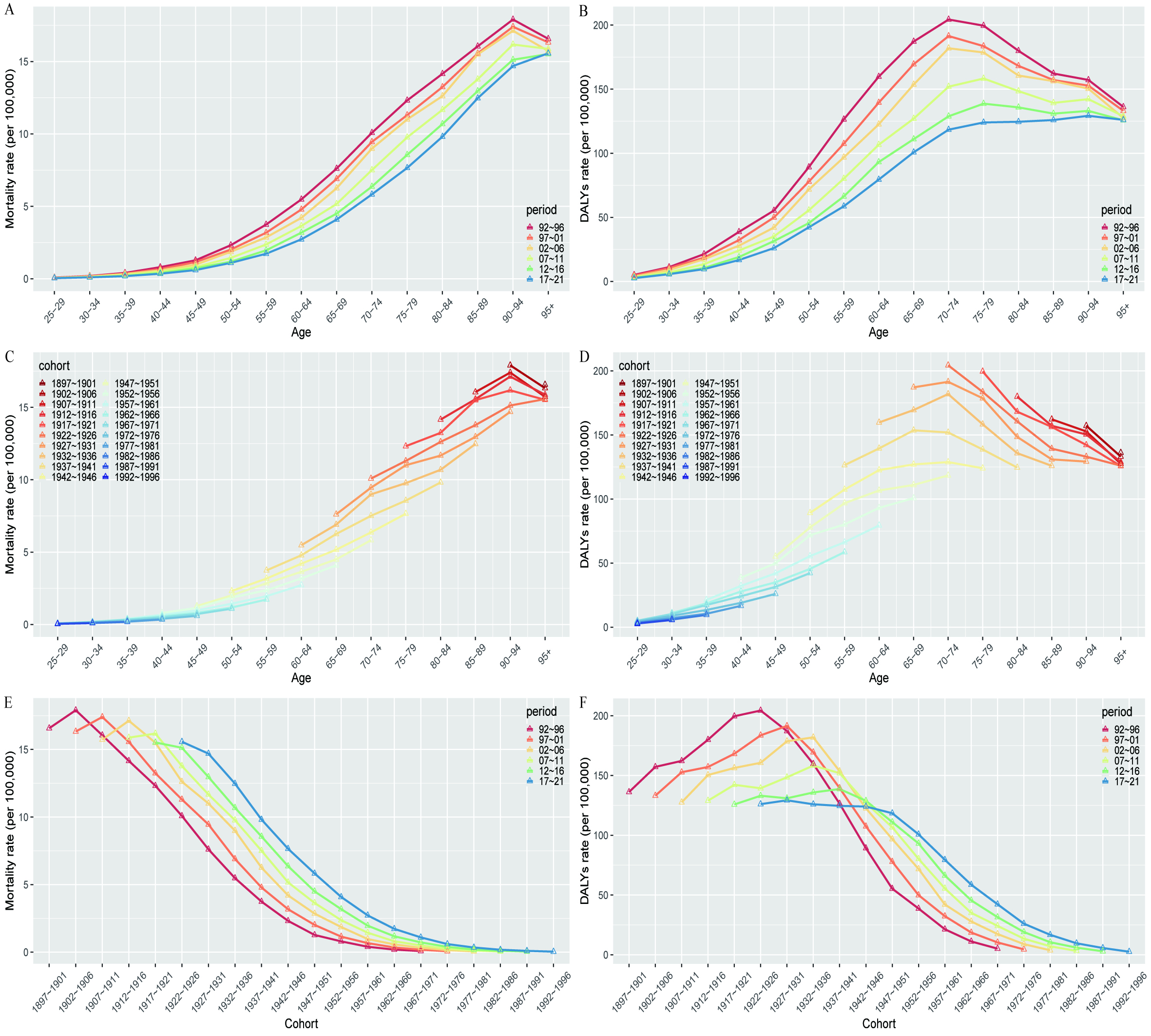

Supplement: Supplementary Figure 4 — Interaction effects between age, period, and cohort on high-sodium diet-attributable gastric cancer burden: (A, C, E) Interaction effects on mortality: age-period (A), age-cohort (C), and cohort-period (E); (B, D, F) Interaction effects on DALYs: age-period (B), age-cohort (D), and cohort-period (F). [file Image_4.jpeg]

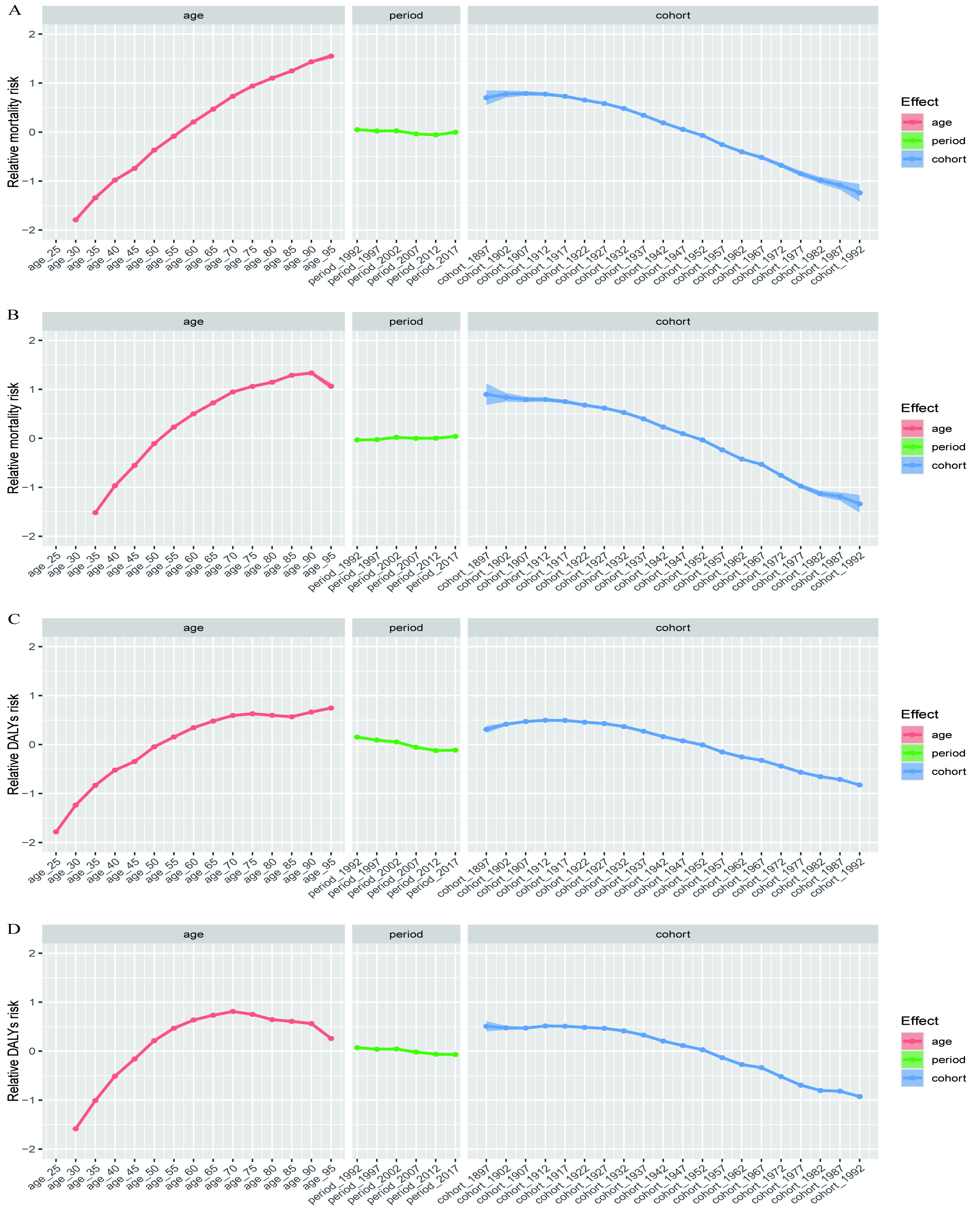

Supplement: Supplementary Figure 5 — Age-period-cohort effects on the relative risks of high-sodium diet-attributable gastric cancer, stratified by sex: (A) Mortality in females; (B) Mortality in males; (C) DALYs in females; (D) DALYs in males. [file Image_5.jpeg]

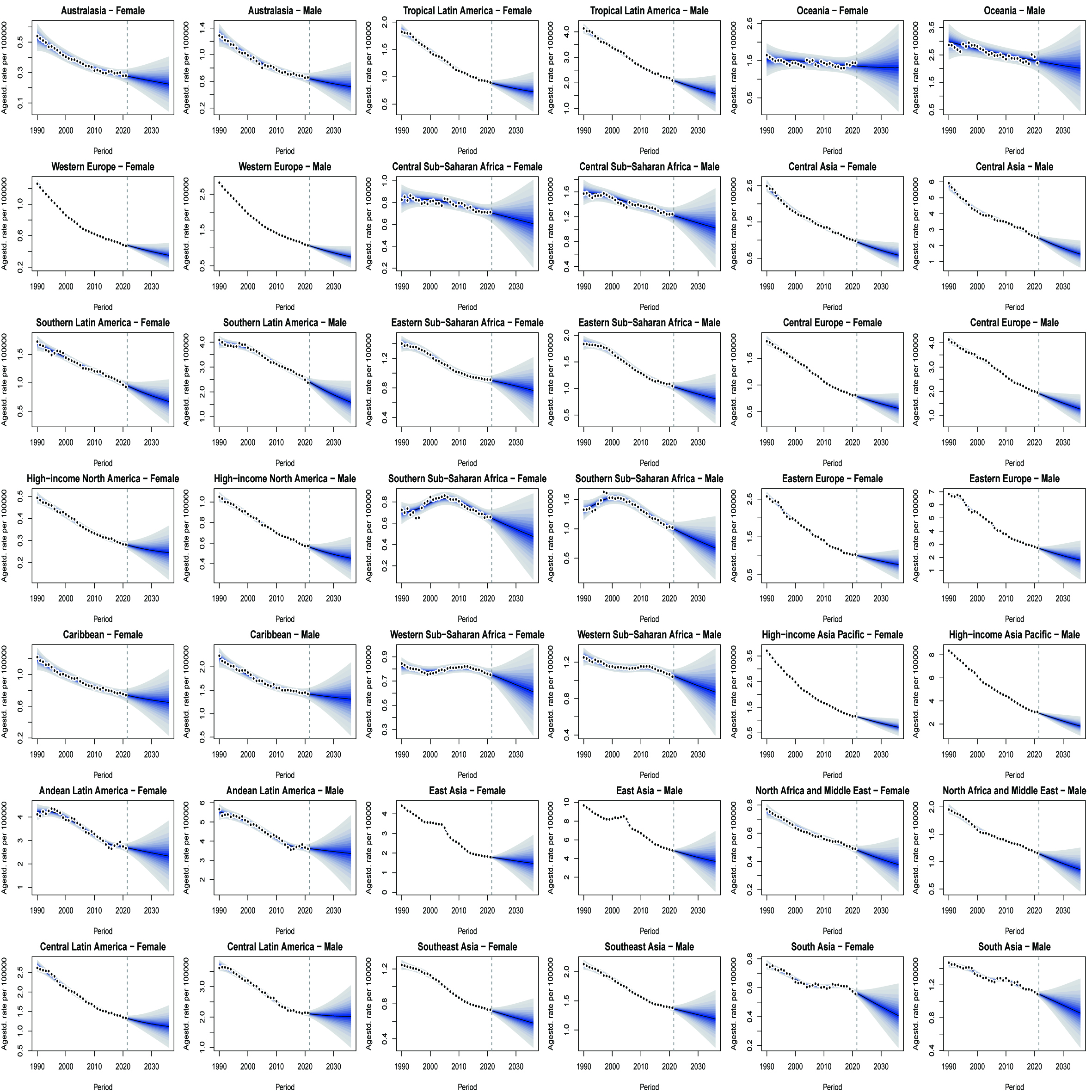

Supplement: Supplementary Figure 6 — Bayesian age-period-cohort (BAPC) model projections of high-sodium diet-attributable gastric cancer mortality in 21 Global Burden of Disease (GBD) regions by sex, 2022–2036. [file Image_6.jpeg]

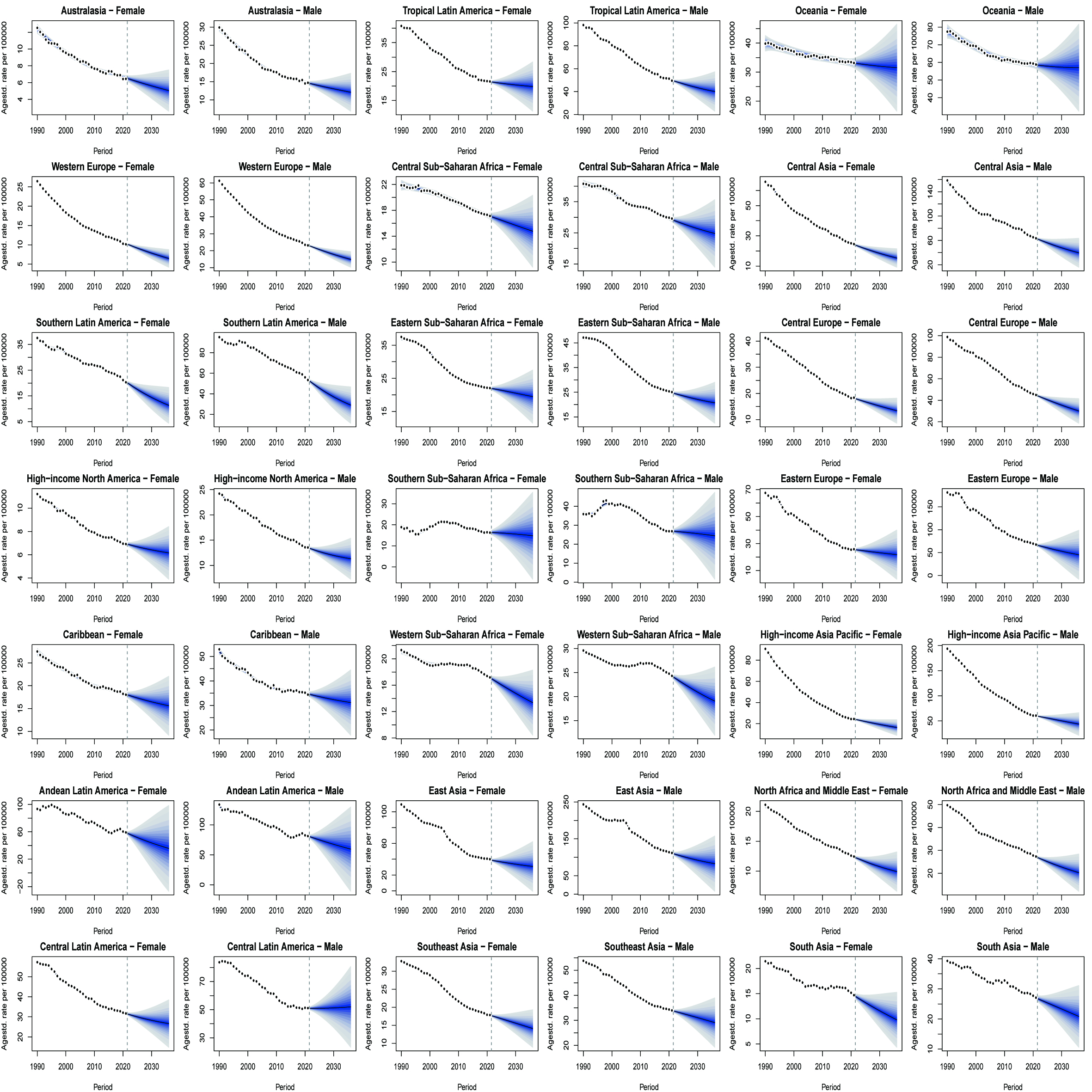

Supplement: Supplementary Figure 7 — Bayesian age-period-cohort (BAPC) model projections of high-sodium diet-attributable gastric cancer DALYs in 21 Global Burden of Disease (GBD) regions by sex, 2022–2036. [file Image_7.jpeg]

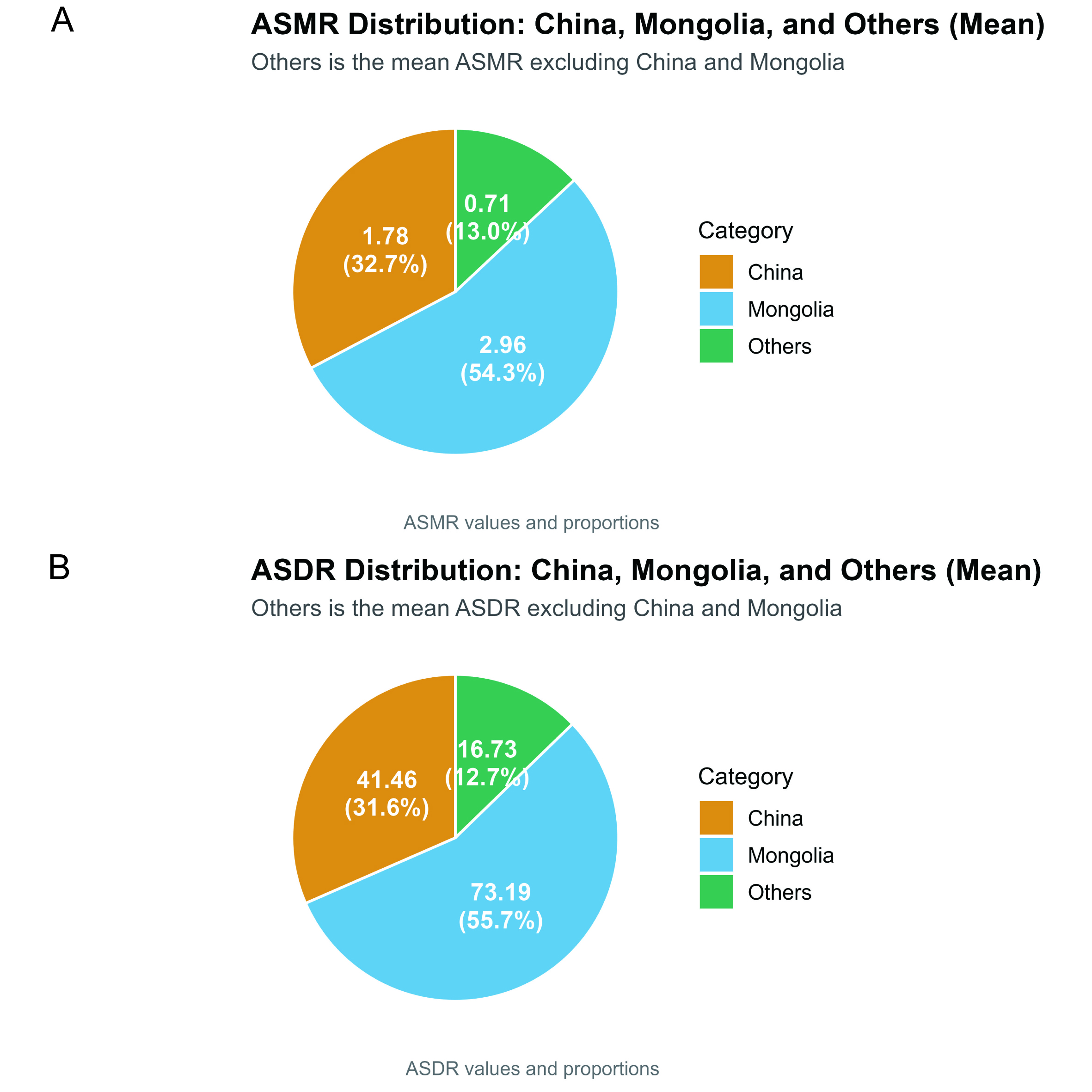

Supplement: Supplementary Figure 8 — (A) Pie chart illustrating the distribution of Age-Standardized Mortality Rates (ASMR) for China, Mongolia, and the mean of all other countries. (B) Pie chart illustrating the distribution of Age-Standardized DALYs Rates (ASDR) for China, Mongolia, and the mean of all other countries. [file Image_8.jpeg]
