# Supplementary material for: Global burden, trends, and inequalities of gastric cancer attributable to high-sodium diets: a 30-year analysis and projections based on the global burden of disease 2021 study
Source: Front Nutr. 2025 Dec 11;12:1683048. doi: 10.3389/fnut.2025.1683048 (PMC12739881; doi:10.3389/fnut.2025.1683048)
Supplement: Supplementary Table 1 — The case number and ASR of mortality and DALYs of gastric cancer attributable to high-sodium diet in 1990 and 2021 for female by SDI quintiles and by GBD regions, with EAPC from 1990 to 2021. ASR, age-standardized rate; DALYs, disability-adjusted life-years; SDI, sociodemographic index; GBD, Global Burden of Diseases, Injuries, and Risk Factors Study; EAPC, estimated annual percentage change; UIs, uncertainty intervals; CI, con?dence interval. [file Table_1.docx]

**Supplemental Table1. The case number and ASR of mortality and DALYs of gastric cancer attributable to high-sodium diet in 1990 and 2021 for female by SDI quintiles and by GBD regions, with EAPC from 1990 to 2021.**

|  | **Deaths** | | | | | **DALYs** | | | | |
| --- | --- | --- | --- | --- | --- | --- | --- | --- | --- | --- |
| **Location** | **Number (95 % UIs).1990** | **Number (95 % UIs).2021** | **ASR (95 % UIs) .1990** | **ASR (95 % UIs) .2021** | **EAPC (95 % CI) 1990–2021** | **Number (95 % UIs).1990** | **Number (95 % UIs).2021** | **ASR (95 % UIs) .1990** | **ASR (95 % UIs) .2021** | **EAPC (95 % CI) 1990–2021** |
| **Global** | **24202  (0 to 123378)** | **25287  (0 to 129118)** | **1.15  (0 to 5.86)** | **0.55  (0 to 2.79)** | **-2.57 (-2.67 to -2.48)** | **624558  (0 to 3176862)** | **573301  (0 to 2940974)** | **28.71  (0 to 146.18)** | **12.61  (0 to 64.62)** | **-2.84 (-2.93 to -2.74)** |
| **High SDI** | **5306  (0 to 27446)** | **4419  (0 to 23150)** | **0.81  (0 to 4.17)** | **0.33  (0 to 1.75)** | **-2.93 (-2.97 to -2.89)** | **115906  (0 to 595894)** | **76327  (0 to 396691)** | **19.37  (0 to 99.36)** | **7.13  (0 to 37.22)** | **-3.28 (-3.32 to -3.24)** |
| **High-middle SDI** | **8296  (0 to 42639)** | **7375  (0 to 37636)** | **1.49  (0 to 7.67)** | **0.67  (0 to 3.39)** | **-2.8 (-2.95 to -2.66)** | **208853  (0 to 1073034)** | **159994  (0 to 809417)** | **37.32  (0 to 191.67)** | **15.25  (0 to 76.89)** | **-3.11 (-3.27 to -2.96)** |
| **Middle SDI** | **8062  (0 to 40677)** | **9149  (0 to 46749)** | **1.57  (0 to 7.95)** | **0.67  (0 to 3.4)** | **-3.02 (-3.16 to -2.89)** | **222525  (0 to 1121025)** | **215896  (0 to 1098955)** | **39.15  (0 to 197.48)** | **15.17  (0 to 77.39)** | **-3.33 (-3.47 to -3.2)** |
| **Low-middle SDI** | **1773  (0 to 9045)** | **3111  (0 to 16125)** | **0.6  (0 to 3.07)** | **0.43  (0 to 2.22)** | **-1.01 (-1.05 to -0.97)** | **53666  (0 to 274524)** | **85118  (0 to 441724)** | **15.86  (0 to 81.05)** | **10.73  (0 to 55.56)** | **-1.19 (-1.22 to -1.15)** |
| **Low SDI** | **744  (0 to 3952)** | **1215  (0 to 6585)** | **0.67  (0 to 3.55)** | **0.48  (0 to 2.63)** | **-1.04 (-1.1 to -0.98)** | **23121  (0 to 123342)** | **35566  (0 to 193458)** | **18.05  (0 to 96.15)** | **12.12  (0 to 65.59)** | **-1.35 (-1.41 to -1.29)** |
| **High-income Asia Pacific** | **2261  (0 to 11173)** | **2128  (0 to 11055)** | **2.03  (0 to 10.03)** | **0.63  (0 to 3.23)** | **-3.82 (-3.88 to -3.76)** | **54887  (0 to 268264)** | **32366  (0 to 165738)** | **49.93  (0 to 243.45)** | **13.46  (0 to 67.47)** | **-4.28 (-4.33 to -4.22)** |
| **High-income North America** | **580  (0 to 3106)** | **560  (0 to 2984)** | **0.27  (0 to 1.44)** | **0.15  (0 to 0.82)** | **-1.88 (-1.94 to -1.83)** | **11907  (0 to 62478)** | **11757  (0 to 62005)** | **6.18  (0 to 32.31)** | **3.81  (0 to 19.86)** | **-1.59 (-1.65 to -1.53)** |
| **Western Europe** | **2527  (0 to 13792)** | **1530  (0 to 8522)** | **0.69  (0 to 3.76)** | **0.26  (0 to 1.42)** | **-3.13 (-3.24 to -3.01)** | **47281  (0 to 257747)** | **26151  (0 to 142626)** | **14.56  (0 to 78.68)** | **5.57  (0 to 30.05)** | **-3 (-3.09 to -2.91)** |
| **Australasia** | **40  (0 to 234)** | **47  (0 to 274)** | **0.3  (0 to 1.76)** | **0.16  (0 to 0.9)** | **-2.02 (-2.14 to -1.9)** | **859  (0 to 4976)** | **912  (0 to 5259)** | **6.91  (0 to 39.66)** | **3.55  (0 to 20.23)** | **-2.03 (-2.12 to -1.93)** |
| **Andean Latin America** | **227  (0 to 1144)** | **450  (0 to 2360)** | **2.25  (0 to 11.27)** | **1.47  (0 to 7.7)** | **-1.72 (-1.91 to -1.53)** | **5700  (0 to 28931)** | **10220  (0 to 53078)** | **51.41  (0 to 260.28)** | **32.55  (0 to 169.17)** | **-1.84 (-2.01 to -1.66)** |
| **Tropical Latin America** | **449  (0 to 2273)** | **698  (0 to 3624)** | **1  (0 to 5.07)** | **0.49  (0 to 2.56)** | **-2.46 (-2.53 to -2.39)** | **11327  (0 to 56944)** | **16760  (0 to 86631)** | **22.44  (0 to 113)** | **11.89  (0 to 61.46)** | **-2.25 (-2.32 to -2.19)** |
| **Central Latin America** | **570  (0 to 2897)** | **980  (0 to 5058)** | **1.43  (0 to 7.27)** | **0.73  (0 to 3.78)** | **-2.33 (-2.4 to -2.25)** | **14233  (0 to 72753)** | **23930  (0 to 124153)** | **31.49  (0 to 160.23)** | **17.46  (0 to 90.61)** | **-2.06 (-2.14 to -1.99)** |
| **Southern Latin America** | **241  (0 to 1205)** | **260  (0 to 1314)** | **0.95  (0 to 4.74)** | **0.5  (0 to 2.55)** | **-1.8 (-1.89 to -1.71)** | **5264  (0 to 26119)** | **5291  (0 to 26902)** | **20.64  (0 to 102.48)** | **11.17  (0 to 56.73)** | **-1.74 (-1.83 to -1.65)** |
| **Caribbean** | **86  (0 to 454)** | **118  (0 to 647)** | **0.66  (0 to 3.46)** | **0.41  (0 to 2.25)** | **-1.51 (-1.56 to -1.45)** | **2083  (0 to 11264)** | **2785  (0 to 15625)** | **15.15  (0 to 81.71)** | **9.94  (0 to 55.75)** | **-1.32 (-1.39 to -1.25)** |
| **Central Europe** | **848  (0 to 4266)** | **591  (0 to 2930)** | **1  (0 to 5.01)** | **0.44  (0 to 2.21)** | **-2.81 (-2.89 to -2.73)** | **19001  (0 to 95788)** | **11795  (0 to 58863)** | **22.73  (0 to 114.83)** | **10.12  (0 to 50.51)** | **-2.77 (-2.84 to -2.7)** |
| **Eastern Europe** | **2558  (0 to 13576)** | **1266  (0 to 6697)** | **1.42  (0 to 7.51)** | **0.57  (0 to 3.01)** | **-3.14 (-3.22 to -3.05)** | **63688  (0 to 339174)** | **28259  (0 to 148626)** | **37.26  (0 to 198.58)** | **14.24  (0 to 74.65)** | **-3.35 (-3.46 to -3.25)** |
| **Central Asia** | **379  (0 to 1921)** | **239  (0 to 1288)** | **1.37  (0 to 6.97)** | **0.54  (0 to 2.89)** | **-2.91 (-2.99 to -2.83)** | **10172  (0 to 51696)** | **6448  (0 to 34944)** | **36.29  (0 to 184.2)** | **13.53  (0 to 72.86)** | **-3.15 (-3.19 to -3.1)** |
| **North Africa and Middle East** | **359  (0 to 2316)** | **593  (0 to 3942)** | **0.42  (0 to 2.77)** | **0.27  (0 to 1.8)** | **-1.4 (-1.45 to -1.34)** | **11219  (0 to 71514)** | **17201  (0 to 113544)** | **11.64  (0 to 74.67)** | **6.86  (0 to 45.37)** | **-1.69 (-1.72 to -1.65)** |
| **South Asia** | **1182  (0 to 5987)** | **2294  (0 to 11957)** | **0.42  (0 to 2.13)** | **0.31  (0 to 1.6)** | **-0.82 (-0.96 to -0.68)** | **38466  (0 to 193293)** | **65497  (0 to 337524)** | **11.83  (0 to 59.74)** | **8.11  (0 to 41.92)** | **-1.07 (-1.2 to -0.93)** |
| **Southeast Asia** | **921  (0 to 4568)** | **1358  (0 to 6749)** | **0.69  (0 to 3.39)** | **0.4  (0 to 2)** | **-1.96 (-2.05 to -1.86)** | **27375  (0 to 137106)** | **35844  (0 to 179476)** | **18.07  (0 to 90.02)** | **9.83  (0 to 49)** | **-2.2 (-2.3 to -2.1)** |
| **East Asia** | **10363  (0 to 51031)** | **11114  (0 to 56593)** | **2.43  (0 to 11.9)** | **0.99  (0 to 5.01)** | **-3.18 (-3.4 to -2.96)** | **282874  (0 to 1399854)** | **248054  (0 to 1258794)** | **60.27  (0 to 297)** | **22.03  (0 to 111.89)** | **-3.56 (-3.77 to -3.34)** |
| **Oceania** | **12  (0 to 65)** | **25  (0 to 135)** | **0.95  (0 to 5.06)** | **0.78  (0 to 4.12)** | **-0.64 (-0.67 to -0.61)** | **341  (0 to 1992)** | **728  (0 to 4006)** | **22.03  (0 to 122.72)** | **18.26  (0 to 98.37)** | **-0.64 (-0.68 to -0.59)** |
| **Western Sub-Saharan Africa** | **195  (0 to 1045)** | **390  (0 to 2091)** | **0.46  (0 to 2.49)** | **0.41  (0 to 2.18)** | **-0.12 (-0.22 to -0.01)** | **5453  (0 to 29319)** | **10709  (0 to 57304)** | **11.76  (0 to 63.11)** | **9.46  (0 to 50.69)** | **-0.49 (-0.57 to -0.41)** |
| **Eastern Sub-Saharan Africa** | **290  (0 to 1462)** | **423  (0 to 2142)** | **0.77  (0 to 3.84)** | **0.5  (0 to 2.53)** | **-1.62 (-1.71 to -1.53)** | **9079  (0 to 46586)** | **12272  (0 to 62672)** | **20.67  (0 to 104.57)** | **12.16  (0 to 61.4)** | **-1.99 (-2.1 to -1.88)** |
| **Central Sub-Saharan Africa** | **53  (0 to 322)** | **108  (0 to 643)** | **0.48  (0 to 2.87)** | **0.39  (0 to 2.33)** | **-0.72 (-0.76 to -0.67)** | **1613  (0 to 9922)** | **3157  (0 to 18638)** | **12.03  (0 to 72.25)** | **9.45  (0 to 56.21)** | **-0.82 (-0.87 to -0.77)** |
| **Southern Sub-Saharan Africa** | **61  (0 to 334)** | **117  (0 to 644)** | **0.41  (0 to 2.22)** | **0.36  (0 to 2.01)** | **-0.12 (-0.46 to 0.21)** | **1735  (0 to 9416)** | **3164  (0 to 17129)** | **10.4  (0 to 56.27)** | **8.96  (0 to 48.74)** | **-0.04 (-0.41 to 0.33)** |

**Abbreviations: ASR, age-standardized rate; DALYs, disability-adjusted life-years; SDI, sociodemographic index; GBD, Global Burden of Diseases, Injuries, and Risk Factors Study; EAPC, estimated annual percentage change; UIs, uncertainty intervals; CI, conﬁdence interval.**

**Supplemental Table2. The case number and ASR of mortality and DALYs of Gastric cancer due to high sodium diet in 1990 and 2021 for Male by SDI quintiles and by GBD regions, with EAPC from 1990 to 2021.**

|  | **Deaths** | | | | | **DALYs** | | | | |
| --- | --- | --- | --- | --- | --- | --- | --- | --- | --- | --- |
| **Location** | **Number (95 % UIs).1990** | **Number (95 % UIs).2021** | **ASR (95 % UIs) .1990** | **ASR (95 % UIs) .2021** | **EAPC (95 % CI) 1990–2021** | **Number (95 % UIs).1990** | **Number (95 % UIs).2021** | **ASR (95 % UIs) .1990** | **ASR (95 % UIs) .2021** | **EAPC (95 % CI) 1990–2021** |
| **Global** | **43642  (0 to 220825)** | **50374  (0 to 247168)** | **2.46  (0 to 12.43)** | **1.29  (0 to 6.34)** | **-2.12 (-2.21 to -2.02)** | **1221058  (0 to 6151893)** | **1231290  (0 to 6026424)** | **62.2  (0 to 314.71)** | **29.9  (0 to 146.65)** | **-2.42 (-2.52 to -2.33)** |
| **High SDI** | **8400  (0 to 42507)** | **7790  (0 to 38610)** | **1.84  (0 to 9.32)** | **0.8  (0 to 3.98)** | **-2.76 (-2.8 to -2.72)** | **205590  (0 to 1042901)** | **154246  (0 to 764606)** | **43.22  (0 to 218.66)** | **16.75  (0 to 82.91)** | **-3.12 (-3.16 to -3.09)** |
| **High-middle SDI** | **15752  (0 to 79772)** | **16064  (0 to 79183)** | **3.72  (0 to 18.88)** | **1.83  (0 to 9.02)** | **-2.32 (-2.45 to -2.19)** | **444700  (0 to 2250084)** | **391402  (0 to 1933482)** | **95.57  (0 to 484.27)** | **43.01  (0 to 212.17)** | **-2.66 (-2.78 to -2.53)** |
| **Middle SDI** | **15188  (0 to 75359)** | **19667  (0 to 99399)** | **3.09  (0 to 15.42)** | **1.62  (0 to 8.12)** | **-2.14 (-2.28 to -2)** | **444814  (0 to 2193098)** | **496686  (0 to 2514966)** | **79.53  (0 to 395.11)** | **37.51  (0 to 189.67)** | **-2.48 (-2.61 to -2.35)** |
| **Low-middle SDI** | **3107  (0 to 15845)** | **5145  (0 to 25634)** | **1.02  (0 to 5.17)** | **0.77  (0 to 3.85)** | **-0.82 (-0.88 to -0.76)** | **91387  (0 to 469905)** | **141180  (0 to 705981)** | **26.72  (0 to 136.58)** | **19.15  (0 to 95.54)** | **-1.01 (-1.06 to -0.96)** |
| **Low SDI** | **1160  (0 to 6059)** | **1678  (0 to 8935)** | **1.04  (0 to 5.49)** | **0.72  (0 to 3.84)** | **-1.15 (-1.21 to -1.09)** | **33673  (0 to 175227)** | **47037  (0 to 251886)** | **26.69  (0 to 139.1)** | **17.42  (0 to 92.9)** | **-1.41 (-1.46 to -1.35)** |
| **High-income Asia Pacific** | **3798  (0 to 18686)** | **3736  (0 to 18472)** | **4.59  (0 to 22.71)** | **1.67  (0 to 8.29)** | **-3.35 (-3.4 to -3.3)** | **97051  (0 to 475786)** | **66651  (0 to 330302)** | **106.67  (0 to 524.47)** | **33.32  (0 to 164.65)** | **-3.83 (-3.89 to -3.77)** |
| **High-income North America** | **834  (0 to 4386)** | **922  (0 to 4667)** | **0.58  (0 to 3.03)** | **0.31  (0 to 1.59)** | **-2.03 (-2.08 to -1.98)** | **19729  (0 to 103534)** | **20874  (0 to 104704)** | **13.33  (0 to 69.88)** | **7.47  (0 to 37.38)** | **-1.9 (-1.94 to -1.86)** |
| **Western Europe** | **3605  (0 to 18570)** | **2520  (0 to 12812)** | **1.55  (0 to 7.98)** | **0.59  (0 to 2.98)** | **-3.12 (-3.22 to -3.03)** | **80255  (0 to 413504)** | **50019  (0 to 254190)** | **33.7  (0 to 173.38)** | **12.75  (0 to 64.63)** | **-3.09 (-3.18 to -3)** |
| **Australasia** | **74  (0 to 395)** | **91  (0 to 510)** | **0.73  (0 to 3.96)** | **0.36  (0 to 2)** | **-2.31 (-2.44 to -2.17)** | **1744  (0 to 9355)** | **1897  (0 to 10377)** | **16.45  (0 to 88.3)** | **8.1  (0 to 43.61)** | **-2.3 (-2.43 to -2.18)** |
| **Andean Latin America** | **295  (0 to 1480)** | **538  (0 to 2714)** | **3.12  (0 to 15.6)** | **1.97  (0 to 9.9)** | **-1.57 (-1.69 to -1.44)** | **7710  (0 to 39287)** | **12908  (0 to 64785)** | **73.84  (0 to 373.81)** | **44.84  (0 to 225.15)** | **-1.73 (-1.87 to -1.6)** |
| **Tropical Latin America** | **907  (0 to 4530)** | **1290  (0 to 6592)** | **2.26  (0 to 11.34)** | **1.15  (0 to 5.87)** | **-2.23 (-2.28 to -2.17)** | **24613  (0 to 122640)** | **32463  (0 to 165524)** | **54.08  (0 to 269.59)** | **27.29  (0 to 139.33)** | **-2.3 (-2.36 to -2.24)** |
| **Central Latin America** | **747  (0 to 3718)** | **1315  (0 to 6783)** | **1.98  (0 to 9.87)** | **1.17  (0 to 6.05)** | **-2.02 (-2.12 to -1.93)** | **19563  (0 to 97870)** | **33366  (0 to 171895)** | **46.12  (0 to 230.29)** | **28.16  (0 to 145.13)** | **-1.92 (-2.02 to -1.82)** |
| **Southern Latin America** | **445  (0 to 2274)** | **492  (0 to 2437)** | **2.25  (0 to 11.51)** | **1.3  (0 to 6.45)** | **-1.63 (-1.76 to -1.49)** | **11014  (0 to 56149)** | **11308  (0 to 55918)** | **52.39  (0 to 267.55)** | **29.43  (0 to 145.37)** | **-1.72 (-1.86 to -1.58)** |
| **Caribbean** | **150  (0 to 767)** | **193  (0 to 995)** | **1.24  (0 to 6.33)** | **0.77  (0 to 3.98)** | **-1.43 (-1.53 to -1.34)** | **3722  (0 to 19037)** | **4822  (0 to 24586)** | **29.15  (0 to 149.3)** | **18.99  (0 to 97.01)** | **-1.28 (-1.41 to -1.16)** |
| **Central Europe** | **1411  (0 to 7114)** | **1009  (0 to 4962)** | **2.28  (0 to 11.47)** | **1.07  (0 to 5.27)** | **-2.48 (-2.58 to -2.38)** | **36098  (0 to 181801)** | **23066  (0 to 113034)** | **54.47  (0 to 274.07)** | **24.77  (0 to 121.46)** | **-2.59 (-2.69 to -2.48)** |
| **Eastern Europe** | **3764  (0 to 19309)** | **1975  (0 to 10083)** | **3.76  (0 to 19.28)** | **1.5  (0 to 7.65)** | **-3.17 (-3.28 to -3.07)** | **110045  (0 to 562481)** | **50981  (0 to 259644)** | **100.13  (0 to 513.22)** | **37.25  (0 to 190.04)** | **-3.45 (-3.57 to -3.33)** |
| **Central Asia** | **619  (0 to 3058)** | **484  (0 to 2463)** | **3.26  (0 to 16.08)** | **1.39  (0 to 7.03)** | **-2.43 (-2.56 to -2.3)** | **18618  (0 to 91748)** | **13987  (0 to 71467)** | **87.55  (0 to 431.81)** | **35.04  (0 to 178.53)** | **-2.69 (-2.79 to -2.58)** |
| **North Africa and Middle East** | **898  (0 to 5061)** | **1384  (0 to 7986)** | **1.08  (0 to 6.12)** | **0.63  (0 to 3.69)** | **-1.65 (-1.71 to -1.58)** | **25805  (0 to 144346)** | **37558  (0 to 214862)** | **27.43  (0 to 153.92)** | **15.05  (0 to 86.79)** | **-1.89 (-1.97 to -1.81)** |
| **South Asia** | **2448  (0 to 12327)** | **4205  (0 to 20847)** | **0.81  (0 to 4.06)** | **0.6  (0 to 2.97)** | **-0.88 (-0.95 to -0.8)** | **74105  (0 to 374034)** | **115309  (0 to 568606)** | **21.69  (0 to 109.37)** | **14.93  (0 to 73.67)** | **-1.14 (-1.21 to -1.07)** |
| **Southeast Asia** | **1343  (0 to 6829)** | **2213  (0 to 11154)** | **1.17  (0 to 5.9)** | **0.76  (0 to 3.83)** | **-1.53 (-1.58 to -1.48)** | **39300  (0 to 200487)** | **61826  (0 to 312532)** | **29.67  (0 to 150.68)** | **18.72  (0 to 94.5)** | **-1.62 (-1.67 to -1.57)** |
| **East Asia** | **21453  (0 to 105109)** | **26748  (0 to 139322)** | **5.32  (0 to 26.25)** | **2.67  (0 to 13.86)** | **-2.22 (-2.43 to -2.01)** | **627292  (0 to 3080699)** | **658366  (0 to 3445706)** | **134.28  (0 to 656.88)** | **61.57  (0 to 320.67)** | **-2.56 (-2.74 to -2.37)** |
| **Oceania** | **24  (0 to 132)** | **47  (0 to 248)** | **1.76  (0 to 9.24)** | **1.32  (0 to 6.82)** | **-0.96 (-1.03 to -0.89)** | **723  (0 to 4063)** | **1416  (0 to 7609)** | **42.74  (0 to 233.5)** | **32.33  (0 to 170.89)** | **-0.93 (-1.01 to -0.85)** |
| **Western Sub-Saharan Africa** | **284  (0 to 1495)** | **490  (0 to 2535)** | **0.69  (0 to 3.69)** | **0.57  (0 to 2.95)** | **-0.4 (-0.48 to -0.32)** | **7717  (0 to 40548)** | **13393  (0 to 69776)** | **16.31  (0 to 85.87)** | **13.4  (0 to 69.37)** | **-0.41 (-0.5 to -0.33)** |
| **Eastern Sub-Saharan Africa** | **367  (0 to 1877)** | **429  (0 to 2311)** | **1.02  (0 to 5.23)** | **0.58  (0 to 3.1)** | **-2.1 (-2.21 to -1.99)** | **10637  (0 to 54359)** | **12198  (0 to 66145)** | **26.01  (0 to 132.6)** | **13.81  (0 to 74.49)** | **-2.36 (-2.48 to -2.25)** |
| **Central Sub-Saharan Africa** | **89  (0 to 522)** | **154  (0 to 889)** | **0.91  (0 to 5.32)** | **0.68  (0 to 3.94)** | **-1.02 (-1.07 to -0.96)** | **2623  (0 to 15124)** | **4706  (0 to 27151)** | **22.57  (0 to 131.93)** | **16.31  (0 to 94.26)** | **-1.12 (-1.18 to -1.07)** |
| **Southern Sub-Saharan Africa** | **87  (0 to 469)** | **137  (0 to 751)** | **0.74  (0 to 4.06)** | **0.57  (0 to 3.17)** | **-1.07 (-1.42 to -0.72)** | **2696  (0 to 14266)** | **4176  (0 to 22632)** | **19.79  (0 to 106.15)** | **14.8  (0 to 80.6)** | **-1.18 (-1.55 to -0.81)** |

**Abbreviations: ASR, age-standardized rate; DALYs, disability-adjusted life-years; SDI, sociodemographic index; GBD, Global Burden of Diseases, Injuries, and Risk Factors Study; EAPC, estimated annual percentage change; UIs, uncertainty intervals; CI, conﬁdence interval.**

**Supplemental Table3. The case number and ASR of mortality and DALYs of gastric cancer attributable to high-sodium diet in 1990 and 2021 for both sexes by SDI quintiles, by GBD regions and 204 countries and regions, with EAPC from 1990 to 2021.**

|  | **SDI** | | **Deaths** | | | | | **DALYs** | | | | |
| --- | --- | --- | --- | --- | --- | --- | --- | --- | --- | --- | --- | --- |
| **Location** | **SDI.Index.Value** | **SDI.Quintile** | **Number (95 % UIs).1990** | **Number (95 % UIs).2021** | **ASR (95 % UIs) .1990** | **ASR (95 % UIs) .2021** | **EAPC (95 % CI) 1990–2021** | **Number (95 % UIs).1990** | **Number (95 % UIs).2021** | **ASR (95 % UIs) .1990** | **ASR (95 % UIs) .2021** | **EAPC (95 % CI) 1990–2021** |
| **Global** | **0.666368** | **NA** | **67845 (0 to 339513)** | **75661 (0 to 372194)** | **1.74 (0 to 8.74)** | **0.89 (0 to 4.37)** | **-2.26 (-2.35 to -2.18)** | **1845617 (0 to 9206158)** | **1804592 (0 to 8884379)** | **44.53 (0 to 222.31)** | **20.78 (0 to 102.38)** | **-2.56 (-2.64 to -2.47)** |
| **High SDI** | **NA** | **NA** | **13706 (0 to 69403)** | **12209 (0 to 61722)** | **1.24 (0 to 6.27)** | **0.54 (0 to 2.73)** | **-2.72 (-2.75 to -2.7)** | **321496 (0 to 1625678)** | **230574 (0 to 1154960)** | **29.89 (0 to 151.09)** | **11.6 (0 to 58.09)** | **-3.11 (-3.13 to -3.08)** |
| **High-middle SDI** | **NA** | **NA** | **24048 (0 to 122047)** | **23439 (0 to 114553)** | **2.44 (0 to 12.41)** | **1.18 (0 to 5.79)** | **-2.43 (-2.56 to -2.31)** | **653552 (0 to 3320830)** | **551396 (0 to 2681101)** | **63.6 (0 to 322.96)** | **28.14 (0 to 136.92)** | **-2.76 (-2.9 to -2.63)** |
| **Middle SDI** | **NA** | **NA** | **23250 (0 to 115303)** | **28816 (0 to 141923)** | **2.3 (0 to 11.48)** | **1.11 (0 to 5.43)** | **-2.47 (-2.6 to -2.35)** | **667339 (0 to 3270306)** | **712582 (0 to 3527047)** | **59.02 (0 to 290.91)** | **25.82 (0 to 127.58)** | **-2.79 (-2.91 to -2.68)** |
| **Low-middle SDI** | **NA** | **NA** | **4881 (0 to 24751)** | **8255 (0 to 41816)** | **0.82 (0 to 4.11)** | **0.59 (0 to 3)** | **-0.95 (-1 to -0.91)** | **145053 (0 to 737564)** | **226298 (0 to 1148265)** | **21.39 (0 to 108.46)** | **14.78 (0 to 75.09)** | **-1.13 (-1.16 to -1.09)** |
| **Low SDI** | **NA** | **NA** | **1904 (0 to 9916)** | **2894 (0 to 15443)** | **0.86 (0 to 4.48)** | **0.6 (0 to 3.18)** | **-1.14 (-1.19 to -1.08)** | **56794 (0 to 295505)** | **82603 (0 to 441281)** | **22.47 (0 to 116.97)** | **14.71 (0 to 78.41)** | **-1.41 (-1.47 to -1.36)** |
| **East Asia** | **0.722912** | **NA** | **31816 (0 to 155224)** | **37862 (0 to 188112)** | **3.77 (0 to 18.42)** | **1.76 (0 to 8.69)** | **-2.54 (-2.74 to -2.34)** | **910166 (0 to 4421005)** | **906420 (0 to 4574158)** | **96.58 (0 to 469.07)** | **41.09 (0 to 206.63)** | **-2.88 (-3.06 to -2.7)** |
| **China** | **0.718679** | **NA** | **31208 (0 to 152476)** | **36958 (0 to 183972)** | **3.85 (0 to 18.79)** | **1.78 (0 to 8.81)** | **-2.56 (-2.77 to -2.35)** | **892815 (0 to 4340140)** | **883435 (0 to 4461211)** | **98.4 (0 to 478.5)** | **41.46 (0 to 208.59)** | **-2.91 (-3.1 to -2.72)** |
| **Democratic People's Republic of Korea** | **0.569455** | **Low-middle SDI** | **367 (0 to 1839)** | **594 (0 to 3058)** | **2.3 (0 to 11.52)** | **1.8 (0 to 9.26)** | **-0.79 (-0.91 to -0.67)** | **10913 (0 to 54775)** | **16585 (0 to 85026)** | **61.06 (0 to 305.53)** | **48.47 (0 to 249.15)** | **-0.77 (-0.88 to -0.66)** |
| **Taiwan (Province of China)** | **0.87514** | **High SDI** | **241 (0 to 1211)** | **310 (0 to 1569)** | **1.59 (0 to 7.97)** | **0.72 (0 to 3.66)** | **-3 (-3.22 to -2.77)** | **6439 (0 to 32662)** | **6400 (0 to 32826)** | **38.17 (0 to 193.09)** | **15.52 (0 to 79.6)** | **-3.3 (-3.49 to -3.11)** |
| **Southeast Asia** | **0.649072** | **NA** | **2264 (0 to 11385)** | **3572 (0 to 18178)** | **0.91 (0 to 4.61)** | **0.56 (0 to 2.89)** | **-1.71 (-1.78 to -1.65)** | **66676 (0 to 336540)** | **97670 (0 to 497997)** | **23.49 (0 to 118.14)** | **13.98 (0 to 71.25)** | **-1.85 (-1.91 to -1.78)** |
| **Cambodia** | **0.474** | **Low-middle SDI** | **62 (0 to 327)** | **101 (0 to 502)** | **1.39 (0 to 7.28)** | **0.86 (0 to 4.27)** | **-1.69 (-1.79 to -1.59)** | **1867 (0 to 10016)** | **2814 (0 to 13956)** | **36.7 (0 to 195.64)** | **21.06 (0 to 104.42)** | **-1.98 (-2.08 to -1.88)** |
| **Indonesia** | **0.657935** | **NA** | **765 (0 to 3955)** | **1353 (0 to 6848)** | **0.8 (0 to 4.08)** | **0.61 (0 to 3.11)** | **-0.78 (-0.85 to -0.71)** | **23391 (0 to 122084)** | **37686 (0 to 189057)** | **20.82 (0 to 107.86)** | **14.58 (0 to 73.62)** | **-1.11 (-1.18 to -1.04)** |
| **Lao People's Democratic Republic** | **0.489281** | **Low-middle SDI** | **30 (0 to 155)** | **31 (0 to 158)** | **1.44 (0 to 7.51)** | **0.71 (0 to 3.57)** | **-2.49 (-2.57 to -2.41)** | **903 (0 to 4721)** | **892 (0 to 4530)** | **38.99 (0 to 203.26)** | **17.38 (0 to 88.01)** | **-2.81 (-2.9 to -2.73)** |
| **Malaysia** | **0.742553** | **High-middle SDI** | **66 (0 to 329)** | **141 (0 to 713)** | **0.74 (0 to 3.66)** | **0.53 (0 to 2.67)** | **-1.29 (-1.43 to -1.16)** | **1810 (0 to 8919)** | **3613 (0 to 18387)** | **18.2 (0 to 90.4)** | **12.29 (0 to 62.33)** | **-1.41 (-1.56 to -1.27)** |
| **Maldives** | **0.657665** | **Middle SDI** | **1 (0 to 3)** | **1 (0 to 4)** | **0.8 (0 to 4.05)** | **0.23 (0 to 1.18)** | **-4.37 (-4.59 to -4.14)** | **21 (0 to 108)** | **20 (0 to 101)** | **20.52 (0 to 105.03)** | **5.06 (0 to 25.89)** | **-4.94 (-5.2 to -4.68)** |
| **Myanmar** | **0.528492** | **Low-middle SDI** | **301 (0 to 1562)** | **285 (0 to 1417)** | **1.31 (0 to 6.84)** | **0.61 (0 to 3.02)** | **-2.83 (-2.96 to -2.69)** | **9092 (0 to 47681)** | **7695 (0 to 39007)** | **35.23 (0 to 183.22)** | **14.98 (0 to 75.45)** | **-3.16 (-3.3 to -3.01)** |
| **Philippines** | **0.65192** | **NA** | **132 (0 to 663)** | **289 (0 to 1486)** | **0.47 (0 to 2.34)** | **0.36 (0 to 1.86)** | **-0.64 (-0.72 to -0.55)** | **4082 (0 to 20610)** | **8288 (0 to 42712)** | **11.82 (0 to 59.59)** | **9.21 (0 to 47.21)** | **-0.72 (-0.81 to -0.64)** |
| **Sri Lanka** | **0.701372** | **Middle SDI** | **74 (0 to 360)** | **78 (0 to 407)** | **0.73 (0 to 3.59)** | **0.29 (0 to 1.54)** | **-3.03 (-3.24 to -2.82)** | **2067 (0 to 10071)** | **1962 (0 to 10183)** | **17.66 (0 to 86.39)** | **7.18 (0 to 37.42)** | **-3.06 (-3.3 to -2.83)** |
| **Thailand** | **0.682657** | **Middle SDI** | **328 (0 to 1637)** | **638 (0 to 3388)** | **0.95 (0 to 4.72)** | **0.6 (0 to 3.18)** | **-1.87 (-2.02 to -1.72)** | **9566 (0 to 48203)** | **16593 (0 to 88976)** | **24.17 (0 to 121.39)** | **16.02 (0 to 85.4)** | **-1.73 (-1.9 to -1.55)** |
| **Timor-Leste** | **0.450689** | **Low SDI** | **2 (0 to 12)** | **5 (0 to 27)** | **0.88 (0 to 4.64)** | **0.64 (0 to 3.31)** | **-0.93 (-1.16 to -0.69)** | **76 (0 to 401)** | **136 (0 to 702)** | **22.35 (0 to 116.99)** | **15.32 (0 to 78.81)** | **-1.15 (-1.43 to -0.87)** |
| **Viet Nam** | **0.621621** | **Middle SDI** | **490 (0 to 2475)** | **630 (0 to 3106)** | **1.23 (0 to 6.16)** | **0.64 (0 to 3.15)** | **-2.49 (-2.69 to -2.29)** | **13434 (0 to 68382)** | **17445 (0 to 87541)** | **32 (0 to 162.06)** | **16.32 (0 to 81.42)** | **-2.5 (-2.68 to -2.31)** |
| **Oceania** | **0.467359** | **NA** | **36 (0 to 196)** | **72 (0 to 381)** | **1.36 (0 to 7.12)** | **1.06 (0 to 5.52)** | **-0.83 (-0.89 to -0.78)** | **1065 (0 to 5908)** | **2144 (0 to 11558)** | **32.71 (0 to 178.43)** | **25.52 (0 to 134.98)** | **-0.82 (-0.89 to -0.76)** |
| **Fiji** | **0.669069** | **Middle SDI** | **3 (0 to 14)** | **4 (0 to 23)** | **0.81 (0 to 4.4)** | **0.65 (0 to 3.47)** | **-0.78 (-0.94 to -0.61)** | **76 (0 to 436)** | **115 (0 to 628)** | **19.08 (0 to 106.74)** | **14.67 (0 to 79.24)** | **-0.83 (-1.02 to -0.64)** |
| **Kiribati** | **0.525958** | **Low-middle SDI** | **1 (0 to 3)** | **1 (0 to 6)** | **1.89 (0 to 9.73)** | **1.73 (0 to 8.99)** | **-0.29 (-0.34 to -0.23)** | **20 (0 to 107)** | **35 (0 to 189)** | **47.5 (0 to 253.3)** | **42.64 (0 to 225.77)** | **-0.35 (-0.41 to -0.28)** |
| **Marshall Islands** | **0.573525** | **Low-middle SDI** | **0 (0 to 1)** | **0 (0 to 2)** | **1.82 (0 to 9.37)** | **1.4 (0 to 7.27)** | **-0.76 (-0.84 to -0.67)** | **8 (0 to 44)** | **14 (0 to 75)** | **44.72 (0 to 232.18)** | **34.9 (0 to 184.28)** | **-0.74 (-0.83 to -0.64)** |
| **Micronesia (Federated States of)** | **0.588013** | **Low-middle SDI** | **1 (0 to 4)** | **1 (0 to 5)** | **1.79 (0 to 9.35)** | **1.35 (0 to 6.9)** | **-0.94 (-1.03 to -0.85)** | **23 (0 to 126)** | **27 (0 to 143)** | **44.15 (0 to 237.28)** | **33.3 (0 to 175.38)** | **-0.93 (-1.03 to -0.84)** |
| **Papua New Guinea** | **0.418098** | **Low SDI** | **23 (0 to 127)** | **51 (0 to 276)** | **1.44 (0 to 7.6)** | **1.13 (0 to 5.96)** | **-0.79 (-0.84 to -0.75)** | **695 (0 to 3899)** | **1530 (0 to 8512)** | **34.69 (0 to 189.6)** | **26.74 (0 to 145.56)** | **-0.87 (-0.92 to -0.82)** |
| **Samoa** | **0.59234** | **Low-middle SDI** | **1 (0 to 3)** | **1 (0 to 5)** | **0.75 (0 to 4.36)** | **0.65 (0 to 3.77)** | **-0.54 (-0.63 to -0.45)** | **14 (0 to 86)** | **21 (0 to 125)** | **16.43 (0 to 99.62)** | **14.28 (0 to 85.36)** | **-0.49 (-0.6 to -0.38)** |
| **Solomon Islands** | **0.429542** | **Low SDI** | **2 (0 to 13)** | **5 (0 to 26)** | **1.92 (0 to 10.34)** | **1.52 (0 to 7.89)** | **-0.71 (-0.82 to -0.6)** | **72 (0 to 415)** | **155 (0 to 848)** | **47.35 (0 to 266.68)** | **38.27 (0 to 203.86)** | **-0.63 (-0.75 to -0.51)** |
| **Tonga** | **0.629101** | **Middle SDI** | **1 (0 to 4)** | **1 (0 to 5)** | **1.65 (0 to 8.68)** | **1.33 (0 to 6.72)** | **-0.62 (-0.74 to -0.5)** | **21 (0 to 116)** | **25 (0 to 130)** | **37.87 (0 to 202.85)** | **30.56 (0 to 159.75)** | **-0.63 (-0.73 to -0.52)** |
| **Vanuatu** | **0.472796** | **Low-middle SDI** | **1 (0 to 5)** | **2 (0 to 11)** | **1.62 (0 to 8.46)** | **1.29 (0 to 6.63)** | **-0.87 (-0.92 to -0.82)** | **27 (0 to 153)** | **61 (0 to 333)** | **39.13 (0 to 213.73)** | **31.59 (0 to 167.94)** | **-0.85 (-0.91 to -0.78)** |
| **Central Asia** | **0.674963** | **NA** | **998 (0 to 4972)** | **723 (0 to 3725)** | **2.13 (0 to 10.58)** | **0.9 (0 to 4.6)** | **-2.54 (-2.64 to -2.44)** | **28790 (0 to 143224)** | **20435 (0 to 105882)** | **57.97 (0 to 288.43)** | **23.08 (0 to 119.44)** | **-2.8 (-2.88 to -2.73)** |
| **Armenia** | **0.702497** | **Middle SDI** | **48 (0 to 238)** | **36 (0 to 190)** | **1.76 (0 to 8.76)** | **0.84 (0 to 4.37)** | **-2.01 (-2.21 to -1.81)** | **1386 (0 to 6883)** | **855 (0 to 4465)** | **47.39 (0 to 234.69)** | **20.07 (0 to 104.48)** | **-2.53 (-2.73 to -2.33)** |
| **Azerbaijan** | **0.695411** | **Middle SDI** | **103 (0 to 512)** | **109 (0 to 580)** | **2.07 (0 to 10.26)** | **1.12 (0 to 5.95)** | **-1.94 (-2.09 to -1.8)** | **3021 (0 to 14868)** | **2983 (0 to 15762)** | **55.91 (0 to 276.11)** | **27.11 (0 to 143.45)** | **-2.45 (-2.59 to -2.31)** |
| **Georgia** | **0.733124** | **High-middle SDI** | **88 (0 to 434)** | **55 (0 to 281)** | **1.4 (0 to 6.95)** | **0.92 (0 to 4.72)** | **-0.31 (-0.77 to 0.16)** | **2406 (0 to 11946)** | **1282 (0 to 6631)** | **38.06 (0 to 188.76)** | **22.66 (0 to 117.21)** | **-0.69 (-1.11 to -0.27)** |
| **Kazakhstan** | **0.718332** | **High-middle SDI** | **346 (0 to 1714)** | **149 (0 to 778)** | **2.73 (0 to 13.55)** | **0.84 (0 to 4.36)** | **-3.65 (-3.85 to -3.45)** | **9938 (0 to 49316)** | **4153 (0 to 21561)** | **73.78 (0 to 365.38)** | **21.61 (0 to 112.23)** | **-3.82 (-4.01 to -3.64)** |
| **Kyrgyzstan** | **0.609181** | **Low-middle SDI** | **76 (0 to 375)** | **58 (0 to 302)** | **2.55 (0 to 12.58)** | **1.19 (0 to 6.19)** | **-2.19 (-2.4 to -1.98)** | **2278 (0 to 11235)** | **1707 (0 to 8951)** | **72.74 (0 to 359.43)** | **31.59 (0 to 165.39)** | **-2.48 (-2.66 to -2.31)** |
| **Mongolia** | **0.618744** | **Low-middle SDI** | **47 (0 to 230)** | **65 (0 to 340)** | **4.52 (0 to 22.13)** | **2.96 (0 to 15.68)** | **-1.77 (-1.92 to -1.62)** | **1297 (0 to 6470)** | **1904 (0 to 9843)** | **116.56 (0 to 576.28)** | **73.19 (0 to 380.58)** | **-1.89 (-2.06 to -1.73)** |
| **Tajikistan** | **0.536613** | **Low-middle SDI** | **63 (0 to 317)** | **62 (0 to 333)** | **2.32 (0 to 11.6)** | **1.12 (0 to 6)** | **-2.24 (-2.49 to -1.99)** | **1823 (0 to 9210)** | **1864 (0 to 9973)** | **62.3 (0 to 314.16)** | **28.12 (0 to 150.18)** | **-2.57 (-2.76 to -2.38)** |
| **Turkmenistan** | **0.68304** | **Middle SDI** | **34 (0 to 168)** | **30 (0 to 159)** | **1.78 (0 to 8.68)** | **0.74 (0 to 3.9)** | **-2.85 (-3.13 to -2.56)** | **990 (0 to 4877)** | **901 (0 to 4763)** | **47.49 (0 to 234.31)** | **20.09 (0 to 105.89)** | **-2.84 (-3.13 to -2.55)** |
| **Uzbekistan** | **0.664965** | **Middle SDI** | **193 (0 to 973)** | **158 (0 to 805)** | **1.67 (0 to 8.4)** | **0.59 (0 to 3.03)** | **-3.01 (-3.2 to -2.82)** | **5651 (0 to 28368)** | **4785 (0 to 24425)** | **46.42 (0 to 232.81)** | **15.99 (0 to 81.55)** | **-3.18 (-3.36 to -3)** |
| **Central Europe** | **0.79578** | **NA** | **2259 (0 to 11339)** | **1600 (0 to 7858)** | **1.54 (0 to 7.69)** | **0.71 (0 to 3.49)** | **-2.58 (-2.67 to -2.5)** | **55099 (0 to 277909)** | **34860 (0 to 170909)** | **36.72 (0 to 185.31)** | **16.71 (0 to 81.88)** | **-2.62 (-2.71 to -2.54)** |
| **Albania** | **0.706889** | **Middle SDI** | **28 (0 to 143)** | **38 (0 to 196)** | **1.47 (0 to 7.46)** | **0.88 (0 to 4.51)** | **-1.56 (-1.8 to -1.31)** | **716 (0 to 3578)** | **809 (0 to 4220)** | **33.44 (0 to 168.87)** | **19.05 (0 to 99.74)** | **-1.7 (-1.94 to -1.47)** |
| **Bosnia and Herzegovina** | **0.722964** | **High-middle SDI** | **45 (0 to 222)** | **46 (0 to 236)** | **1.15 (0 to 5.63)** | **0.73 (0 to 3.72)** | **-1.67 (-1.83 to -1.5)** | **1220 (0 to 5919)** | **1007 (0 to 5169)** | **28.17 (0 to 137.3)** | **16.63 (0 to 84.94)** | **-1.89 (-2.06 to -1.73)** |
| **Bulgaria** | **0.764641** | **High-middle SDI** | **222 (0 to 1104)** | **135 (0 to 662)** | **1.88 (0 to 9.36)** | **0.94 (0 to 4.63)** | **-1.91 (-2.1 to -1.73)** | **5508 (0 to 27298)** | **2988 (0 to 14705)** | **44.89 (0 to 222.56)** | **22.76 (0 to 112.13)** | **-1.94 (-2.11 to -1.76)** |
| **Croatia** | **0.799069** | **High-middle SDI** | **117 (0 to 588)** | **66 (0 to 330)** | **2.01 (0 to 10.08)** | **0.71 (0 to 3.53)** | **-3.29 (-3.4 to -3.17)** | **2762 (0 to 14013)** | **1324 (0 to 6553)** | **45.24 (0 to 228.51)** | **15.64 (0 to 77.58)** | **-3.4 (-3.51 to -3.29)** |
| **Czechia** | **0.82851** | **High SDI** | **198 (0 to 982)** | **102 (0 to 504)** | **1.42 (0 to 7.02)** | **0.46 (0 to 2.26)** | **-3.85 (-3.95 to -3.75)** | **4405 (0 to 21834)** | **2088 (0 to 10355)** | **32.21 (0 to 159.49)** | **10.23 (0 to 50.64)** | **-3.79 (-3.86 to -3.72)** |
| **Hungary** | **0.791025** | **High-middle SDI** | **248 (0 to 1223)** | **117 (0 to 575)** | **1.69 (0 to 8.38)** | **0.59 (0 to 2.9)** | **-3.34 (-3.52 to -3.16)** | **5708 (0 to 28300)** | **2538 (0 to 12450)** | **39.47 (0 to 196.42)** | **14.02 (0 to 68.82)** | **-3.32 (-3.51 to -3.13)** |
| **North Macedonia** | **0.750955** | **High-middle SDI** | **33 (0 to 158)** | **36 (0 to 178)** | **1.83 (0 to 8.79)** | **1.17 (0 to 5.72)** | **-1.76 (-2.01 to -1.5)** | **841 (0 to 3988)** | **825 (0 to 4043)** | **43.73 (0 to 207.42)** | **24.91 (0 to 122.52)** | **-2.11 (-2.33 to -1.89)** |
| **Montenegro** | **0.796533** | **High-middle SDI** | **4 (0 to 22)** | **6 (0 to 31)** | **0.72 (0 to 3.51)** | **0.66 (0 to 3.28)** | **-0.43 (-0.62 to -0.24)** | **112 (0 to 542)** | **135 (0 to 680)** | **17.54 (0 to 84.88)** | **14.08 (0 to 70.68)** | **-0.84 (-1.08 to -0.61)** |
| **Poland** | **0.812073** | **NA** | **732 (0 to 3649)** | **507 (0 to 2513)** | **1.69 (0 to 8.4)** | **0.7 (0 to 3.46)** | **-2.99 (-3.12 to -2.87)** | **17532 (0 to 87992)** | **10990 (0 to 54421)** | **40.11 (0 to 201.26)** | **16.25 (0 to 80.58)** | **-2.99 (-3.13 to -2.86)** |
| **Romania** | **0.766321** | **High-middle SDI** | **348 (0 to 1795)** | **323 (0 to 1612)** | **1.26 (0 to 6.47)** | **0.87 (0 to 4.33)** | **-1.4 (-1.55 to -1.24)** | **9271 (0 to 47872)** | **7322 (0 to 36508)** | **32.75 (0 to 168.97)** | **21.42 (0 to 106.86)** | **-1.58 (-1.73 to -1.42)** |
| **Serbia** | **0.792213** | **High-middle SDI** | **118 (0 to 587)** | **109 (0 to 534)** | **1.16 (0 to 5.75)** | **0.65 (0 to 3.18)** | **-2.27 (-2.48 to -2.06)** | **3027 (0 to 15051)** | **2370 (0 to 11741)** | **27 (0 to 134.19)** | **15 (0 to 74.13)** | **-2.29 (-2.53 to -2.05)** |
| **Slovakia** | **0.808329** | **High-middle SDI** | **90 (0 to 439)** | **66 (0 to 330)** | **1.5 (0 to 7.32)** | **0.68 (0 to 3.44)** | **-2.49 (-2.59 to -2.39)** | **2178 (0 to 10668)** | **1451 (0 to 7270)** | **36.53 (0 to 178.7)** | **15.65 (0 to 78.59)** | **-2.66 (-2.76 to -2.56)** |
| **Slovenia** | **0.842633** | **High SDI** | **40 (0 to 196)** | **27 (0 to 132)** | **1.6 (0 to 7.88)** | **0.56 (0 to 2.81)** | **-3.5 (-3.61 to -3.39)** | **937 (0 to 4618)** | **508 (0 to 2525)** | **38.05 (0 to 187.63)** | **11.93 (0 to 59.18)** | **-3.88 (-4 to -3.76)** |
| **Eastern Europe** | **0.803414** | **NA** | **6323 (0 to 32996)** | **3241 (0 to 16645)** | **2.25 (0 to 11.72)** | **0.92 (0 to 4.74)** | **-3.11 (-3.21 to -3.01)** | **173733 (0 to 903204)** | **79240 (0 to 402533)** | **61.63 (0 to 320.72)** | **23.54 (0 to 119.38)** | **-3.4 (-3.52 to -3.28)** |
| **Belarus** | **0.784114** | **High-middle SDI** | **280 (0 to 1523)** | **134 (0 to 718)** | **2.16 (0 to 11.74)** | **0.84 (0 to 4.51)** | **-3.59 (-3.8 to -3.38)** | **7645 (0 to 41415)** | **3429 (0 to 18075)** | **59.39 (0 to 320.65)** | **22.55 (0 to 118.46)** | **-3.76 (-3.97 to -3.54)** |
| **Estonia** | **0.845787** | **High SDI** | **30 (0 to 170)** | **15 (0 to 87)** | **1.45 (0 to 8.32)** | **0.56 (0 to 3.14)** | **-3.29 (-3.41 to -3.18)** | **779 (0 to 4422)** | **325 (0 to 1819)** | **38.48 (0 to 218.65)** | **13.42 (0 to 74.98)** | **-3.66 (-3.79 to -3.53)** |
| **Latvia** | **0.830715** | **High SDI** | **62 (0 to 335)** | **30 (0 to 160)** | **1.73 (0 to 9.34)** | **0.78 (0 to 4.05)** | **-2.7 (-2.84 to -2.56)** | **1624 (0 to 8804)** | **682 (0 to 3521)** | **46.32 (0 to 250.83)** | **19.65 (0 to 100.56)** | **-2.98 (-3.14 to -2.82)** |
| **Lithuania** | **0.857613** | **High SDI** | **83 (0 to 435)** | **47 (0 to 241)** | **1.84 (0 to 9.65)** | **0.81 (0 to 4.17)** | **-2.58 (-2.7 to -2.46)** | **2129 (0 to 11207)** | **1027 (0 to 5281)** | **47.84 (0 to 251.53)** | **20.24 (0 to 104.25)** | **-2.73 (-2.87 to -2.6)** |
| **Republic of Moldova** | **0.732393** | **High-middle SDI** | **62 (0 to 335)** | **35 (0 to 188)** | **1.41 (0 to 7.6)** | **0.58 (0 to 3.15)** | **-2.32 (-2.67 to -1.97)** | **1771 (0 to 9555)** | **909 (0 to 4913)** | **38.48 (0 to 207.59)** | **15.63 (0 to 84.42)** | **-2.38 (-2.72 to -2.05)** |
| **Russian Federation** | **0.809111** | **NA** | **4499 (0 to 23008)** | **2439 (0 to 12348)** | **2.48 (0 to 12.65)** | **1.02 (0 to 5.15)** | **-3.05 (-3.16 to -2.94)** | **123581 (0 to 628901)** | **58560 (0 to 294977)** | **67.29 (0 to 342.55)** | **25.26 (0 to 127.12)** | **-3.39 (-3.51 to -3.26)** |
| **Ukraine** | **0.761046** | **NA** | **1306 (0 to 7142)** | **541 (0 to 3007)** | **1.83 (0 to 9.99)** | **0.71 (0 to 3.96)** | **-3.49 (-3.67 to -3.3)** | **36203 (0 to 197249)** | **14307 (0 to 79233)** | **51.57 (0 to 280.38)** | **19.95 (0 to 110.44)** | **-3.62 (-3.83 to -3.42)** |
| **High-income Asia Pacific** | **0.877157** | **NA** | **6059 (0 to 29937)** | **5864 (0 to 29532)** | **3.09 (0 to 15.26)** | **1.09 (0 to 5.43)** | **-3.44 (-3.48 to -3.39)** | **151938 (0 to 743670)** | **99017 (0 to 495285)** | **74.62 (0 to 365.66)** | **22.57 (0 to 112.13)** | **-3.92 (-3.97 to -3.87)** |
| **Brunei Darussalam** | **0.810289** | **High-middle SDI** | **2 (0 to 10)** | **3 (0 to 13)** | **1.95 (0 to 9.92)** | **0.84 (0 to 4.3)** | **-2.5 (-2.77 to -2.24)** | **58 (0 to 290)** | **77 (0 to 388)** | **46.76 (0 to 237.2)** | **19.53 (0 to 98.37)** | **-2.73 (-3.01 to -2.44)** |
| **Japan** | **0.87146** | **NA** | **4680 (0 to 23011)** | **4801 (0 to 24191)** | **2.81 (0 to 13.86)** | **1.09 (0 to 5.46)** | **-3.08 (-3.13 to -3.04)** | **110926 (0 to 544309)** | **76537 (0 to 383664)** | **65.73 (0 to 322.56)** | **22.26 (0 to 111.56)** | **-3.52 (-3.57 to -3.47)** |
| **Republic of Korea** | **0.887196** | **High SDI** | **1348 (0 to 6571)** | **1029 (0 to 5084)** | **4.61 (0 to 22.14)** | **1.11 (0 to 5.5)** | **-4.99 (-5.14 to -4.84)** | **40195 (0 to 196098)** | **21757 (0 to 106998)** | **118.73 (0 to 580.64)** | **24.08 (0 to 117.95)** | **-5.43 (-5.55 to -5.3)** |
| **Singapore** | **0.856235** | **High SDI** | **29 (0 to 141)** | **31 (0 to 155)** | **1.37 (0 to 6.71)** | **0.38 (0 to 1.87)** | **-4.27 (-4.51 to -4.03)** | **760 (0 to 3684)** | **646 (0 to 3207)** | **32.17 (0 to 157.39)** | **7.62 (0 to 37.78)** | **-4.65 (-4.89 to -4.4)** |
| **Australasia** | **0.845644** | **NA** | **113 (0 to 630)** | **138 (0 to 788)** | **0.49 (0 to 2.7)** | **0.25 (0 to 1.41)** | **-2.1 (-2.22 to -1.99)** | **2603 (0 to 14210)** | **2809 (0 to 15483)** | **11.25 (0 to 61.37)** | **5.72 (0 to 31.14)** | **-2.16 (-2.26 to -2.05)** |
| **Australia** | **0.844269** | **High SDI** | **86 (0 to 482)** | **109 (0 to 629)** | **0.44 (0 to 2.49)** | **0.23 (0 to 1.34)** | **-2.01 (-2.1 to -1.92)** | **1976 (0 to 10969)** | **2198 (0 to 12383)** | **10.26 (0 to 56.75)** | **5.32 (0 to 29.71)** | **-2.07 (-2.15 to -1.99)** |
| **New Zealand** | **0.850145** | **NA** | **27 (0 to 143)** | **29 (0 to 157)** | **0.7 (0 to 3.65)** | **0.34 (0 to 1.84)** | **-2.37 (-2.63 to -2.11)** | **627 (0 to 3278)** | **611 (0 to 3275)** | **16.26 (0 to 84.84)** | **7.79 (0 to 41.76)** | **-2.45 (-2.69 to -2.2)** |
| **Western Europe** | **0.848729** | **NA** | **6132 (0 to 32392)** | **4050 (0 to 21331)** | **1.04 (0 to 5.47)** | **0.41 (0 to 2.1)** | **-3 (-3.1 to -2.9)** | **127536 (0 to 667811)** | **76171 (0 to 392855)** | **22.85 (0 to 119.24)** | **8.93 (0 to 45.98)** | **-2.96 (-3.05 to -2.88)** |
| **Andorra** | **0.869895** | **High SDI** | **0 (0 to 3)** | **1 (0 to 4)** | **0.89 (0 to 4.96)** | **0.46 (0 to 2.66)** | **-1.9 (-2.15 to -1.65)** | **12 (0 to 63)** | **15 (0 to 86)** | **19.76 (0 to 108.15)** | **9.75 (0 to 56.37)** | **-2.03 (-2.26 to -1.79)** |
| **Austria** | **0.854558** | **High SDI** | **161 (0 to 812)** | **72 (0 to 364)** | **1.31 (0 to 6.6)** | **0.37 (0 to 1.88)** | **-3.99 (-4.15 to -3.82)** | **3199 (0 to 16156)** | **1368 (0 to 6945)** | **27.72 (0 to 140.14)** | **8.04 (0 to 40.82)** | **-3.86 (-4.04 to -3.68)** |
| **Belgium** | **0.853674** | **High SDI** | **153 (0 to 773)** | **91 (0 to 460)** | **0.96 (0 to 4.87)** | **0.36 (0 to 1.8)** | **-3.08 (-3.27 to -2.88)** | **2947 (0 to 14811)** | **1649 (0 to 8401)** | **19.59 (0 to 98.23)** | **7.56 (0 to 38.48)** | **-2.95 (-3.1 to -2.81)** |
| **Cyprus** | **0.835649** | **High SDI** | **6 (0 to 30)** | **9 (0 to 45)** | **0.89 (0 to 4.54)** | **0.44 (0 to 2.31)** | **-1.88 (-2.09 to -1.68)** | **127 (0 to 665)** | **169 (0 to 889)** | **16.99 (0 to 88.57)** | **8.45 (0 to 44.6)** | **-1.88 (-2.02 to -1.75)** |
| **Denmark** | **0.897314** | **High SDI** | **57 (0 to 294)** | **43 (0 to 226)** | **0.69 (0 to 3.57)** | **0.35 (0 to 1.83)** | **-2.05 (-2.34 to -1.77)** | **1209 (0 to 6296)** | **851 (0 to 4473)** | **15.96 (0 to 82.73)** | **7.71 (0 to 40.23)** | **-2.21 (-2.48 to -1.95)** |
| **Finland** | **0.860244** | **High SDI** | **69 (0 to 365)** | **42 (0 to 218)** | **0.96 (0 to 5.05)** | **0.31 (0 to 1.6)** | **-3.58 (-3.74 to -3.41)** | **1526 (0 to 8006)** | **766 (0 to 3996)** | **21.97 (0 to 114.77)** | **6.68 (0 to 34.87)** | **-3.69 (-3.81 to -3.57)** |
| **France** | **0.837816** | **High SDI** | **668 (0 to 3542)** | **503 (0 to 2641)** | **0.77 (0 to 4.1)** | **0.33 (0 to 1.73)** | **-2.53 (-2.64 to -2.43)** | **12979 (0 to 68947)** | **9345 (0 to 49117)** | **16.14 (0 to 85.72)** | **7.47 (0 to 39.32)** | **-2.19 (-2.29 to -2.1)** |
| **Germany** | **0.903516** | **High SDI** | **1400 (0 to 7439)** | **882 (0 to 4466)** | **1.07 (0 to 5.68)** | **0.44 (0 to 2.2)** | **-2.99 (-3.16 to -2.83)** | **29254 (0 to 153643)** | **17257 (0 to 86397)** | **23.9 (0 to 125.09)** | **10.09 (0 to 50.47)** | **-2.83 (-2.98 to -2.68)** |
| **Greece** | **0.791882** | **High-middle SDI** | **183 (0 to 960)** | **144 (0 to 752)** | **1.22 (0 to 6.37)** | **0.55 (0 to 2.87)** | **-2.87 (-3.1 to -2.63)** | **3857 (0 to 20094)** | **2624 (0 to 13614)** | **26.03 (0 to 135.49)** | **12.22 (0 to 62.73)** | **-2.67 (-2.88 to -2.46)** |
| **Iceland** | **0.874629** | **High SDI** | **3 (0 to 15)** | **2 (0 to 11)** | **1.04 (0 to 5.28)** | **0.33 (0 to 1.74)** | **-3.53 (-3.62 to -3.44)** | **64 (0 to 328)** | **38 (0 to 202)** | **22.95 (0 to 117.69)** | **6.92 (0 to 36.08)** | **-3.76 (-3.84 to -3.68)** |
| **Ireland** | **0.87399** | **High SDI** | **37 (0 to 200)** | **25 (0 to 139)** | **0.91 (0 to 4.87)** | **0.31 (0 to 1.69)** | **-3.37 (-3.45 to -3.29)** | **798 (0 to 4275)** | **503 (0 to 2717)** | **19.82 (0 to 105.93)** | **6.53 (0 to 35.12)** | **-3.56 (-3.66 to -3.46)** |
| **Israel** | **0.809091** | **High-middle SDI** | **39 (0 to 202)** | **49 (0 to 256)** | **0.82 (0 to 4.27)** | **0.39 (0 to 1.99)** | **-2.81 (-2.96 to -2.65)** | **825 (0 to 4310)** | **978 (0 to 5020)** | **17.35 (0 to 90.49)** | **8.23 (0 to 42.16)** | **-2.75 (-2.91 to -2.59)** |
| **Italy** | **0.805537** | **NA** | **1369 (0 to 6948)** | **906 (0 to 4752)** | **1.53 (0 to 7.75)** | **0.56 (0 to 2.9)** | **-3.2 (-3.27 to -3.13)** | **28833 (0 to 144128)** | **16109 (0 to 82405)** | **33.51 (0 to 166.96)** | **11.95 (0 to 60.43)** | **-3.27 (-3.33 to -3.21)** |
| **Luxembourg** | **0.884636** | **High SDI** | **5 (0 to 28)** | **4 (0 to 20)** | **0.99 (0 to 5.15)** | **0.33 (0 to 1.74)** | **-3.41 (-3.51 to -3.31)** | **117 (0 to 606)** | **70 (0 to 366)** | **22.11 (0 to 114.17)** | **6.68 (0 to 34.69)** | **-3.74 (-3.82 to -3.67)** |
| **Malta** | **0.801854** | **High-middle SDI** | **4 (0 to 22)** | **4 (0 to 19)** | **1.04 (0 to 5.3)** | **0.37 (0 to 1.87)** | **-3.18 (-3.33 to -3.03)** | **96 (0 to 490)** | **72 (0 to 366)** | **22.49 (0 to 114.32)** | **7.88 (0 to 40.12)** | **-3.2 (-3.33 to -3.07)** |
| **Netherlands** | **0.888376** | **High SDI** | **181 (0 to 976)** | **126 (0 to 673)** | **0.89 (0 to 4.78)** | **0.34 (0 to 1.81)** | **-3.12 (-3.23 to -3.01)** | **3776 (0 to 20125)** | **2356 (0 to 12388)** | **19.33 (0 to 102.6)** | **7.05 (0 to 36.75)** | **-3.22 (-3.35 to -3.1)** |
| **Norway** | **0.916632** | **NA** | **55 (0 to 292)** | **28 (0 to 151)** | **0.77 (0 to 4.09)** | **0.26 (0 to 1.41)** | **-3.49 (-3.55 to -3.43)** | **1103 (0 to 5818)** | **525 (0 to 2791)** | **17.2 (0 to 90.45)** | **5.45 (0 to 28.98)** | **-3.7 (-3.76 to -3.64)** |
| **Portugal** | **0.745395** | **High-middle SDI** | **265 (0 to 1461)** | **197 (0 to 1062)** | **1.97 (0 to 10.86)** | **0.78 (0 to 4.15)** | **-2.88 (-2.93 to -2.84)** | **6253 (0 to 33606)** | **3960 (0 to 20797)** | **47.01 (0 to 252.12)** | **18.46 (0 to 94.95)** | **-2.93 (-3.03 to -2.83)** |
| **Spain** | **0.769483** | **High-middle SDI** | **500 (0 to 2919)** | **354 (0 to 2055)** | **0.93 (0 to 5.37)** | **0.36 (0 to 2.03)** | **-2.88 (-3.01 to -2.75)** | **11553 (0 to 65693)** | **7484 (0 to 41726)** | **22.46 (0 to 125.38)** | **8.78 (0 to 47.77)** | **-2.87 (-2.98 to -2.76)** |
| **Sweden** | **0.887384** | **NA** | **111 (0 to 587)** | **60 (0 to 314)** | **0.7 (0 to 3.69)** | **0.25 (0 to 1.32)** | **-3.44 (-3.64 to -3.25)** | **2168 (0 to 11258)** | **1055 (0 to 5509)** | **15.21 (0 to 78.16)** | **5.16 (0 to 26.96)** | **-3.49 (-3.65 to -3.33)** |
| **Switzerland** | **0.933532** | **High SDI** | **85 (0 to 451)** | **55 (0 to 291)** | **0.79 (0 to 4.16)** | **0.28 (0 to 1.49)** | **-3 (-3.35 to -2.65)** | **1766 (0 to 9242)** | **1034 (0 to 5450)** | **17.73 (0 to 92.24)** | **6.11 (0 to 31.85)** | **-3.11 (-3.44 to -2.78)** |
| **United Kingdom** | **0.858445** | **NA** | **775 (0 to 4186)** | **449 (0 to 2435)** | **0.81 (0 to 4.4)** | **0.32 (0 to 1.71)** | **-3.04 (-3.15 to -2.92)** | **14943 (0 to 81249)** | **7853 (0 to 42733)** | **16.6 (0 to 90.58)** | **6.22 (0 to 33.88)** | **-3.13 (-3.26 to -3)** |
| **Southern Latin America** | **0.74303** | **NA** | **685 (0 to 3455)** | **752 (0 to 3767)** | **1.52 (0 to 7.61)** | **0.85 (0 to 4.26)** | **-1.69 (-1.8 to -1.58)** | **16278 (0 to 82265)** | **16599 (0 to 82996)** | **35 (0 to 176.91)** | **19.43 (0 to 97.04)** | **-1.73 (-1.84 to -1.62)** |
| **Argentina** | **0.733528** | **High-middle SDI** | **363 (0 to 1854)** | **382 (0 to 1940)** | **1.15 (0 to 5.84)** | **0.68 (0 to 3.43)** | **-1.46 (-1.6 to -1.31)** | **8635 (0 to 44566)** | **8672 (0 to 43936)** | **26.69 (0 to 137.72)** | **15.85 (0 to 80.22)** | **-1.42 (-1.58 to -1.27)** |
| **Chile** | **0.770149** | **High-middle SDI** | **270 (0 to 1353)** | **319 (0 to 1605)** | **2.79 (0 to 13.97)** | **1.23 (0 to 6.18)** | **-2.54 (-2.65 to -2.43)** | **6472 (0 to 32469)** | **6894 (0 to 34597)** | **63.24 (0 to 317.68)** | **27.13 (0 to 135.98)** | **-2.63 (-2.72 to -2.55)** |
| **Uruguay** | **0.721713** | **High-middle SDI** | **53 (0 to 264)** | **50 (0 to 253)** | **1.35 (0 to 6.75)** | **0.87 (0 to 4.42)** | **-1.53 (-1.64 to -1.42)** | **1170 (0 to 5920)** | **1032 (0 to 5234)** | **30.8 (0 to 155.66)** | **20.11 (0 to 102.22)** | **-1.48 (-1.57 to -1.39)** |
| **High-income North America** | **0.864217** | **NA** | **1414 (0 to 7466)** | **1482 (0 to 7675)** | **0.4 (0 to 2.1)** | **0.23 (0 to 1.17)** | **-1.88 (-1.92 to -1.83)** | **31635 (0 to 165388)** | **32631 (0 to 166471)** | **9.34 (0 to 48.84)** | **5.52 (0 to 28.1)** | **-1.73 (-1.77 to -1.68)** |
| **Canada** | **0.873182** | **High SDI** | **196 (0 to 1015)** | **226 (0 to 1168)** | **0.6 (0 to 3.11)** | **0.3 (0 to 1.56)** | **-2.05 (-2.17 to -1.94)** | **4334 (0 to 22115)** | **4428 (0 to 22545)** | **13.45 (0 to 68.65)** | **6.63 (0 to 33.55)** | **-2.13 (-2.23 to -2.03)** |
| **United States of America** | **0.863244** | **NA** | **1217 (0 to 6444)** | **1255 (0 to 6523)** | **0.38 (0 to 2)** | **0.22 (0 to 1.13)** | **-1.89 (-1.95 to -1.83)** | **27284 (0 to 143482)** | **28190 (0 to 144065)** | **8.92 (0 to 46.83)** | **5.38 (0 to 27.4)** | **-1.69 (-1.74 to -1.63)** |
| **Caribbean** | **0.642315** | **NA** | **236 (0 to 1223)** | **311 (0 to 1671)** | **0.94 (0 to 4.83)** | **0.58 (0 to 3.1)** | **-1.48 (-1.55 to -1.4)** | **5805 (0 to 30338)** | **7608 (0 to 40990)** | **21.93 (0 to 114.63)** | **14.24 (0 to 76.67)** | **-1.31 (-1.41 to -1.21)** |
| **Antigua and Barbuda** | **0.74985** | **High-middle SDI** | **1 (0 to 4)** | **1 (0 to 4)** | **1.33 (0 to 6.81)** | **0.76 (0 to 4.01)** | **-1.8 (-1.96 to -1.63)** | **16 (0 to 81)** | **17 (0 to 93)** | **30.31 (0 to 154.49)** | **16.16 (0 to 85.68)** | **-2 (-2.16 to -1.84)** |
| **Bahamas** | **0.805144** | **High-middle SDI** | **2 (0 to 8)** | **3 (0 to 14)** | **1.04 (0 to 5.44)** | **0.66 (0 to 3.61)** | **-1.44 (-1.57 to -1.3)** | **43 (0 to 231)** | **67 (0 to 365)** | **25.98 (0 to 138.09)** | **15.77 (0 to 86.67)** | **-1.59 (-1.72 to -1.46)** |
| **Barbados** | **0.747065** | **High-middle SDI** | **4 (0 to 19)** | **3 (0 to 19)** | **1.18 (0 to 6.36)** | **0.66 (0 to 3.73)** | **-1.89 (-2.1 to -1.68)** | **75 (0 to 402)** | **72 (0 to 410)** | **26.59 (0 to 142.59)** | **14.46 (0 to 82.29)** | **-1.93 (-2.1 to -1.76)** |
| **Belize** | **0.610552** | **Low-middle SDI** | **1 (0 to 4)** | **2 (0 to 10)** | **0.93 (0 to 4.82)** | **0.7 (0 to 3.66)** | **-1.11 (-1.67 to -0.54)** | **21 (0 to 108)** | **53 (0 to 276)** | **21.56 (0 to 112.75)** | **16.51 (0 to 86.39)** | **-1.04 (-1.59 to -0.48)** |
| **Cuba** | **0.669332** | **Middle SDI** | **61 (0 to 317)** | **83 (0 to 429)** | **0.6 (0 to 3.14)** | **0.42 (0 to 2.15)** | **-1.02 (-1.13 to -0.91)** | **1386 (0 to 7286)** | **1805 (0 to 9313)** | **13.48 (0 to 70.86)** | **9.47 (0 to 48.88)** | **-1.01 (-1.11 to -0.9)** |
| **Dominica** | **0.747382** | **High-middle SDI** | **1 (0 to 7)** | **1 (0 to 6)** | **2.21 (0 to 11.61)** | **1.51 (0 to 7.98)** | **-1.32 (-1.44 to -1.2)** | **28 (0 to 149)** | **28 (0 to 147)** | **48.02 (0 to 252.12)** | **33.29 (0 to 175.28)** | **-1.22 (-1.36 to -1.08)** |
| **Dominican Republic** | **0.619171** | **Middle SDI** | **22 (0 to 115)** | **51 (0 to 273)** | **0.64 (0 to 3.38)** | **0.52 (0 to 2.76)** | **-0.28 (-0.44 to -0.12)** | **584 (0 to 3102)** | **1278 (0 to 6776)** | **14.82 (0 to 78.25)** | **12.45 (0 to 66.1)** | **-0.17 (-0.29 to -0.04)** |
| **Grenada** | **0.669351** | **Middle SDI** | **1 (0 to 4)** | **1 (0 to 4)** | **1.1 (0 to 5.69)** | **0.64 (0 to 3.35)** | **-1.56 (-1.88 to -1.24)** | **18 (0 to 94)** | **17 (0 to 88)** | **26.44 (0 to 137.7)** | **14.34 (0 to 75.83)** | **-1.74 (-1.96 to -1.52)** |
| **Guyana** | **0.650902** | **Middle SDI** | **4 (0 to 20)** | **4 (0 to 20)** | **1.06 (0 to 5.47)** | **0.59 (0 to 3.26)** | **-1.65 (-1.92 to -1.39)** | **102 (0 to 531)** | **100 (0 to 561)** | **25.37 (0 to 132.5)** | **14.74 (0 to 82.09)** | **-1.5 (-1.76 to -1.23)** |
| **Haiti** | **0.448751** | **Low SDI** | **65 (0 to 345)** | **91 (0 to 487)** | **2.16 (0 to 11.4)** | **1.38 (0 to 7.33)** | **-1.32 (-1.41 to -1.24)** | **1833 (0 to 9806)** | **2590 (0 to 13906)** | **52.78 (0 to 283.13)** | **32.74 (0 to 174.87)** | **-1.4 (-1.5 to -1.3)** |
| **Jamaica** | **0.683064** | **Middle SDI** | **22 (0 to 112)** | **22 (0 to 117)** | **1.19 (0 to 6.1)** | **0.69 (0 to 3.74)** | **-1.84 (-2.15 to -1.53)** | **461 (0 to 2376)** | **486 (0 to 2648)** | **26.02 (0 to 134.3)** | **15.73 (0 to 85.8)** | **-1.74 (-2.08 to -1.4)** |
| **Saint Lucia** | **0.672602** | **Middle SDI** | **1 (0 to 7)** | **2 (0 to 11)** | **1.69 (0 to 8.8)** | **0.82 (0 to 4.55)** | **-2.8 (-3.09 to -2.51)** | **33 (0 to 171)** | **44 (0 to 244)** | **37.52 (0 to 197.51)** | **18.38 (0 to 102.01)** | **-2.64 (-2.88 to -2.41)** |
| **Saint Vincent and the Grenadines** | **0.640887** | **Middle SDI** | **1 (0 to 5)** | **1 (0 to 6)** | **1.37 (0 to 7.07)** | **0.79 (0 to 4.21)** | **-1.76 (-1.99 to -1.53)** | **22 (0 to 115)** | **26 (0 to 137)** | **31.36 (0 to 161.79)** | **18.1 (0 to 96.88)** | **-1.77 (-2 to -1.55)** |
| **Suriname** | **0.641163** | **Middle SDI** | **2 (0 to 11)** | **3 (0 to 18)** | **0.85 (0 to 4.5)** | **0.52 (0 to 2.87)** | **-1.45 (-1.66 to -1.23)** | **54 (0 to 289)** | **82 (0 to 445)** | **20.27 (0 to 108.85)** | **12.64 (0 to 69.06)** | **-1.45 (-1.68 to -1.23)** |
| **Trinidad and Tobago** | **0.769401** | **High-middle SDI** | **7 (0 to 36)** | **7 (0 to 38)** | **0.87 (0 to 4.53)** | **0.39 (0 to 2.02)** | **-2.78 (-2.99 to -2.57)** | **167 (0 to 883)** | **180 (0 to 940)** | **19.65 (0 to 103.45)** | **9.44 (0 to 49.15)** | **-2.57 (-2.79 to -2.35)** |
| **Andean Latin America** | **0.654008** | **NA** | **522 (0 to 2645)** | **988 (0 to 4999)** | **2.67 (0 to 13.48)** | **1.71 (0 to 8.65)** | **-1.64 (-1.79 to -1.5)** | **13410 (0 to 68084)** | **23128 (0 to 116684)** | **62.37 (0 to 316.56)** | **38.45 (0 to 193.86)** | **-1.79 (-1.93 to -1.64)** |
| **Bolivia (Plurinational State of)** | **0.604497** | **Low-middle SDI** | **125 (0 to 631)** | **219 (0 to 1113)** | **4.17 (0 to 21.2)** | **2.58 (0 to 13.23)** | **-1.56 (-1.62 to -1.5)** | **3286 (0 to 16547)** | **5259 (0 to 26843)** | **97.64 (0 to 491.71)** | **56.47 (0 to 288.2)** | **-1.85 (-1.93 to -1.77)** |
| **Ecuador** | **0.665675** | **Middle SDI** | **128 (0 to 637)** | **238 (0 to 1217)** | **2.55 (0 to 12.69)** | **1.5 (0 to 7.64)** | **-1.81 (-2.22 to -1.4)** | **3202 (0 to 15959)** | **5576 (0 to 28478)** | **57.91 (0 to 289.9)** | **33.62 (0 to 171.72)** | **-1.88 (-2.26 to -1.5)** |
| **Peru** | **0.662036** | **Middle SDI** | **269 (0 to 1383)** | **531 (0 to 2774)** | **2.33 (0 to 12.03)** | **1.6 (0 to 8.33)** | **-1.56 (-1.83 to -1.3)** | **6923 (0 to 35443)** | **12293 (0 to 63938)** | **54.88 (0 to 280.51)** | **36.04 (0 to 187.32)** | **-1.7 (-1.97 to -1.44)** |
| **Central Latin America** | **0.641931** | **NA** | **1316 (0 to 6625)** | **2295 (0 to 11895)** | **1.7 (0 to 8.53)** | **0.93 (0 to 4.83)** | **-2.18 (-2.26 to -2.1)** | **33796 (0 to 170528)** | **57297 (0 to 297890)** | **38.56 (0 to 194.24)** | **22.41 (0 to 116.52)** | **-2 (-2.09 to -1.92)** |
| **Colombia** | **0.65664** | **Middle SDI** | **432 (0 to 2146)** | **616 (0 to 3149)** | **2.61 (0 to 12.88)** | **1.12 (0 to 5.7)** | **-3.13 (-3.27 to -3)** | **11274 (0 to 56725)** | **14940 (0 to 76563)** | **60.69 (0 to 303.31)** | **27.16 (0 to 139.11)** | **-2.92 (-3.07 to -2.77)** |
| **Costa Rica** | **0.70437** | **Middle SDI** | **50 (0 to 250)** | **75 (0 to 379)** | **2.97 (0 to 14.7)** | **1.36 (0 to 6.87)** | **-2.83 (-3.04 to -2.62)** | **1218 (0 to 6028)** | **1766 (0 to 8912)** | **67.48 (0 to 334.34)** | **32.07 (0 to 161.83)** | **-2.78 (-3 to -2.55)** |
| **El Salvador** | **0.56557** | **Low-middle SDI** | **47 (0 to 238)** | **90 (0 to 457)** | **1.61 (0 to 8.13)** | **1.42 (0 to 7.23)** | **-0.77 (-1 to -0.54)** | **1238 (0 to 6245)** | **2135 (0 to 10919)** | **39.75 (0 to 200.31)** | **34.85 (0 to 178.14)** | **-0.79 (-1.02 to -0.57)** |
| **Guatemala** | **0.540099** | **Low-middle SDI** | **78 (0 to 389)** | **210 (0 to 1087)** | **2.65 (0 to 13.21)** | **1.98 (0 to 10.25)** | **-1.3 (-1.95 to -0.64)** | **2174 (0 to 10801)** | **5396 (0 to 27893)** | **58.91 (0 to 293.38)** | **46.77 (0 to 242.03)** | **-1.15 (-1.77 to -0.53)** |
| **Honduras** | **0.513586** | **Low-middle SDI** | **28 (0 to 139)** | **98 (0 to 498)** | **1.43 (0 to 7.09)** | **1.68 (0 to 8.55)** | **0.69 (0.49 to 0.89)** | **797 (0 to 3964)** | **2436 (0 to 12396)** | **35.34 (0 to 176.07)** | **37.28 (0 to 189.21)** | **0.32 (0.17 to 0.47)** |
| **Mexico** | **0.664969** | **NA** | **496 (0 to 2518)** | **877 (0 to 4573)** | **1.29 (0 to 6.54)** | **0.71 (0 to 3.7)** | **-2.08 (-2.22 to -1.94)** | **12328 (0 to 63326)** | **22346 (0 to 116943)** | **27.82 (0 to 142.03)** | **17.14 (0 to 89.36)** | **-1.74 (-1.87 to -1.6)** |
| **Nicaragua** | **0.523647** | **Low-middle SDI** | **17 (0 to 85)** | **37 (0 to 189)** | **1.14 (0 to 5.74)** | **0.79 (0 to 3.99)** | **-1.1 (-1.37 to -0.83)** | **458 (0 to 2335)** | **979 (0 to 5000)** | **27.27 (0 to 138.44)** | **18.95 (0 to 96.48)** | **-1.13 (-1.36 to -0.89)** |
| **Panama** | **0.70666** | **Middle SDI** | **17 (0 to 84)** | **36 (0 to 192)** | **1.17 (0 to 5.86)** | **0.81 (0 to 4.31)** | **-1.14 (-1.29 to -0.99)** | **410 (0 to 2045)** | **833 (0 to 4405)** | **26.59 (0 to 132.63)** | **18.87 (0 to 99.69)** | **-1.13 (-1.28 to -0.98)** |
| **Venezuela (Bolivarian Republic of)** | **0.5966** | **Low-middle SDI** | **151 (0 to 756)** | **256 (0 to 1314)** | **1.64 (0 to 8.26)** | **0.88 (0 to 4.5)** | **-2.46 (-2.65 to -2.26)** | **3898 (0 to 19406)** | **6465 (0 to 33104)** | **38.29 (0 to 191.2)** | **21.23 (0 to 108.69)** | **-2.35 (-2.55 to -2.16)** |
| **Tropical Latin America** | **0.648942** | **NA** | **1356 (0 to 6802)** | **1988 (0 to 10206)** | **1.58 (0 to 7.95)** | **0.78 (0 to 4.01)** | **-2.36 (-2.42 to -2.31)** | **35940 (0 to 179652)** | **49223 (0 to 252502)** | **37.33 (0 to 186.56)** | **18.88 (0 to 96.83)** | **-2.33 (-2.39 to -2.27)** |
| **Brazil** | **0.648847** | **NA** | **1338 (0 to 6704)** | **1948 (0 to 10007)** | **1.6 (0 to 8.04)** | **0.78 (0 to 4.02)** | **-2.39 (-2.45 to -2.34)** | **35485 (0 to 177283)** | **48260 (0 to 247639)** | **37.77 (0 to 188.6)** | **18.95 (0 to 97.31)** | **-2.35 (-2.41 to -2.3)** |
| **Paraguay** | **0.650488** | **Middle SDI** | **18 (0 to 93)** | **40 (0 to 205)** | **0.85 (0 to 4.33)** | **0.7 (0 to 3.63)** | **-0.85 (-1.08 to -0.63)** | **454 (0 to 2321)** | **963 (0 to 5025)** | **19.72 (0 to 100.22)** | **16.13 (0 to 83.9)** | **-0.94 (-1.17 to -0.72)** |
| **North Africa and Middle East** | **0.658716** | **NA** | **1257 (0 to 7341)** | **1977 (0 to 11966)** | **0.75 (0 to 4.4)** | **0.45 (0 to 2.75)** | **-1.58 (-1.64 to -1.51)** | **37024 (0 to 214621)** | **54759 (0 to 325676)** | **19.66 (0 to 114.77)** | **11 (0 to 65.92)** | **-1.83 (-1.9 to -1.77)** |
| **North Africa and Middle East** | **0.658716** | **NA** | **1257 (0 to 7341)** | **1977 (0 to 11966)** | **0.75 (0 to 4.4)** | **0.45 (0 to 2.75)** | **-1.58 (-1.64 to -1.51)** | **37024 (0 to 214621)** | **54759 (0 to 325676)** | **19.66 (0 to 114.77)** | **11 (0 to 65.92)** | **-1.83 (-1.9 to -1.77)** |
| **Algeria** | **0.65972** | **Middle SDI** | **36 (0 to 212)** | **60 (0 to 361)** | **0.32 (0 to 1.92)** | **0.18 (0 to 1.09)** | **-1.67 (-1.8 to -1.54)** | **1004 (0 to 5942)** | **1596 (0 to 9529)** | **7.63 (0 to 45.49)** | **4.22 (0 to 25.39)** | **-1.87 (-1.97 to -1.78)** |
| **Bahrain** | **0.752218** | **High-middle SDI** | **1 (0 to 7)** | **2 (0 to 14)** | **0.73 (0 to 4.32)** | **0.33 (0 to 1.99)** | **-3.11 (-3.46 to -2.77)** | **32 (0 to 192)** | **69 (0 to 410)** | **16.49 (0 to 96.66)** | **6.92 (0 to 42.21)** | **-3.38 (-3.69 to -3.07)** |
| **Egypt** | **0.603962** | **Low-middle SDI** | **67 (0 to 377)** | **216 (0 to 1297)** | **0.24 (0 to 1.4)** | **0.36 (0 to 2.16)** | **2.49 (1.88 to 3.1)** | **2127 (0 to 12030)** | **6315 (0 to 37759)** | **6.57 (0 to 37.37)** | **8.87 (0 to 53.2)** | **2.07 (1.52 to 2.63)** |
| **Iran (Islamic Republic of)** | **0.697293** | **NA** | **307 (0 to 1755)** | **492 (0 to 2964)** | **1.23 (0 to 7.2)** | **0.66 (0 to 4.04)** | **-1.77 (-1.91 to -1.63)** | **8782 (0 to 50205)** | **12704 (0 to 74973)** | **30.43 (0 to 175.31)** | **15.55 (0 to 92.33)** | **-1.95 (-2.1 to -1.8)** |
| **Iraq** | **0.662777** | **Middle SDI** | **29 (0 to 168)** | **62 (0 to 375)** | **0.35 (0 to 2.05)** | **0.26 (0 to 1.59)** | **-1.25 (-1.37 to -1.13)** | **879 (0 to 5160)** | **1799 (0 to 10741)** | **9.89 (0 to 58.08)** | **6.62 (0 to 39.91)** | **-1.56 (-1.64 to -1.47)** |
| **Jordan** | **0.72542** | **High-middle SDI** | **5 (0 to 29)** | **14 (0 to 82)** | **0.38 (0 to 2.2)** | **0.19 (0 to 1.17)** | **-2.31 (-2.5 to -2.11)** | **155 (0 to 877)** | **397 (0 to 2291)** | **9.9 (0 to 55.99)** | **4.69 (0 to 27.71)** | **-2.62 (-2.83 to -2.4)** |
| **Kuwait** | **0.846802** | **High SDI** | **2 (0 to 9)** | **5 (0 to 26)** | **0.29 (0 to 1.56)** | **0.18 (0 to 1)** | **-1.66 (-2.1 to -1.23)** | **52 (0 to 276)** | **134 (0 to 709)** | **6.99 (0 to 37.37)** | **3.98 (0 to 21.48)** | **-2.04 (-2.47 to -1.6)** |
| **Lebanon** | **0.741226** | **High-middle SDI** | **11 (0 to 68)** | **16 (0 to 98)** | **0.54 (0 to 3.23)** | **0.27 (0 to 1.59)** | **-1.95 (-2.12 to -1.79)** | **314 (0 to 1845)** | **371 (0 to 2188)** | **13.7 (0 to 81.02)** | **6.22 (0 to 36.59)** | **-2.25 (-2.41 to -2.1)** |
| **Libya** | **0.735084** | **High-middle SDI** | **8 (0 to 47)** | **17 (0 to 107)** | **0.41 (0 to 2.55)** | **0.33 (0 to 2.06)** | **-0.37 (-0.54 to -0.2)** | **219 (0 to 1323)** | **510 (0 to 3156)** | **10.54 (0 to 63.86)** | **8.46 (0 to 52.16)** | **-0.45 (-0.6 to -0.29)** |
| **Morocco** | **0.56168** | **Low-middle SDI** | **24 (0 to 138)** | **43 (0 to 263)** | **0.17 (0 to 0.98)** | **0.13 (0 to 0.78)** | **-0.83 (-0.91 to -0.75)** | **675 (0 to 3875)** | **1135 (0 to 6797)** | **4.4 (0 to 25.34)** | **3.14 (0 to 18.98)** | **-1.02 (-1.08 to -0.96)** |
| **Palestine** | **0.629202** | **Middle SDI** | **5 (0 to 32)** | **7 (0 to 44)** | **0.61 (0 to 3.82)** | **0.31 (0 to 1.87)** | **-2.23 (-2.47 to -1.98)** | **132 (0 to 806)** | **205 (0 to 1204)** | **14.49 (0 to 89.29)** | **7.37 (0 to 43.42)** | **-2.26 (-2.47 to -2.05)** |
| **Oman** | **0.773801** | **High-middle SDI** | **4 (0 to 24)** | **5 (0 to 31)** | **0.61 (0 to 3.55)** | **0.28 (0 to 1.66)** | **-2.15 (-2.29 to -2.01)** | **125 (0 to 715)** | **155 (0 to 884)** | **15.64 (0 to 91.83)** | **6.41 (0 to 37.75)** | **-2.55 (-2.69 to -2.41)** |
| **Qatar** | **0.846704** | **High SDI** | **1 (0 to 4)** | **2 (0 to 13)** | **0.85 (0 to 4.92)** | **0.29 (0 to 1.75)** | **-3.69 (-4.32 to -3.06)** | **25 (0 to 145)** | **78 (0 to 436)** | **18.82 (0 to 107.01)** | **6.17 (0 to 36.75)** | **-3.75 (-4.32 to -3.19)** |
| **Saudi Arabia** | **0.814516** | **High SDI** | **20 (0 to 121)** | **37 (0 to 212)** | **0.35 (0 to 2.11)** | **0.19 (0 to 1.11)** | **-2.1 (-2.28 to -1.91)** | **591 (0 to 3527)** | **1261 (0 to 7213)** | **8.68 (0 to 52.7)** | **4.7 (0 to 27.44)** | **-2.02 (-2.19 to -1.85)** |
| **Syrian Arab Republic** | **0.622856** | **Middle SDI** | **22 (0 to 130)** | **37 (0 to 221)** | **0.42 (0 to 2.55)** | **0.31 (0 to 1.81)** | **-1.27 (-1.41 to -1.13)** | **646 (0 to 3784)** | **1000 (0 to 5849)** | **10.94 (0 to 65.25)** | **7.23 (0 to 42.5)** | **-1.57 (-1.74 to -1.4)** |
| **Tunisia** | **0.681701** | **Middle SDI** | **17 (0 to 99)** | **28 (0 to 168)** | **0.34 (0 to 2.03)** | **0.21 (0 to 1.28)** | **-1.7 (-1.78 to -1.61)** | **451 (0 to 2606)** | **726 (0 to 4396)** | **8.39 (0 to 49.2)** | **5.29 (0 to 31.84)** | **-1.68 (-1.76 to -1.59)** |
| **Turkey** | **0.713246** | **High-middle SDI** | **385 (0 to 2389)** | **470 (0 to 2935)** | **1.11 (0 to 6.89)** | **0.51 (0 to 3.18)** | **-2.65 (-2.99 to -2.31)** | **11559 (0 to 69945)** | **12107 (0 to 73875)** | **29.87 (0 to 183.06)** | **12.53 (0 to 76.78)** | **-2.98 (-3.32 to -2.65)** |
| **United Arab Emirates** | **0.84974** | **High SDI** | **3 (0 to 20)** | **11 (0 to 67)** | **0.77 (0 to 4.73)** | **0.39 (0 to 2.41)** | **-0.8 (-1.32 to -0.29)** | **114 (0 to 667)** | **379 (0 to 2229)** | **19 (0 to 113.86)** | **8.12 (0 to 48.99)** | **-1.68 (-2.15 to -1.21)** |
| **Yemen** | **0.45354** | **Low SDI** | **65 (0 to 411)** | **136 (0 to 890)** | **1.3 (0 to 8.24)** | **0.98 (0 to 6.44)** | **-1.14 (-1.23 to -1.04)** | **1978 (0 to 12384)** | **3969 (0 to 26028)** | **34.98 (0 to 220.66)** | **24.51 (0 to 160.97)** | **-1.4 (-1.51 to -1.29)** |
| **South Asia** | **0.559643** | **NA** | **3631 (0 to 18683)** | **6499 (0 to 32700)** | **0.62 (0 to 3.21)** | **0.45 (0 to 2.25)** | **-0.95 (-1.03 to -0.86)** | **112571 (0 to 573788)** | **180806 (0 to 908078)** | **17.02 (0 to 87.29)** | **11.45 (0 to 57.6)** | **-1.19 (-1.26 to -1.11)** |
| **South Asia** | **0.559643** | **NA** | **3631 (0 to 18683)** | **6499 (0 to 32700)** | **0.62 (0 to 3.21)** | **0.45 (0 to 2.25)** | **-0.95 (-1.03 to -0.86)** | **112571 (0 to 573788)** | **180806 (0 to 908078)** | **17.02 (0 to 87.29)** | **11.45 (0 to 57.6)** | **-1.19 (-1.26 to -1.11)** |
| **Afghanistan** | **0.335068** | **Low SDI** | **142 (0 to 885)** | **162 (0 to 1025)** | **2.07 (0 to 12.89)** | **1.59 (0 to 10.32)** | **-1.05 (-1.29 to -0.8)** | **4108 (0 to 25272)** | **5393 (0 to 34028)** | **57.31 (0 to 356.12)** | **42.9 (0 to 275.01)** | **-1.16 (-1.41 to -0.91)** |
| **Bangladesh** | **0.493106** | **Low-middle SDI** | **356 (0 to 1827)** | **570 (0 to 2941)** | **0.75 (0 to 3.87)** | **0.43 (0 to 2.2)** | **-1.76 (-1.99 to -1.52)** | **10608 (0 to 54919)** | **14956 (0 to 77505)** | **20.37 (0 to 104.95)** | **10.42 (0 to 53.93)** | **-2.01 (-2.18 to -1.83)** |
| **Bhutan** | **0.476725** | **Low-middle SDI** | **2 (0 to 8)** | **3 (0 to 14)** | **0.62 (0 to 3.3)** | **0.44 (0 to 2.4)** | **-0.97 (-1.09 to -0.85)** | **48 (0 to 254)** | **66 (0 to 362)** | **16.61 (0 to 88.33)** | **10.49 (0 to 57.2)** | **-1.41 (-1.54 to -1.29)** |
| **India** | **0.577738** | **NA** | **2989 (0 to 15541)** | **5368 (0 to 27164)** | **0.63 (0 to 3.29)** | **0.45 (0 to 2.31)** | **-0.91 (-1.04 to -0.77)** | **93864 (0 to 482095)** | **149555 (0 to 754254)** | **17.43 (0 to 90.46)** | **11.76 (0 to 59.4)** | **-1.16 (-1.27 to -1.05)** |
| **Nepal** | **0.433953** | **Low SDI** | **61 (0 to 322)** | **110 (0 to 558)** | **0.66 (0 to 3.49)** | **0.49 (0 to 2.5)** | **-0.71 (-0.98 to -0.44)** | **1883 (0 to 10104)** | **2951 (0 to 15051)** | **17.54 (0 to 93.06)** | **12.08 (0 to 61.32)** | **-1.01 (-1.3 to -0.73)** |
| **Pakistan** | **0.504276** | **NA** | **223 (0 to 1162)** | **448 (0 to 2198)** | **0.41 (0 to 2.15)** | **0.38 (0 to 1.89)** | **-0.44 (-0.71 to -0.17)** | **6169 (0 to 31945)** | **13278 (0 to 65439)** | **10.26 (0 to 53.22)** | **9.51 (0 to 46.81)** | **-0.47 (-0.76 to -0.18)** |
| **Central Sub-Saharan Africa** | **0.484518** | **NA** | **142 (0 to 838)** | **262 (0 to 1544)** | **0.68 (0 to 4.03)** | **0.51 (0 to 2.99)** | **-0.96 (-1 to -0.92)** | **4237 (0 to 24980)** | **7864 (0 to 46176)** | **16.97 (0 to 100.02)** | **12.54 (0 to 73.89)** | **-1.02 (-1.06 to -0.98)** |
| **Angola** | **0.482946** | **Low-middle SDI** | **32 (0 to 195)** | **64 (0 to 368)** | **0.88 (0 to 5.3)** | **0.6 (0 to 3.35)** | **-1.44 (-1.51 to -1.37)** | **996 (0 to 6061)** | **1938 (0 to 11243)** | **22.24 (0 to 135.36)** | **14.38 (0 to 81.79)** | **-1.6 (-1.67 to -1.53)** |
| **Central African Republic** | **0.311027** | **Low SDI** | **12 (0 to 68)** | **19 (0 to 110)** | **1.1 (0 to 6.27)** | **0.89 (0 to 5.12)** | **-0.77 (-0.85 to -0.69)** | **375 (0 to 2129)** | **610 (0 to 3522)** | **28.67 (0 to 162.1)** | **22.89 (0 to 132.24)** | **-0.84 (-0.92 to -0.75)** |
| **Congo** | **0.586909** | **Low-middle SDI** | **9 (0 to 55)** | **15 (0 to 85)** | **0.94 (0 to 5.41)** | **0.61 (0 to 3.39)** | **-1.68 (-1.82 to -1.55)** | **278 (0 to 1607)** | **452 (0 to 2618)** | **23.83 (0 to 138.15)** | **14.53 (0 to 82.32)** | **-1.9 (-2.05 to -1.74)** |
| **Democratic Republic of the Congo** | **0.390178** | **Low SDI** | **82 (0 to 489)** | **157 (0 to 950)** | **0.57 (0 to 3.4)** | **0.46 (0 to 2.79)** | **-0.69 (-0.73 to -0.64)** | **2422 (0 to 14460)** | **4659 (0 to 28099)** | **14.1 (0 to 83.72)** | **11.2 (0 to 67.87)** | **-0.7 (-0.75 to -0.65)** |
| **Equatorial Guinea** | **0.663978** | **Middle SDI** | **2 (0 to 11)** | **2 (0 to 12)** | **0.97 (0 to 5.72)** | **0.45 (0 to 2.52)** | **-2.81 (-3.12 to -2.5)** | **52 (0 to 314)** | **61 (0 to 348)** | **24.64 (0 to 147.24)** | **10.35 (0 to 59.07)** | **-3.17 (-3.52 to -2.82)** |
| **Gabon** | **0.639081** | **Middle SDI** | **4 (0 to 25)** | **5 (0 to 30)** | **0.79 (0 to 4.51)** | **0.55 (0 to 3.04)** | **-1.32 (-1.44 to -1.19)** | **112 (0 to 645)** | **144 (0 to 825)** | **19.19 (0 to 109.79)** | **12.75 (0 to 72.33)** | **-1.44 (-1.57 to -1.31)** |
| **Eastern Sub-Saharan Africa** | **0.412188** | **NA** | **658 (0 to 3325)** | **852 (0 to 4457)** | **0.9 (0 to 4.5)** | **0.54 (0 to 2.79)** | **-1.9 (-1.99 to -1.81)** | **19716 (0 to 99793)** | **24470 (0 to 129515)** | **23.37 (0 to 118.12)** | **12.94 (0 to 67.73)** | **-2.21 (-2.32 to -2.11)** |
| **Burundi** | **0.291289** | **Low SDI** | **24 (0 to 124)** | **28 (0 to 146)** | **1.04 (0 to 5.38)** | **0.62 (0 to 3.18)** | **-2.16 (-2.36 to -1.96)** | **682 (0 to 3577)** | **823 (0 to 4343)** | **26.97 (0 to 140.64)** | **14.94 (0 to 77.39)** | **-2.46 (-2.68 to -2.24)** |
| **Comoros** | **0.476956** | **Low-middle SDI** | **2 (0 to 8)** | **3 (0 to 13)** | **0.8 (0 to 4.14)** | **0.55 (0 to 2.83)** | **-1.42 (-1.54 to -1.3)** | **45 (0 to 237)** | **68 (0 to 357)** | **20.45 (0 to 108.08)** | **13.11 (0 to 68.4)** | **-1.72 (-1.87 to -1.56)** |
| **Djibouti** | **0.4892** | **Low-middle SDI** | **1 (0 to 5)** | **3 (0 to 18)** | **0.75 (0 to 3.87)** | **0.6 (0 to 3.11)** | **-0.93 (-1.06 to -0.8)** | **30 (0 to 161)** | **101 (0 to 557)** | **18.73 (0 to 97.98)** | **13.99 (0 to 74.78)** | **-1.14 (-1.29 to -0.98)** |
| **Eritrea** | **0.404572** | **Low SDI** | **13 (0 to 67)** | **21 (0 to 113)** | **1.14 (0 to 5.88)** | **0.8 (0 to 4.29)** | **-1.2 (-1.24 to -1.16)** | **441 (0 to 2222)** | **642 (0 to 3512)** | **30.92 (0 to 160.17)** | **19.77 (0 to 107.3)** | **-1.52 (-1.57 to -1.47)** |
| **Ethiopia** | **0.360728** | **NA** | **260 (0 to 1327)** | **219 (0 to 1162)** | **1.3 (0 to 6.63)** | **0.53 (0 to 2.77)** | **-3.44 (-3.64 to -3.24)** | **8248 (0 to 41968)** | **6183 (0 to 33134)** | **35.48 (0 to 180.82)** | **12.63 (0 to 67.11)** | **-3.9 (-4.12 to -3.68)** |
| **Kenya** | **0.524783** | **NA** | **41 (0 to 221)** | **101 (0 to 581)** | **0.52 (0 to 2.79)** | **0.48 (0 to 2.73)** | **0.13 (-0.08 to 0.34)** | **1144 (0 to 6300)** | **2828 (0 to 16302)** | **12.73 (0 to 69.29)** | **11.21 (0 to 64.41)** | **-0.05 (-0.28 to 0.18)** |
| **Madagascar** | **0.401385** | **Low SDI** | **36 (0 to 194)** | **53 (0 to 277)** | **0.73 (0 to 3.9)** | **0.51 (0 to 2.6)** | **-1.26 (-1.32 to -1.2)** | **1055 (0 to 5694)** | **1652 (0 to 8910)** | **18.8 (0 to 100.96)** | **12.5 (0 to 64.66)** | **-1.4 (-1.46 to -1.33)** |
| **Malawi** | **0.381986** | **Low SDI** | **12 (0 to 61)** | **18 (0 to 92)** | **0.33 (0 to 1.66)** | **0.25 (0 to 1.29)** | **-1.19 (-1.44 to -0.94)** | **357 (0 to 1775)** | **515 (0 to 2670)** | **8.34 (0 to 41.56)** | **6.21 (0 to 31.89)** | **-1.32 (-1.6 to -1.04)** |
| **Mauritius** | **0.717977** | **High-middle SDI** | **9 (0 to 47)** | **15 (0 to 73)** | **1.33 (0 to 6.68)** | **0.82 (0 to 4.08)** | **-2.31 (-2.68 to -1.95)** | **257 (0 to 1299)** | **376 (0 to 1858)** | **32.77 (0 to 165.01)** | **20.67 (0 to 102.35)** | **-2.25 (-2.64 to -1.87)** |
| **Mozambique** | **0.327475** | **Low SDI** | **36 (0 to 177)** | **61 (0 to 319)** | **0.7 (0 to 3.42)** | **0.64 (0 to 3.34)** | **0.14 (-0.02 to 0.3)** | **954 (0 to 4646)** | **1633 (0 to 8773)** | **15.65 (0 to 75.82)** | **14.21 (0 to 74.86)** | **0.14 (-0.04 to 0.32)** |
| **Rwanda** | **0.43614** | **Low SDI** | **31 (0 to 157)** | **32 (0 to 164)** | **1.11 (0 to 5.57)** | **0.55 (0 to 2.81)** | **-3.32 (-3.69 to -2.94)** | **939 (0 to 4805)** | **889 (0 to 4708)** | **29.26 (0 to 148.19)** | **12.83 (0 to 66.63)** | **-3.8 (-4.22 to -3.39)** |
| **Seychelles** | **0.727579** | **High-middle SDI** | **0 (0 to 2)** | **1 (0 to 3)** | **0.87 (0 to 4.36)** | **0.48 (0 to 2.42)** | **-1.78 (-1.92 to -1.64)** | **12 (0 to 63)** | **14 (0 to 70)** | **22.14 (0 to 112.86)** | **11.35 (0 to 57.09)** | **-2.04 (-2.18 to -1.91)** |
| **Somalia** | **0.077434** | **Low SDI** | **28 (0 to 144)** | **56 (0 to 289)** | **1.2 (0 to 5.98)** | **0.94 (0 to 4.85)** | **-0.89 (-0.96 to -0.81)** | **931 (0 to 4782)** | **1762 (0 to 9244)** | **31.52 (0 to 159.2)** | **23.87 (0 to 123.47)** | **-1.05 (-1.13 to -0.97)** |
| **United Republic of Tanzania** | **0.448566** | **Low SDI** | **81 (0 to 398)** | **122 (0 to 616)** | **0.78 (0 to 3.8)** | **0.5 (0 to 2.53)** | **-1.6 (-1.68 to -1.51)** | **2295 (0 to 11355)** | **3414 (0 to 17274)** | **19.44 (0 to 95.7)** | **12.12 (0 to 60.89)** | **-1.74 (-1.83 to -1.65)** |
| **Uganda** | **0.426554** | **Low SDI** | **46 (0 to 234)** | **69 (0 to 356)** | **0.75 (0 to 3.82)** | **0.5 (0 to 2.58)** | **-1.94 (-2.23 to -1.65)** | **1271 (0 to 6469)** | **1961 (0 to 10170)** | **18.41 (0 to 93.72)** | **11.95 (0 to 61.49)** | **-2.13 (-2.46 to -1.8)** |
| **Zambia** | **0.51023** | **Low-middle SDI** | **24 (0 to 122)** | **39 (0 to 209)** | **0.86 (0 to 4.45)** | **0.59 (0 to 3.1)** | **-1.68 (-1.94 to -1.42)** | **697 (0 to 3609)** | **1167 (0 to 6406)** | **21.8 (0 to 112.43)** | **14.42 (0 to 77.58)** | **-1.85 (-2.13 to -1.57)** |
| **Southern Sub-Saharan Africa** | **0.643348** | **NA** | **148 (0 to 796)** | **255 (0 to 1387)** | **0.55 (0 to 3)** | **0.45 (0 to 2.48)** | **-0.69 (-1.01 to -0.37)** | **4431 (0 to 23681)** | **7340 (0 to 39115)** | **14.62 (0 to 78.11)** | **11.52 (0 to 62.18)** | **-0.75 (-1.08 to -0.43)** |
| **Botswana** | **0.643078** | **Middle SDI** | **4 (0 to 22)** | **5 (0 to 28)** | **0.72 (0 to 4.04)** | **0.36 (0 to 2.01)** | **-2.34 (-2.55 to -2.13)** | **113 (0 to 641)** | **142 (0 to 796)** | **18.35 (0 to 104.67)** | **8.62 (0 to 48.6)** | **-2.58 (-2.82 to -2.33)** |
| **Lesotho** | **0.511571** | **Low-middle SDI** | **5 (0 to 29)** | **9 (0 to 50)** | **0.64 (0 to 3.64)** | **0.81 (0 to 4.67)** | **1.49 (1.07 to 1.92)** | **140 (0 to 776)** | **262 (0 to 1462)** | **15.89 (0 to 88.16)** | **21.79 (0 to 122.95)** | **1.76 (1.29 to 2.24)** |
| **Namibia** | **0.618074** | **Low-middle SDI** | **2 (0 to 8)** | **2 (0 to 13)** | **0.24 (0 to 1.28)** | **0.18 (0 to 0.97)** | **-1.15 (-1.45 to -0.84)** | **45 (0 to 241)** | **72 (0 to 396)** | **6.22 (0 to 33.55)** | **4.58 (0 to 25)** | **-1.21 (-1.56 to -0.86)** |
| **South Africa** | **0.681292** | **NA** | **102 (0 to 568)** | **166 (0 to 931)** | **0.49 (0 to 2.72)** | **0.37 (0 to 2.09)** | **-1.14 (-1.45 to -0.83)** | **3161 (0 to 17149)** | **4638 (0 to 25752)** | **13.44 (0 to 73.49)** | **9.27 (0 to 51.66)** | **-1.4 (-1.68 to -1.11)** |
| **Eswatini** | **0.586217** | **Low-middle SDI** | **2 (0 to 12)** | **3 (0 to 20)** | **0.77 (0 to 4.28)** | **0.62 (0 to 3.49)** | **-0.27 (-0.82 to 0.28)** | **64 (0 to 356)** | **110 (0 to 621)** | **19.66 (0 to 111.25)** | **16.45 (0 to 93.58)** | **-0.12 (-0.75 to 0.52)** |
| **Zimbabwe** | **0.475577** | **Low-middle SDI** | **33 (0 to 175)** | **69 (0 to 372)** | **0.88 (0 to 4.65)** | **1.02 (0 to 5.37)** | **1.11 (0.62 to 1.59)** | **909 (0 to 4836)** | **2116 (0 to 11467)** | **20.87 (0 to 110.33)** | **26.08 (0 to 140.73)** | **1.38 (0.81 to 1.95)** |
| **Western Sub-Saharan Africa** | **0.446421** | **NA** | **479 (0 to 2559)** | **880 (0 to 4609)** | **0.57 (0 to 3.06)** | **0.49 (0 to 2.54)** | **-0.28 (-0.37 to -0.2)** | **13169 (0 to 70651)** | **24102 (0 to 126886)** | **14.02 (0 to 75.09)** | **11.32 (0 to 59.28)** | **-0.48 (-0.55 to -0.4)** |
| **Benin** | **0.374522** | **Low SDI** | **18 (0 to 95)** | **34 (0 to 179)** | **0.94 (0 to 4.96)** | **0.73 (0 to 3.79)** | **-0.55 (-0.72 to -0.38)** | **468 (0 to 2485)** | **918 (0 to 4782)** | **22.57 (0 to 119.19)** | **16.67 (0 to 86.71)** | **-0.75 (-0.92 to -0.58)** |
| **Burkina Faso** | **0.284471** | **Low SDI** | **41 (0 to 223)** | **74 (0 to 417)** | **1.03 (0 to 5.5)** | **0.87 (0 to 4.86)** | **-0.21 (-0.38 to -0.03)** | **1120 (0 to 6134)** | **1968 (0 to 11098)** | **24.67 (0 to 134.44)** | **20.06 (0 to 112.79)** | **-0.36 (-0.55 to -0.17)** |
| **Cameroon** | **0.480365** | **Low-middle SDI** | **38 (0 to 205)** | **87 (0 to 495)** | **0.92 (0 to 4.93)** | **0.76 (0 to 4.29)** | **-0.39 (-0.51 to -0.26)** | **1054 (0 to 5692)** | **2438 (0 to 13967)** | **22.07 (0 to 118.96)** | **17.73 (0 to 100.65)** | **-0.51 (-0.64 to -0.37)** |
| **Cabo Verde** | **0.533601** | **Low-middle SDI** | **5 (0 to 29)** | **8 (0 to 41)** | **2.31 (0 to 12.26)** | **1.78 (0 to 9.63)** | **-1.1 (-1.58 to -0.61)** | **118 (0 to 622)** | **170 (0 to 922)** | **52.51 (0 to 276.31)** | **37.72 (0 to 205.61)** | **-1.29 (-1.72 to -0.85)** |
| **Chad** | **0.243517** | **Low SDI** | **23 (0 to 126)** | **53 (0 to 294)** | **0.86 (0 to 4.63)** | **1 (0 to 5.48)** | **0.72 (0.59 to 0.85)** | **606 (0 to 3272)** | **1473 (0 to 8136)** | **20.84 (0 to 112.96)** | **23.57 (0 to 130.09)** | **0.61 (0.48 to 0.74)** |
| **C么te d'Ivoire** | **0.424541** | **Low SDI** | **12 (0 to 64)** | **27 (0 to 143)** | **0.34 (0 to 1.78)** | **0.26 (0 to 1.35)** | **-0.81 (-0.99 to -0.63)** | **374 (0 to 1936)** | **787 (0 to 4235)** | **8.23 (0 to 42.67)** | **6.17 (0 to 32.87)** | **-0.88 (-1.06 to -0.69)** |
| **Gambia** | **0.410077** | **Low SDI** | **1 (0 to 6)** | **2 (0 to 13)** | **0.3 (0 to 1.64)** | **0.26 (0 to 1.36)** | **-0.54 (-0.67 to -0.41)** | **29 (0 to 161)** | **67 (0 to 356)** | **7.48 (0 to 41.06)** | **6.22 (0 to 33.07)** | **-0.66 (-0.82 to -0.5)** |
| **Ghana** | **0.563348** | **Low-middle SDI** | **42 (0 to 222)** | **91 (0 to 470)** | **0.73 (0 to 3.87)** | **0.61 (0 to 3.13)** | **-0.38 (-0.45 to -0.31)** | **1188 (0 to 6359)** | **2465 (0 to 12776)** | **17.48 (0 to 92.91)** | **13.81 (0 to 71.2)** | **-0.55 (-0.61 to -0.49)** |
| **Guinea** | **0.336555** | **Low SDI** | **25 (0 to 136)** | **39 (0 to 209)** | **0.79 (0 to 4.23)** | **0.71 (0 to 3.81)** | **-0.13 (-0.27 to 0.01)** | **682 (0 to 3624)** | **1074 (0 to 5838)** | **19.73 (0 to 105.27)** | **17.44 (0 to 94.45)** | **-0.18 (-0.33 to -0.03)** |
| **Guinea-Bissau** | **0.353448** | **Low SDI** | **5 (0 to 30)** | **8 (0 to 43)** | **1.42 (0 to 7.8)** | **1.18 (0 to 6.36)** | **-0.18 (-0.35 to -0.01)** | **160 (0 to 898)** | **239 (0 to 1304)** | **36.56 (0 to 203.01)** | **28.79 (0 to 155.74)** | **-0.39 (-0.55 to -0.22)** |
| **Liberia** | **0.353229** | **Low SDI** | **9 (0 to 49)** | **14 (0 to 79)** | **0.83 (0 to 4.48)** | **0.75 (0 to 4.11)** | **-0.23 (-0.38 to -0.07)** | **234 (0 to 1280)** | **408 (0 to 2249)** | **19.57 (0 to 106.49)** | **17.25 (0 to 95.14)** | **-0.33 (-0.5 to -0.16)** |
| **Mali** | **0.271176** | **Low SDI** | **61 (0 to 329)** | **104 (0 to 546)** | **1.62 (0 to 8.69)** | **1.25 (0 to 6.52)** | **-0.59 (-0.7 to -0.47)** | **1791 (0 to 9508)** | **2973 (0 to 15640)** | **41.04 (0 to 219.42)** | **30.42 (0 to 159.74)** | **-0.73 (-0.85 to -0.62)** |
| **Mauritania** | **0.495267** | **Low-middle SDI** | **9 (0 to 46)** | **13 (0 to 71)** | **0.9 (0 to 4.84)** | **0.67 (0 to 3.58)** | **-0.82 (-1.08 to -0.56)** | **223 (0 to 1199)** | **326 (0 to 1762)** | **21.58 (0 to 115.65)** | **14.69 (0 to 79.36)** | **-1.1 (-1.36 to -0.85)** |
| **Niger** | **0.17031** | **Low SDI** | **25 (0 to 133)** | **62 (0 to 339)** | **0.97 (0 to 5.22)** | **0.85 (0 to 4.69)** | **-0.09 (-0.26 to 0.08)** | **718 (0 to 3878)** | **1674 (0 to 9215)** | **23.55 (0 to 126.82)** | **19.28 (0 to 105.25)** | **-0.33 (-0.51 to -0.16)** |
| **Nigeria** | **0.503699** | **NA** | **108 (0 to 584)** | **150 (0 to 825)** | **0.26 (0 to 1.4)** | **0.18 (0 to 0.97)** | **-1.07 (-1.19 to -0.95)** | **2934 (0 to 15757)** | **4072 (0 to 22551)** | **6.24 (0 to 33.69)** | **4.11 (0 to 22.85)** | **-1.34 (-1.45 to -1.23)** |
| **Sao Tome and Principe** | **0.503306** | **Low-middle SDI** | **1 (0 to 4)** | **1 (0 to 6)** | **1.23 (0 to 6.42)** | **1.12 (0 to 5.84)** | **-0.22 (-0.37 to -0.07)** | **18 (0 to 93)** | **27 (0 to 140)** | **27.16 (0 to 143.37)** | **24.09 (0 to 124.39)** | **-0.44 (-0.63 to -0.26)** |
| **Senegal** | **0.409005** | **Low SDI** | **28 (0 to 149)** | **56 (0 to 306)** | **0.91 (0 to 4.85)** | **0.78 (0 to 4.27)** | **-0.18 (-0.41 to 0.05)** | **745 (0 to 4015)** | **1443 (0 to 8004)** | **21.85 (0 to 117.75)** | **17.81 (0 to 98.07)** | **-0.37 (-0.6 to -0.14)** |
| **Sierra Leone** | **0.359009** | **Low SDI** | **16 (0 to 89)** | **27 (0 to 149)** | **0.84 (0 to 4.56)** | **0.77 (0 to 4.24)** | **0.18 (-0.01 to 0.37)** | **419 (0 to 2319)** | **728 (0 to 4056)** | **19.86 (0 to 109.52)** | **17.95 (0 to 99.25)** | **0.15 (-0.05 to 0.35)** |
| **Togo** | **0.410016** | **Low SDI** | **10 (0 to 54)** | **30 (0 to 170)** | **0.88 (0 to 4.73)** | **0.86 (0 to 4.82)** | **0.26 (0.1 to 0.41)** | **289 (0 to 1561)** | **853 (0 to 4937)** | **21.31 (0 to 114.32)** | **20.31 (0 to 115.37)** | **0.16 (0 to 0.32)** |
| **American Samoa** | **0.726268** | **High-middle SDI** | **0 (0 to 2)** | **1 (0 to 3)** | **1.46 (0 to 7.89)** | **1.26 (0 to 6.61)** | **-0.38 (-0.46 to -0.3)** | **8 (0 to 48)** | **14 (0 to 75)** | **33.5 (0 to 190.16)** | **28.69 (0 to 156.55)** | **-0.43 (-0.52 to -0.35)** |
| **Bermuda** | **0.82132** | **High SDI** | **1 (0 to 3)** | **1 (0 to 3)** | **1.01 (0 to 5.26)** | **0.37 (0 to 2.01)** | **-3.11 (-3.34 to -2.87)** | **14 (0 to 74)** | **10 (0 to 56)** | **22.38 (0 to 116.93)** | **7.89 (0 to 42.51)** | **-3.23 (-3.46 to -2.99)** |
| **Cook Islands** | **0.778252** | **High-middle SDI** | **0 (0 to 1)** | **0 (0 to 1)** | **0.93 (0 to 4.74)** | **0.49 (0 to 2.48)** | **-2 (-2.13 to -1.87)** | **3 (0 to 14)** | **3 (0 to 14)** | **20.67 (0 to 107.53)** | **11.12 (0 to 57.42)** | **-1.91 (-2.08 to -1.74)** |
| **Greenland** | **0.83564** | **High SDI** | **1 (0 to 3)** | **0 (0 to 2)** | **1.54 (0 to 7.86)** | **0.68 (0 to 3.53)** | **-2.6 (-2.74 to -2.46)** | **16 (0 to 83)** | **12 (0 to 64)** | **40 (0 to 203.64)** | **17 (0 to 87.85)** | **-2.75 (-2.87 to -2.63)** |
| **Guam** | **0.802168** | **High-middle SDI** | **0 (0 to 2)** | **1 (0 to 4)** | **0.74 (0 to 3.74)** | **0.38 (0 to 1.96)** | **-1.66 (-2.1 to -1.21)** | **12 (0 to 65)** | **22 (0 to 113)** | **15.67 (0 to 80.55)** | **10.64 (0 to 56.35)** | **-0.85 (-1.23 to -0.47)** |
| **Monaco** | **0.909519** | **High SDI** | **1 (0 to 3)** | **1 (0 to 3)** | **0.89 (0 to 4.66)** | **0.53 (0 to 2.81)** | **-1.65 (-1.78 to -1.53)** | **13 (0 to 65)** | **10 (0 to 53)** | **19.69 (0 to 102.73)** | **11.58 (0 to 60.31)** | **-1.71 (-1.83 to -1.59)** |
| **Nauru** | **0.62755** | **Middle SDI** | **0 (0 to 0)** | **0 (0 to 0)** | **2.07 (0 to 10.65)** | **1.64 (0 to 8.29)** | **-0.84 (-1.08 to -0.59)** | **3 (0 to 15)** | **3 (0 to 15)** | **51.4 (0 to 269.18)** | **41.59 (0 to 217.06)** | **-0.79 (-1.07 to -0.51)** |
| **Niue** | **0.726219** | **High-middle SDI** | **0 (0 to 0)** | **0 (0 to 0)** | **1.16 (0 to 5.68)** | **0.92 (0 to 4.57)** | **-0.92 (-0.97 to -0.87)** | **1 (0 to 3)** | **0 (0 to 2)** | **26.61 (0 to 133.54)** | **20.96 (0 to 106.92)** | **-1.01 (-1.08 to -0.94)** |
| **Northern Mariana Islands** | **0.777505** | **High-middle SDI** | **0 (0 to 1)** | **0 (0 to 2)** | **1.38 (0 to 7.05)** | **1.1 (0 to 5.55)** | **-0.68 (-0.95 to -0.42)** | **6 (0 to 35)** | **13 (0 to 64)** | **30.52 (0 to 162.34)** | **24.14 (0 to 123.29)** | **-0.66 (-0.94 to -0.38)** |
| **Palau** | **0.75459** | **High-middle SDI** | **0 (0 to 1)** | **0 (0 to 1)** | **1.97 (0 to 10.21)** | **1.49 (0 to 7.57)** | **-0.76 (-0.83 to -0.68)** | **5 (0 to 26)** | **8 (0 to 42)** | **45.69 (0 to 245.01)** | **35 (0 to 183.43)** | **-0.74 (-0.8 to -0.68)** |
| **Puerto Rico** | **0.824544** | **High SDI** | **33 (0 to 170)** | **24 (0 to 126)** | **0.93 (0 to 4.77)** | **0.32 (0 to 1.69)** | **-3.7 (-3.84 to -3.56)** | **721 (0 to 3733)** | **470 (0 to 2477)** | **19.94 (0 to 103.16)** | **7.39 (0 to 39.46)** | **-3.46 (-3.6 to -3.32)** |
| **Saint Kitts and Nevis** | **0.756333** | **High-middle SDI** | **0 (0 to 3)** | **0 (0 to 2)** | **1.31 (0 to 6.72)** | **0.69 (0 to 3.84)** | **-1.77 (-1.95 to -1.59)** | **11 (0 to 56)** | **11 (0 to 58)** | **30.25 (0 to 155.63)** | **14.9 (0 to 82.49)** | **-2.05 (-2.25 to -1.85)** |
| **San Marino** | **0.887884** | **High SDI** | **1 (0 to 4)** | **1 (0 to 3)** | **2.02 (0 to 10.43)** | **0.71 (0 to 3.94)** | **-2.54 (-2.86 to -2.22)** | **14 (0 to 74)** | **10 (0 to 59)** | **41.88 (0 to 218.39)** | **14.9 (0 to 83.83)** | **-2.55 (-2.84 to -2.26)** |
| **Tokelau** | **0.687018** | **Middle SDI** | **0 (0 to 0)** | **0 (0 to 0)** | **1.39 (0 to 7.41)** | **0.86 (0 to 4.39)** | **-1.66 (-1.69 to -1.64)** | **0 (0 to 2)** | **0 (0 to 1)** | **32.03 (0 to 175.75)** | **19.87 (0 to 102.66)** | **-1.68 (-1.72 to -1.64)** |
| **Tuvalu** | **0.578627** | **Low-middle SDI** | **0 (0 to 1)** | **0 (0 to 1)** | **1.77 (0 to 9.49)** | **1.18 (0 to 6.12)** | **-1.22 (-1.29 to -1.15)** | **3 (0 to 17)** | **3 (0 to 16)** | **43.29 (0 to 237.51)** | **28.45 (0 to 151.15)** | **-1.27 (-1.36 to -1.19)** |
| **United States Virgin Islands** | **0.822988** | **High SDI** | **1 (0 to 4)** | **1 (0 to 4)** | **0.91 (0 to 4.64)** | **0.42 (0 to 2.15)** | **-2.43 (-2.61 to -2.24)** | **19 (0 to 98)** | **16 (0 to 83)** | **20.88 (0 to 109.62)** | **9.82 (0 to 51.14)** | **-2.28 (-2.45 to -2.12)** |
| **South Sudan** | **0.278378** | **Low SDI** | **22 (0 to 116)** | **27 (0 to 144)** | **0.9 (0 to 4.63)** | **0.75 (0 to 3.94)** | **-0.81 (-0.97 to -0.64)** | **615 (0 to 3225)** | **810 (0 to 4325)** | **22.53 (0 to 117.5)** | **18.49 (0 to 97)** | **-0.91 (-1.12 to -0.7)** |
| **Sudan** | **0.542748** | **Low-middle SDI** | **104 (0 to 622)** | **150 (0 to 953)** | **1.1 (0 to 6.66)** | **0.78 (0 to 5)** | **-1.16 (-1.22 to -1.11)** | **3035 (0 to 17978)** | **4404 (0 to 27600)** | **29.18 (0 to 174.63)** | **19.54 (0 to 123.65)** | **-1.36 (-1.41 to -1.3)** |
| **Georgia** | **0.847268** | **High SDI** | **88 (0 to 434)** | **55 (0 to 281)** | **1.4 (0 to 6.95)** | **0.92 (0 to 4.72)** | **-0.31 (-0.77 to 0.16)** | **2406 (0 to 11946)** | **1282 (0 to 6631)** | **38.06 (0 to 188.76)** | **22.66 (0 to 117.21)** | **-0.69 (-1.11 to -0.27)** |
| **Niger** | **0.425189** | **Low SDI** | **25 (0 to 133)** | **62 (0 to 339)** | **0.97 (0 to 5.22)** | **0.85 (0 to 4.69)** | **-0.09 (-0.26 to 0.08)** | **718 (0 to 3878)** | **1674 (0 to 9215)** | **23.55 (0 to 126.82)** | **19.28 (0 to 105.25)** | **-0.33 (-0.51 to -0.16)** |

**Abbreviations: ASR, age-standardized rate; DALYs, disability-adjusted life-years; SDI, sociodemographic index; GBD, Global Burden of Diseases, Injuries, and Risk Factors Study; EAPC, estimated annual percentage change; UIs, uncertainty intervals; CI, conﬁdence interval.**

**Supplemental Table4. The APC and AAPC in ASR of Gastric cancer due to high sodium diet mortality for both sexes from 1990 to 2021.**

| **Location** | **Segment** | **APC** | **P Value** | **AAPC (1990-2021)** | **P Value** |
| --- | --- | --- | --- | --- | --- |
| **Global** | **1990-1998** | **-2.1379 (-2.2318 to -2.0439)** | **0** | **-2.1735**  **(-2.2982 to -2.0486)** | **0** |
| **Global** | **1998-2004** | **-1.1092 (-1.3037 to -0.9143)** | **0** |  |  |
| **Global** | **2004-2007** | **-4.0983 (-4.9301 to -3.2593)** | **0** |  |  |
| **Global** | **2007-2010** | **-2.2721 (-3.1282 to -1.4084)** | **5.40E-05** |  |  |
| **Global** | **2010-2015** | **-2.9044 (-3.1744 to -2.6337)** | **0** |  |  |
| **Global** | **2015-2021** | **-1.6455 (-1.7903 to -1.5005)** | **0** |  |  |
| **High-middle SDI** | **1990-1998** | **-2.4532 (-2.6196 to -2.2864)** | **0** | **-2.327 (-2.4969 to -2.1569)** | **0** |
| **High-middle SDI** | **1998-2004** | **-0.4895 (-0.8369 to -0.1409)** | **0.008606** |  |  |
| **High-middle SDI** | **2004-2007** | **-4.9636 (-6.4349 to -3.4692)** | **2.00E-06** |  |  |
| **High-middle SDI** | **2007-2015** | **-3.1068 (-3.3096 to -2.9035)** | **0** |  |  |
| **High-middle SDI** | **2015-2021** | **-1.5961 (-1.8529 to -1.3386)** | **0** |  |  |
| **High SDI** | **1990-1998** | **-2.4867 (-2.6095 to -2.3637)** | **0** | **-2.6367 (-2.7141 to -2.5593)** | **0** |
| **High SDI** | **1998-2019** | **-2.799 (-2.8297 to -2.7682)** | **0** |  |  |
| **High SDI** | **2019-2021** | **-1.5251 (-2.6492 to -0.3879)** | **0.010831** |  |  |
| **Low-middle SDI** | **1990-2005** | **-0.8844 (-0.9609 to -0.8079)** | **0** | **-0.9971 (-1.1726 to -0.8213)** | **0** |
| **Low-middle SDI** | **2005-2008** | **-0.1873 (-2.0125 to 1.6719)** | **0.835682** |  |  |
| **Low-middle SDI** | **2008-2021** | **-1.3127 (-1.4076 to -1.2177)** | **0** |  |  |
| **Low SDI** | **1990-1997** | **-0.6637 (-0.8931 to -0.4336)** | **6.00E-06** | **-1.1366 (-1.2391 to -1.034)** | **0** |
| **Low SDI** | **1997-2005** | **-1.7885 (-2.017 to -1.5595)** | **0** |  |  |
| **Low SDI** | **2005-2015** | **-0.6374 (-0.7935 to -0.481)** | **0** |  |  |
| **Low SDI** | **2015-2021** | **-1.6436 (-1.9311 to -1.3553)** | **0** |  |  |
| **Middle SDI** | **1990-1999** | **-2.0874 (-2.1851 to -1.9897)** | **0** | **-2.3478 (-2.5042 to -2.1913)** | **0** |
| **Middle SDI** | **1999-2004** | **-0.6279 (-0.9679 to -0.2867)** | **0.001374** |  |  |
| **Middle SDI** | **2004-2007** | **-5.3108 (-6.3285 to -4.282)** | **0** |  |  |
| **Middle SDI** | **2007-2010** | **-2.1402 (-3.2036 to -1.0652)** | **7.41E-04** |  |  |
| **Middle SDI** | **2010-2015** | **-3.4946 (-3.8273 to -3.1608)** | **0** |  |  |
| **Middle SDI** | **2015-2021** | **-1.7953 (-1.9742 to -1.616)** | **0** |  |  |
| **Central Sub-Saharan Africa** | **1990-1999** | **-0.6149 (-0.753 to -0.4767)** | **0** | **-0.8972 (-1.0248 to -0.7694)** | **0** |
| **Central Sub-Saharan Africa** | **1999-2006** | **-1.4713 (-1.7231 to -1.2189)** | **0** |  |  |
| **Central Sub-Saharan Africa** | **2006-2012** | **-0.5246 (-0.8584 to -0.1897)** | **0.004091** |  |  |
| **Central Sub-Saharan Africa** | **2012-2017** | **-1.2519 (-1.7257 to -0.7757)** | **3.20E-05** |  |  |
| **Central Sub-Saharan Africa** | **2017-2021** | **-0.6372 (-1.1147 to -0.1574)** | **0.01215** |  |  |
| **Eastern Sub-Saharan Africa** | **1990-1995** | **-0.5676 (-0.736 to -0.399)** | **1.00E-06** | **-1.6333 (-1.7188 to -1.5477)** | **0** |
| **Eastern Sub-Saharan Africa** | **1995-1998** | **-1.1462 (-1.8931 to -0.3937)** | **0.005033** |  |  |
| **Eastern Sub-Saharan Africa** | **1998-2009** | **-2.4282 (-2.4865 to -2.3698)** | **0** |  |  |
| **Eastern Sub-Saharan Africa** | **2009-2014** | **-2.1208 (-2.3595 to -1.8816)** | **0** |  |  |
| **Eastern Sub-Saharan Africa** | **2014-2021** | **-0.9932 (-1.0959 to -0.8904)** | **0** |  |  |
| **North Africa and Middle East** | **1990-1996** | **-1.597 (-1.7747 to -1.419)** | **0** | **-1.6311 (-1.7867 to -1.4752)** | **0** |
| **North Africa and Middle East** | **1996-2001** | **-2.621 (-2.9511 to -2.2898)** | **0** |  |  |
| **North Africa and Middle East** | **2001-2009** | **-1.1717 (-1.3145 to -1.0288)** | **0** |  |  |
| **North Africa and Middle East** | **2009-2012** | **-1.6547 (-2.7383 to -0.559)** | **0.005844** |  |  |
| **North Africa and Middle East** | **2012-2015** | **-0.6119 (-1.6948 to 0.4829)** | **0.250973** |  |  |
| **North Africa and Middle East** | **2015-2021** | **-1.9418 (-2.1236 to -1.7596)** | **0** |  |  |
| **Southern Sub-Saharan Africa** | **1990-1995** | **-0.2269 (-1.0322 to 0.5849)** | **0.566092** | **-0.6852 (-1.0656 to -0.3032)** | **4.47E-04** |
| **Southern Sub-Saharan Africa** | **1995-1998** | **5.024 (1.2703 to 8.9168)** | **0.010708** |  |  |
| **Southern Sub-Saharan Africa** | **1998-2005** | **-0.0564 (-0.6716 to 0.5625)** | **0.851027** |  |  |
| **Southern Sub-Saharan Africa** | **2005-2021** | **-2.1324 (-2.2702 to -1.9945)** | **0** |  |  |
| **Western Sub-Saharan Africa** | **1990-2000** | **-0.9503 (-0.9894 to -0.9111)** | **0** | **-0.514 (-0.5738 to -0.4541)** | **0** |
| **Western Sub-Saharan Africa** | **2000-2010** | **0.3224 (0.2763 to 0.3684)** | **0** |  |  |
| **Western Sub-Saharan Africa** | **2010-2013** | **0.1072 (-0.3918 to 0.6087)** | **0.657849** |  |  |
| **Western Sub-Saharan Africa** | **2013-2017** | **-1.0438 (-1.2889 to -0.798)** | **0** |  |  |
| **Western Sub-Saharan Africa** | **2017-2021** | **-1.4318 (-1.5864 to -1.2769)** | **0** |  |  |
| **Andean Latin America** | **1990-1997** | **-0.0196 (-0.6309 to 0.5954)** | **0.94719** | **-1.3853 (-1.9662 to -0.801)** | **4.00E-06** |
| **Andean Latin America** | **1997-2012** | **-1.9379 (-2.1505 to -1.7247)** | **0** |  |  |
| **Andean Latin America** | **2012-2015** | **-4.6822 (-8.9539 to -0.21)** | **0.041334** |  |  |
| **Andean Latin America** | **2015-2019** | **2.0667 (-0.2147 to 4.4004)** | **0.073417** |  |  |
| **Andean Latin America** | **2019-2021** | **-3.7057 (-8.0535 to 0.8476)** | **0.103113** |  |  |
| **Caribbean** | **1990-1992** | **-3.4123 (-5.9183 to -0.8396)** | **0.011785** | **-1.5146 (-1.6914 to -1.3374)** | **0** |
| **Caribbean** | **1992-2010** | **-1.6707 (-1.7624 to -1.579)** | **0** |  |  |
| **Caribbean** | **2010-2021** | **-0.9086 (-1.0875 to -0.7294)** | **0** |  |  |
| **Central Latin America** | **1990-1994** | **-0.5176 (-1.2476 to 0.2177)** | **0.15603** | **-1.9224 (-2.1364 to -1.7079)** | **0** |
| **Central Latin America** | **1994-1998** | **-2.8536 (-3.9737 to -1.7205)** | **5.50E-05** |  |  |
| **Central Latin America** | **1998-2003** | **-1.8848 (-2.6047 to -1.1596)** | **3.70E-05** |  |  |
| **Central Latin America** | **2003-2014** | **-2.6476 (-2.8246 to -2.4703)** | **0** |  |  |
| **Central Latin America** | **2014-2021** | **-1.0639 (-1.3766 to -0.7502)** | **1.00E-06** |  |  |
| **High-income North America** | **1990-2003** | **-1.6321 (-1.7504 to -1.5136)** | **0** | **-1.8197 (-2.0336 to -1.6054)** | **0** |
| **High-income North America** | **2003-2006** | **-2.9762 (-5.1492 to -0.7534)** | **0.011071** |  |  |
| **High-income North America** | **2006-2021** | **-1.7495 (-1.8432 to -1.6558)** | **0** |  |  |
| **Southern Latin America** | **1990-1995** | **-1.8811 (-2.3201 to -1.4401)** | **0** | **-1.8395 (-2.1038 to -1.5744)** | **0** |
| **Southern Latin America** | **1995-1998** | **1.2047 (-0.8012 to 3.2512)** | **0.221702** |  |  |
| **Southern Latin America** | **1998-2008** | **-2.1366 (-2.316 to -1.9568)** | **0** |  |  |
| **Southern Latin America** | **2008-2012** | **-1.3232 (-2.3165 to -0.3198)** | **0.01329** |  |  |
| **Southern Latin America** | **2012-2019** | **-2.0613 (-2.3952 to -1.7263)** | **0** |  |  |
| **Southern Latin America** | **2019-2021** | **-4.947 (-6.8348 to -3.021)** | **7.50E-05** |  |  |
| **Tropical Latin America** | **1990-1993** | **-1.0157 (-2.1846 to 0.1672)** | **0.087762** | **-2.2263 (-2.4935 to -1.9584)** | **0** |
| **Tropical Latin America** | **1993-1996** | **-3.0324 (-5.297 to -0.7136)** | **0.013526** |  |  |
| **Tropical Latin America** | **1996-2004** | **-2.0413 (-2.3507 to -1.7309)** | **0** |  |  |
| **Tropical Latin America** | **2004-2010** | **-3.1213 (-3.6339 to -2.6059)** | **0** |  |  |
| **Tropical Latin America** | **2010-2021** | **-1.9775 (-2.136 to -1.8188)** | **0** |  |  |
| **Australasia** | **1990-2007** | **-2.6297 (-2.7636 to -2.4956)** | **0** | **-2.0776 (-2.1834 to -1.9717)** | **0** |
| **Australasia** | **2007-2021** | **-1.4029 (-1.5867 to -1.2188)** | **0** |  |  |
| **Central Asia** | **1990-1999** | **-3.5013 (-3.8193 to -3.1823)** | **0** | **-2.7864 (-2.9473 to -2.6252)** | **0** |
| **Central Asia** | **1999-2015** | **-2.0616 (-2.2139 to -1.909)** | **0** |  |  |
| **Central Asia** | **2015-2021** | **-3.632 (-4.2358 to -3.0244)** | **0** |  |  |
| **East Asia** | **1990-1998** | **-2.2707 (-2.3828 to -2.1585)** | **0** | **-2.4286 (-2.5781 to -2.279)** | **0** |
| **East Asia** | **1998-2004** | **0.3232 (0.0879 to 0.5592)** | **0.010381** |  |  |
| **East Asia** | **2004-2007** | **-6.1003 (-7.0802 to -5.11)** | **0** |  |  |
| **East Asia** | **2007-2010** | **-2.7704 (-3.793 to -1.7369)** | **4.50E-05** |  |  |
| **East Asia** | **2010-2015** | **-4.1075 (-4.4254 to -3.7886)** | **0** |  |  |
| **East Asia** | **2015-2021** | **-1.8978 (-2.0723 to -1.723)** | **0** |  |  |
| **High-income Asia Pacific** | **1990-1999** | **-3.2655 (-3.3759 to -3.155)** | **0** | **-3.3317 (-3.4767 to -3.1866)** | **0** |
| **High-income Asia Pacific** | **1999-2002** | **-4.5678 (-5.7544 to -3.3663)** | **0** |  |  |
| **High-income Asia Pacific** | **2002-2011** | **-3.0854 (-3.218 to -2.9525)** | **0** |  |  |
| **High-income Asia Pacific** | **2011-2019** | **-3.7484 (-3.9102 to -3.5863)** | **0** |  |  |
| **High-income Asia Pacific** | **2019-2021** | **-1.1769 (-2.417 to 0.0789)** | **0.064457** |  |  |
| **Oceania** | **1990-2005** | **-1.1276 (-1.1879 to -1.0672)** | **0** | **-0.8363 (-0.8752 to -0.7975)** | **0** |
| **Oceania** | **2005-2021** | **-0.5625 (-0.6174 to -0.5076)** | **0** |  |  |
| **South Asia** | **1990-1998** | **-0.8291 (-1.2207 to -0.436)** | **2.64E-04** | **-1.0138 (-1.3748 to -0.6515)** | **0** |
| **South Asia** | **1998-2001** | **-3.1038 (-6.5142 to 0.431)** | **0.081485** |  |  |
| **South Asia** | **2001-2014** | **-0.395 (-0.6074 to -0.1821)** | **9.19E-04** |  |  |
| **South Asia** | **2014-2021** | **-1.464 (-1.9325 to -0.9933)** | **2.00E-06** |  |  |
| **Southeast Asia** | **1990-1994** | **-0.8345 (-1.166 to -0.5018)** | **3.70E-05** | **-1.515 (-1.584 to -1.4459)** | **0** |
| **Southeast Asia** | **1994-2000** | **-1.343 (-1.5733 to -1.1121)** | **0** |  |  |
| **Southeast Asia** | **2000-2014** | **-2.0933 (-2.1466 to -2.0399)** | **0** |  |  |
| **Southeast Asia** | **2014-2021** | **-0.8878 (-1.027 to -0.7483)** | **0** |  |  |
| **Central Europe** | **1990-2003** | **-1.9484 (-2.0406 to -1.8562)** | **0** | **-2.4669 (-2.5433 to -2.3904)** | **0** |
| **Central Europe** | **2003-2013** | **-3.2027 (-3.3604 to -3.0447)** | **0** |  |  |
| **Central Europe** | **2013-2021** | **-2.3838 (-2.5725 to -2.1947)** | **0** |  |  |
| **Eastern Europe** | **1990-1994** | **-0.3639 (-1.5775 to 0.8646)** | **0.5398** | **-2.8255 (-3.3432 to -2.3051)** | **0** |
| **Eastern Europe** | **1994-1997** | **-5.6883 (-9.2857 to -1.9482)** | **0.005374** |  |  |
| **Eastern Europe** | **1997-2000** | **-0.9635 (-4.7259 to 2.9475)** | **0.605857** |  |  |
| **Eastern Europe** | **2000-2013** | **-3.6201 (-3.8391 to -3.4006)** | **0** |  |  |
| **Eastern Europe** | **2013-2021** | **-2.3452 (-2.7519 to -1.9369)** | **0** |  |  |
| **Western Europe** | **1990-1996** | **-3.3007 (-3.4895 to -3.1115)** | **0** | **-2.9902 (-3.114 to -2.8663)** | **0** |
| **Western Europe** | **1996-2000** | **-3.7973 (-4.3535 to -3.2378)** | **0** |  |  |
| **Western Europe** | **2000-2007** | **-3.3652 (-3.5541 to -3.1759)** | **0** |  |  |
| **Western Europe** | **2007-2014** | **-2.4491 (-2.638 to -2.2599)** | **0** |  |  |
| **Western Europe** | **2014-2018** | **-1.9795 (-2.5339 to -1.4219)** | **2.00E-06** |  |  |
| **Western Europe** | **2018-2021** | **-3.011 (-3.5601 to -2.4588)** | **0** |  |  |

**Supplemental Table5. The APC and AAPC in ASR of Gastric cancer due to high sodium diet DALYs for both sexes from 1990 to 2021.**

| **Location** | **Segment** | **APC** | **P Value** | **AAPC (1990-2021)** | **P Value** |
| --- | --- | --- | --- | --- | --- |
| **Global** | **1990-1998** | **-2.3779 (-2.4833 to -2.2724)** | **0** | **-2.4494 (-2.5567 to -2.3421)** | **0** |
| **Global** | **1998-2004** | **-1.4769 (-1.6961 to -1.2573)** | **0** |  |  |
| **Global** | **2004-2007** | **-4.1546 (-5.0929 to -3.207)** | **0** |  |  |
| **Global** | **2007-2015** | **-3.0334 (-3.162 to -2.9046)** | **0** |  |  |
| **Global** | **2015-2021** | **-1.8697 (-2.0326 to -1.7064)** | **0** |  |  |
| **High-middle SDI** | **1990-1994** | **-2.0858 (-2.4986 to -1.6713)** | **0** | **-2.594 (-2.7785 to -2.4093)** | **0** |
| **High-middle SDI** | **1994-1997** | **-3.4802 (-4.7578 to -2.1855)** | **4.50E-05** |  |  |
| **High-middle SDI** | **1997-2004** | **-0.9983 (-1.2203 to -0.7757)** | **0** |  |  |
| **High-middle SDI** | **2004-2007** | **-5.2186 (-6.4627 to -3.958)** | **0** |  |  |
| **High-middle SDI** | **2007-2015** | **-3.4952 (-3.6662 to -3.3238)** | **0** |  |  |
| **High-middle SDI** | **2015-2021** | **-1.7929 (-2.0111 to -1.5743)** | **0** |  |  |
| **High SDI** | **1990-1998** | **-2.9293 (-3.0592 to -2.7993)** | **0** | **-3.0259 (-3.1075 to -2.9443)** | **0** |
| **High SDI** | **1998-2019** | **-3.173 (-3.2054 to -3.1407)** | **0** |  |  |
| **High SDI** | **2019-2021** | **-1.8593 (-3.0442 to -0.6599)** | **0.003945** |  |  |
| **Low-middle SDI** | **1990-1998** | **-0.932 (-1.0753 to -0.7885)** | **0** | **-1.1779 (-1.3351 to -1.0204)** | **0** |
| **Low-middle SDI** | **1998-2005** | **-1.2574 (-1.4789 to -1.0355)** | **0** |  |  |
| **Low-middle SDI** | **2005-2008** | **-0.4034 (-1.7205 to 0.9313)** | **0.53153** |  |  |
| **Low-middle SDI** | **2008-2019** | **-1.3343 (-1.4361 to -1.2324)** | **0** |  |  |
| **Low-middle SDI** | **2019-2021** | **-2.1733 (-3.4535 to -0.876)** | **0.002533** |  |  |
| **Low SDI** | **1990-1997** | **-0.6975 (-0.8527 to -0.5421)** | **0** | **-1.3407 (-1.4102 to -1.2711)** | **0** |
| **Low SDI** | **1997-2005** | **-2.0604 (-2.2152 to -1.9054)** | **0** |  |  |
| **Low SDI** | **2005-2015** | **-1.0934 (-1.1991 to -0.9875)** | **0** |  |  |
| **Low SDI** | **2015-2021** | **-1.5369 (-1.7329 to -1.3405)** | **0** |  |  |
| **Middle SDI** | **1990-1999** | **-2.4377 (-2.534 to -2.3413)** | **0** | **-2.6475 (-2.8023 to -2.4925)** | **0** |
| **Middle SDI** | **1999-2004** | **-1.1027 (-1.4389 to -0.7654)** | **5.00E-06** |  |  |
| **Middle SDI** | **2004-2007** | **-5.2819 (-6.2976 to -4.2553)** | **0** |  |  |
| **Middle SDI** | **2007-2010** | **-2.8913 (-3.9337 to -1.8376)** | **3.50E-05** |  |  |
| **Middle SDI** | **2010-2015** | **-3.6596 (-3.9891 to -3.329)** | **0** |  |  |
| **Middle SDI** | **2015-2021** | **-1.9312 (-2.1105 to -1.7515)** | **0** |  |  |
| **Central Sub-Saharan Africa** | **1990-1999** | **-0.639 (-0.7983 to -0.4794)** | **0** | **-0.9842 (-1.1043 to -0.864)** | **0** |
| **Central Sub-Saharan Africa** | **1999-2006** | **-1.5395 (-1.8287 to -1.2495)** | **0** |  |  |
| **Central Sub-Saharan Africa** | **2006-2011** | **-0.537 (-1.0873 to 0.0164)** | **0.056529** |  |  |
| **Central Sub-Saharan Africa** | **2011-2021** | **-1.1276 (-1.2641 to -0.9909)** | **0** |  |  |
| **Eastern Sub-Saharan Africa** | **1990-1995** | **-0.6463 (-0.8327 to -0.4596)** | **1.00E-06** | **-1.8991 (-1.994 to -1.8041)** | **0** |
| **Eastern Sub-Saharan Africa** | **1995-1998** | **-1.4409 (-2.2689 to -0.6058)** | **0.001984** |  |  |
| **Eastern Sub-Saharan Africa** | **1998-2009** | **-2.8275 (-2.8922 to -2.7627)** | **0** |  |  |
| **Eastern Sub-Saharan Africa** | **2009-2014** | **-2.4092 (-2.6755 to -2.1421)** | **0** |  |  |
| **Eastern Sub-Saharan Africa** | **2014-2021** | **-1.1502 (-1.2647 to -1.0355)** | **0** |  |  |
| **North Africa and Middle East** | **1990-1996** | **-1.772 (-1.971 to -1.5726)** | **0** | **-1.8605 (-2.0331 to -1.6876)** | **0** |
| **North Africa and Middle East** | **1996-2001** | **-3.0017 (-3.3709 to -2.6311)** | **0** |  |  |
| **North Africa and Middle East** | **2001-2004** | **-1.1853 (-2.3812 to 0.0253)** | **0.054359** |  |  |
| **North Africa and Middle East** | **2004-2012** | **-1.6454 (-1.8056 to -1.4848)** | **0** |  |  |
| **North Africa and Middle East** | **2012-2015** | **-1.119 (-2.3236 to 0.1006)** | **0.069269** |  |  |
| **North Africa and Middle East** | **2015-2021** | **-1.9847 (-2.187 to -1.7819)** | **0** |  |  |
| **Southern Sub-Saharan Africa** | **1990-1995** | **-0.9112 (-1.9083 to 0.0962)** | **0.073773** | **-0.8504 (-1.3243 to -0.3741)** | **4.78E-04** |
| **Southern Sub-Saharan Africa** | **1995-1998** | **4.2867 (-0.3471 to 9.136)** | **0.068476** |  |  |
| **Southern Sub-Saharan Africa** | **1998-2005** | **0.5377 (-0.2338 to 1.3152)** | **0.162439** |  |  |
| **Southern Sub-Saharan Africa** | **2005-2021** | **-2.3619 (-2.5328 to -2.1907)** | **0** |  |  |
| **Western Sub-Saharan Africa** | **1990-2000** | **-1.0363 (-1.087 to -0.9855)** | **0** | **-0.686 (-0.7141 to -0.6578)** | **0** |
| **Western Sub-Saharan Africa** | **2000-2014** | **0.004 (-0.03 to 0.038)** | **0.809341** |  |  |
| **Western Sub-Saharan Africa** | **2014-2021** | **-1.5561 (-1.6412 to -1.4709)** | **0** |  |  |
| **Andean Latin America** | **1990-1996** | **-0.2773 (-1.0178 to 0.4687)** | **0.443883** | **-1.5036 (-1.9811 to -1.0237)** | **0** |
| **Andean Latin America** | **1996-2011** | **-1.9581 (-2.162 to -1.7538)** | **0** |  |  |
| **Andean Latin America** | **2011-2015** | **-4.2591 (-6.3568 to -2.1144)** | **6.32E-04** |  |  |
| **Andean Latin America** | **2015-2019** | **2.0418 (-0.1507 to 4.2824)** | **0.066291** |  |  |
| **Andean Latin America** | **2019-2021** | **-3.106 (-7.2653 to 1.2398)** | **0.148175** |  |  |
| **Caribbean** | **1990-1996** | **-2.1608 (-2.5671 to -1.7528)** | **0** | **-1.3242 (-1.7161 to -0.9308)** | **0** |
| **Caribbean** | **1996-1999** | **-0.5638 (-2.9844 to 1.9172)** | **0.631911** |  |  |
| **Caribbean** | **1999-2002** | **-2.701 (-5.0666 to -0.2764)** | **0.031553** |  |  |
| **Caribbean** | **2002-2012** | **-1.3454 (-1.5679 to -1.1224)** | **0** |  |  |
| **Caribbean** | **2012-2015** | **0.3161 (-2.0892 to 2.7804)** | **0.785448** |  |  |
| **Caribbean** | **2015-2021** | **-0.947 (-1.3647 to -0.5276)** | **2.34E-04** |  |  |
| **Central Latin America** | **1990-1994** | **-0.4865 (-1.2036 to 0.2358)** | **0.173642** | **-1.7437 (-1.9533 to -1.5336)** | **0** |
| **Central Latin America** | **1994-1998** | **-2.7275 (-3.825 to -1.6174)** | **7.20E-05** |  |  |
| **Central Latin America** | **1998-2003** | **-1.7124 (-2.4145 to -1.0052)** | **8.10E-05** |  |  |
| **Central Latin America** | **2003-2014** | **-2.4736 (-2.6472 to -2.2998)** | **0** |  |  |
| **Central Latin America** | **2014-2021** | **-0.7585 (-1.0661 to -0.45)** | **6.70E-05** |  |  |
| **High-income North America** | **1990-2003** | **-1.621 (-1.7494 to -1.4925)** | **0** | **-1.681 (-1.9123 to -1.4491)** | **0** |
| **High-income North America** | **2003-2006** | **-2.6496 (-5.0023 to -0.2386)** | **0.032791** |  |  |
| **High-income North America** | **2006-2021** | **-1.5382 (-1.6396 to -1.4367)** | **0** |  |  |
| **Southern Latin America** | **1990-1995** | **-1.8391 (-2.2946 to -1.3813)** | **0** | **-1.8633 (-2.099 to -1.627)** | **0** |
| **Southern Latin America** | **1995-1998** | **0.9232 (-1.1469 to 3.0367)** | **0.366943** |  |  |
| **Southern Latin America** | **1998-2019** | **-1.9583 (-2.0141 to -1.9025)** | **0** |  |  |
| **Southern Latin America** | **2019-2021** | **-4.9958 (-6.9375 to -3.0135)** | **4.10E-05** |  |  |
| **Tropical Latin America** | **1990-1993** | **-1.0491 (-2.107 to 0.0202)** | **0.053911** | **-2.1408 (-2.4064 to -1.8745)** | **0** |
| **Tropical Latin America** | **1993-1996** | **-2.8024 (-4.8635 to -0.6966)** | **0.012756** |  |  |
| **Tropical Latin America** | **1996-2004** | **-2.0184 (-2.2995 to -1.7365)** | **0** |  |  |
| **Tropical Latin America** | **2004-2009** | **-3.1229 (-3.779 to -2.4624)** | **0** |  |  |
| **Tropical Latin America** | **2009-2018** | **-2.209 (-2.4405 to -1.9771)** | **0** |  |  |
| **Tropical Latin America** | **2018-2021** | **-1.0328 (-2.1121 to 0.0584)** | **0.061843** |  |  |
| **Australasia** | **1990-2006** | **-2.6614 (-2.8001 to -2.5225)** | **0** | **-2.151 (-2.2501 to -2.0517)** | **0** |
| **Australasia** | **2006-2021** | **-1.6035 (-1.7589 to -1.4479)** | **0** |  |  |
| **Central Asia** | **1990-1999** | **-3.4799 (-3.776 to -3.1828)** | **0** | **-2.9766 (-3.1266 to -2.8265)** | **0** |
| **Central Asia** | **1999-2015** | **-2.4734 (-2.6144 to -2.3322)** | **0** |  |  |
| **Central Asia** | **2015-2021** | **-3.5565 (-4.1227 to -2.987)** | **0** |  |  |
| **East Asia** | **1990-1998** | **-2.5728 (-2.6823 to -2.4632)** | **0** | **-2.7255 (-2.8714 to -2.5794)** | **0** |
| **East Asia** | **1998-2004** | **-0.373 (-0.6019 to -0.1436)** | **0.003474** |  |  |
| **East Asia** | **2004-2007** | **-6.1285 (-7.0886 to -5.1585)** | **0** |  |  |
| **East Asia** | **2007-2010** | **-3.4261 (-4.4193 to -2.4226)** | **3.00E-06** |  |  |
| **East Asia** | **2010-2015** | **-4.2026 (-4.5135 to -3.8906)** | **0** |  |  |
| **East Asia** | **2015-2021** | **-1.9263 (-2.0997 to -1.7526)** | **0** |  |  |
| **High-income Asia Pacific** | **1990-1999** | **-3.731 (-3.835 to -3.6269)** | **0** | **-3.8056 (-3.9419 to -3.669)** | **0** |
| **High-income Asia Pacific** | **1999-2002** | **-4.9904 (-6.1059 to -3.8616)** | **0** |  |  |
| **High-income Asia Pacific** | **2002-2011** | **-3.5034 (-3.6282 to -3.3785)** | **0** |  |  |
| **High-income Asia Pacific** | **2011-2019** | **-4.4158 (-4.5675 to -4.264)** | **0** |  |  |
| **High-income Asia Pacific** | **2019-2021** | **-1.2274 (-2.4035 to -0.037)** | **0.043996** |  |  |
| **Oceania** | **1990-2005** | **-1.1702 (-1.244 to -1.0962)** | **0** | **-0.8287 (-0.876 to -0.7813)** | **0** |
| **Oceania** | **2005-2021** | **-0.5074 (-0.5738 to -0.441)** | **0** |  |  |
| **South Asia** | **1990-1998** | **-1.0904 (-1.3825 to -0.7974)** | **0** | **-1.2064 (-1.3854 to -1.0271)** | **0** |
| **South Asia** | **1998-2004** | **-2.2632 (-2.8522 to -1.6707)** | **0** |  |  |
| **South Asia** | **2004-2010** | **-0.1176 (-0.7132 to 0.4816)** | **0.686682** |  |  |
| **South Asia** | **2010-2021** | **-1.3019 (-1.4799 to -1.1236)** | **0** |  |  |
| **Southeast Asia** | **1990-1994** | **-1.0234 (-1.3996 to -0.6458)** | **2.20E-05** | **-1.6556 (-1.7511 to -1.56)** | **0** |
| **Southeast Asia** | **1994-2002** | **-1.5529 (-1.7096 to -1.3959)** | **0** |  |  |
| **Southeast Asia** | **2002-2008** | **-2.5128 (-2.7737 to -2.2512)** | **0** |  |  |
| **Southeast Asia** | **2008-2014** | **-2.0517 (-2.3154 to -1.7871)** | **0** |  |  |
| **Southeast Asia** | **2014-2021** | **-1.0531 (-1.2124 to -0.8936)** | **0** |  |  |
| **Central Europe** | **1990-2003** | **-2.0295 (-2.1227 to -1.9361)** | **0** | **-2.5337 (-2.6819 to -2.3853)** | **0** |
| **Central Europe** | **2003-2008** | **-2.8683 (-3.4249 to -2.3086)** | **0** |  |  |
| **Central Europe** | **2008-2012** | **-3.6874 (-4.555 to -2.8118)** | **0** |  |  |
| **Central Europe** | **2012-2021** | **-2.5581 (-2.7173 to -2.3987)** | **0** |  |  |
| **Eastern Europe** | **1990-1994** | **0.0091 (-1.3594 to 1.3966)** | **0.989091** | **-3.0491 (-3.557 to -2.5385)** | **0** |
| **Eastern Europe** | **1994-1997** | **-5.9219 (-9.9415 to -1.723)** | **0.008808** |  |  |
| **Eastern Europe** | **1997-2001** | **-1.7508 (-3.8729 to 0.4182)** | **0.10646** |  |  |
| **Eastern Europe** | **2001-2013** | **-4.0717 (-4.3513 to -3.7914)** | **0** |  |  |
| **Eastern Europe** | **2013-2021** | **-2.5625 (-3.0184 to -2.1044)** | **0** |  |  |
| **Western Europe** | **1990-2007** | **-3.3832 (-3.4381 to -3.3283)** | **0** | **-2.9998 (-3.0868 to -2.9127)** | **0** |
| **Western Europe** | **2007-2018** | **-2.3267 (-2.4489 to -2.2043)** | **0** |  |  |
| **Western Europe** | **2018-2021** | **-3.2815 (-4.0516 to -2.5052)** | **0** |  |  |

**Supplemental Table6. The APC and AAPC in ASR of Gastric cancer due to high sodium diet mortality for female from 1990 to 2021.**

| **Location** | **Segment** | **APC** | **P Value** | **AAPC (1990-2021)** | **P Value** |
| --- | --- | --- | --- | --- | --- |
| **Global** | **1990-1994** | **-2.1292 (-2.4057 to -1.8519)** | **0** | **-2.3851 (-2.4968 to -2.2733)** | **0** |
| **Global** | **1994-1998** | **-2.648 (-3.0807 to -2.2134)** | **0** |  |  |
| **Global** | **1998-2004** | **-1.6296 (-1.8262 to -1.4326)** | **0** |  |  |
| **Global** | **2004-2007** | **-4.4292 (-5.2763 to -3.5745)** | **0** |  |  |
| **Global** | **2007-2014** | **-3.0893 (-3.237 to -2.9413)** | **0** |  |  |
| **Global** | **2014-2021** | **-1.429 (-1.5487 to -1.3091)** | **0** |  |  |
| **High-middle SDI** | **1990-1998** | **-2.6343 (-2.8363 to -2.4319)** | **0** | **-2.5918 (-2.7987 to -2.3845)** | **0** |
| **High-middle SDI** | **1998-2004** | **-1.057 (-1.4785 to -0.6337)** | **5.70E-05** |  |  |
| **High-middle SDI** | **2004-2007** | **-5.186 (-6.9636 to -3.3744)** | **1.40E-05** |  |  |
| **High-middle SDI** | **2007-2014** | **-3.926 (-4.2366 to -3.6144)** | **0** |  |  |
| **High-middle SDI** | **2014-2021** | **-1.3708 (-1.6237 to -1.1173)** | **0** |  |  |
| **High SDI** | **1990-2015** | **-3.0035 (-3.0352 to -2.9719)** | **0** | **-2.8627 (-2.9191 to -2.8062)** | **0** |
| **High SDI** | **2015-2021** | **-2.2735 (-2.5501 to -1.996)** | **0** |  |  |
| **Low-middle SDI** | **1990-1998** | **-0.7472 (-0.8977 to -0.5966)** | **0** | **-1.0884 (-1.2665 to -0.91)** | **0** |
| **Low-middle SDI** | **1998-2001** | **-1.5404 (-2.8892 to -0.173)** | **0.029887** |  |  |
| **Low-middle SDI** | **2001-2007** | **-0.6682 (-0.9766 to -0.3589)** | **3.50E-04** |  |  |
| **Low-middle SDI** | **2007-2011** | **-1.402 (-2.0837 to -0.7156)** | **5.86E-04** |  |  |
| **Low-middle SDI** | **2011-2018** | **-0.7947 (-1.0277 to -0.561)** | **3.00E-06** |  |  |
| **Low-middle SDI** | **2018-2021** | **-2.6357 (-3.3149 to -1.9517)** | **1.00E-06** |  |  |
| **Low SDI** | **1990-1996** | **-0.5106 (-0.7071 to -0.3138)** | **3.60E-05** | **-1.0262 (-1.1661 to -0.886)** | **0** |
| **Low SDI** | **1996-2000** | **-1.8332 (-2.414 to -1.2489)** | **4.00E-06** |  |  |
| **Low SDI** | **2000-2011** | **-1.2086 (-1.301 to -1.1161)** | **0** |  |  |
| **Low SDI** | **2011-2014** | **0.7434 (-0.4496 to 1.9507)** | **0.207922** |  |  |
| **Low SDI** | **2014-2021** | **-1.4676 (-1.6244 to -1.3105)** | **0** |  |  |
| **Middle SDI** | **1990-2000** | **-2.5717 (-2.7159 to -2.4273)** | **0** | **-2.7575 (-3.0481 to -2.466)** | **0** |
| **Middle SDI** | **2000-2004** | **-1.3639 (-2.3009 to -0.4178)** | **0.007838** |  |  |
| **Middle SDI** | **2004-2007** | **-6.3034 (-8.0876 to -4.4847)** | **3.00E-06** |  |  |
| **Middle SDI** | **2007-2010** | **-2.8517 (-4.7089 to -0.9582)** | **0.00603** |  |  |
| **Middle SDI** | **2010-2014** | **-4.2394 (-5.1576 to -3.3123)** | **0** |  |  |
| **Middle SDI** | **2014-2021** | **-1.3616 (-1.6201 to -1.1023)** | **0** |  |  |

**Supplemental Table7. The APC and AAPC in ASR of Gastric cancer due to high sodium diet mortality for male from 1990 to 2021.**

| **Location** | **Segment** | **APC** | **P Value** | **AAPC (1990-2021)** | **P Value** |
| --- | --- | --- | --- | --- | --- |
| **Global** | **1990-1998** | **-2.0056 (-2.1093 to -1.9018)** | **0** | **-2.0721 (-2.2087 to -1.9354)** | **0** |
| **Global** | **1998-2004** | **-0.8022 (-1.0156 to -0.5884)** | **1.00E-06** |  |  |
| **Global** | **2004-2007** | **-3.9632 (-4.877 to -3.0406)** | **0** |  |  |
| **Global** | **2007-2010** | **-1.8916 (-2.829 to -0.9452)** | **7.13E-04** |  |  |
| **Global** | **2010-2015** | **-2.9247 (-3.2171 to -2.6314)** | **0** |  |  |
| **Global** | **2015-2021** | **-1.8441 (-2.0023 to -1.6857)** | **0** |  |  |
| **High-middle SDI** | **1990-1998** | **-2.4676 (-2.6521 to -2.2828)** | **0** | **-2.2601 (-2.4496 to -2.0702)** | **0** |
| **High-middle SDI** | **1998-2004** | **-0.2026 (-0.5849 to 0.1811)** | **0.281515** |  |  |
| **High-middle SDI** | **2004-2007** | **-4.9532 (-6.5768 to -3.3014)** | **8.00E-06** |  |  |
| **High-middle SDI** | **2007-2017** | **-2.8111 (-2.9646 to -2.6574)** | **0** |  |  |
| **High-middle SDI** | **2017-2021** | **-1.466 (-2.0037 to -0.9254)** | **2.20E-05** |  |  |
| **High SDI** | **1990-1998** | **-2.376 (-2.5025 to -2.2493)** | **0** | **-2.6588 (-2.7469 to -2.5706)** | **0** |
| **High SDI** | **1998-2010** | **-2.7766 (-2.8541 to -2.6991)** | **0** |  |  |
| **High SDI** | **2010-2019** | **-3.0369 (-3.1617 to -2.912)** | **0** |  |  |
| **High SDI** | **2019-2021** | **-1.3676 (-2.5212 to -0.2003)** | **0.023941** |  |  |
| **Low-middle SDI** | **1990-2005** | **-0.7539 (-0.8322 to -0.6755)** | **0** | **-0.8869 (-1.0668 to -0.7066)** | **0** |
| **Low-middle SDI** | **2005-2008** | **0.2626 (-1.6152 to 2.1763)** | **0.777082** |  |  |
| **Low-middle SDI** | **2008-2021** | **-1.3031 (-1.3996 to -1.2066)** | **0** |  |  |
| **Low SDI** | **1990-1997** | **-0.6342 (-0.905 to -0.3627)** | **8.60E-05** | **-1.1588 (-1.2793 to -1.0381)** | **0** |
| **Low SDI** | **1997-2005** | **-1.7794 (-2.0475 to -1.5106)** | **0** |  |  |
| **Low SDI** | **2005-2015** | **-0.6287 (-0.8127 to -0.4444)** | **1.00E-06** |  |  |
| **Low SDI** | **2015-2021** | **-1.818 (-2.1546 to -1.4802)** | **0** |  |  |
| **Middle SDI** | **1990-1999** | **-1.7512 (-1.8677 to -1.6345)** | **0** | **-2.0818 (-2.2684 to -1.8947)** | **0** |
| **Middle SDI** | **1999-2004** | **-0.0386 (-0.4479 to 0.3723)** | **0.84358** |  |  |
| **Middle SDI** | **2004-2007** | **-4.9007 (-6.114 to -3.6717)** | **1.00E-06** |  |  |
| **Middle SDI** | **2007-2010** | **-1.5875 (-2.8599 to -0.2984)** | **0.01928** |  |  |
| **Middle SDI** | **2010-2015** | **-3.323 (-3.7181 to -2.9262)** | **0** |  |  |
| **Middle SDI** | **2015-2021** | **-2.0373 (-2.254 to -1.8201)** | **0** |  |  |

**Supplemental Table8. The APC and AAPC in ASR of Gastric cancer due to high sodium diet DALYs for female from 1990 to 2021.**

| **Location** | **Segment** | **APC** | **P Value** | **AAPC (1990-2021)** | **P Value** |
| --- | --- | --- | --- | --- | --- |
| **Global** | **1990-1994** | **-2.2774 (-2.565 to -1.989)** | **0** | **-2.622 (-2.7511 to -2.4928)** | **0** |
| **Global** | **1994-1997** | **-3.1033 (-3.9961 to -2.2021)** | **3.00E-06** |  |  |
| **Global** | **1997-2004** | **-2.0083 (-2.1608 to -1.8556)** | **0** |  |  |
| **Global** | **2004-2007** | **-4.7034 (-5.5818 to -3.8167)** | **0** |  |  |
| **Global** | **2007-2014** | **-3.3717 (-3.524 to -3.2193)** | **0** |  |  |
| **Global** | **2014-2021** | **-1.5636 (-1.6872 to -1.44)** | **0** |  |  |
| **High-middle SDI** | **1990-1994** | **-2.2401 (-2.8741 to -1.6019)** | **2.00E-06** | **-2.8493 (-3.1313 to -2.5666)** | **0** |
| **High-middle SDI** | **1994-1997** | **-3.5388 (-5.481 to -1.5567)** | **0.001833** |  |  |
| **High-middle SDI** | **1997-2004** | **-1.4468 (-1.7851 to -1.1073)** | **0** |  |  |
| **High-middle SDI** | **2004-2007** | **-5.473 (-7.3617 to -3.5457)** | **2.70E-05** |  |  |
| **High-middle SDI** | **2007-2014** | **-4.3281 (-4.6585 to -3.9966)** | **0** |  |  |
| **High-middle SDI** | **2014-2021** | **-1.6551 (-1.9248 to -1.3847)** | **0** |  |  |
| **High SDI** | **1990-2015** | **-3.3631 (-3.3897 to -3.3365)** | **0** | **-3.2107 (-3.2581 to -3.1632)** | **0** |
| **High SDI** | **2015-2021** | **-2.5729 (-2.8056 to -2.3396)** | **0** |  |  |
| **Low-middle SDI** | **1990-1998** | **-1.0057 (-1.1576 to -0.8536)** | **0** | **-1.2547 (-1.3492 to -1.1601)** | **0** |
| **Low-middle SDI** | **1998-2011** | **-1.2855 (-1.3676 to -1.2033)** | **0** |  |  |
| **Low-middle SDI** | **2011-2018** | **-0.8409 (-1.0751 to -0.6062)** | **0** |  |  |
| **Low-middle SDI** | **2018-2021** | **-2.7366 (-3.4218 to -2.0465)** | **0** |  |  |
| **Low SDI** | **1990-1996** | **-0.6728 (-0.8343 to -0.5111)** | **0** | **-1.2641 (-1.3387 to -1.1894)** | **0** |
| **Low SDI** | **1996-2010** | **-1.7295 (-1.7802 to -1.6788)** | **0** |  |  |
| **Low SDI** | **2010-2014** | **-0.318 (-0.8078 to 0.1743)** | **0.193122** |  |  |
| **Low SDI** | **2014-2021** | **-1.3742 (-1.5022 to -1.246)** | **0** |  |  |
| **Middle SDI** | **1990-1995** | **-2.5819 (-2.8448 to -2.3183)** | **0** | **-3.0254 (-3.1918 to -2.8588)** | **0** |
| **Middle SDI** | **1995-1998** | **-3.6437 (-4.7838 to -2.49)** | **8.00E-06** |  |  |
| **Middle SDI** | **1998-2004** | **-2.068 (-2.3294 to -1.8058)** | **0** |  |  |
| **Middle SDI** | **2004-2007** | **-6.0842 (-7.206 to -4.9488)** | **0** |  |  |
| **Middle SDI** | **2007-2014** | **-4.0229 (-4.2183 to -3.827)** | **0** |  |  |
| **Middle SDI** | **2014-2021** | **-1.5465 (-1.708 to -1.3848)** | **0** |  |  |

**Supplemental Table9. The APC and AAPC in ASR of Gastric cancer due to high sodium diet DALYs for male from 1990 to 2021.**

| **Location** | **Segment** | **APC (95% CI)** | **P Value** | **AAPC (95% CI) (1990-2021)** | **P Value** |
| --- | --- | --- | --- | --- | --- |
| **Global** | **1990-1998** | **-2.2718 (-2.3835 to -2.1599)** | **0** | **-2.3565 (-2.5039 to -2.2088)** | **0** |
| **Global** | **1998-2004** | **-1.1894 (-1.4191 to -0.9591)** | **0** |  |  |
| **Global** | **2004-2007** | **-4.116 (-5.1063 to -3.1154)** | **0** |  |  |
| **Global** | **2007-2010** | **-2.4225 (-3.4289 to -1.4056)** | **1.46E-04** |  |  |
| **Global** | **2010-2015** | **-3.2012 (-3.5175 to -2.8839)** | **0** |  |  |
| **Global** | **2015-2021** | **-2.0009 (-2.1723 to -1.8292)** | **0** |  |  |
| **High-middle SDI** | **1990-1994** | **-2.1341 (-2.5843 to -1.6818)** | **0** | **-2.544 (-2.7455 to -2.342)** | **0** |
| **High-middle SDI** | **1994-1997** | **-3.5307 (-4.9165 to -2.1247)** | **9.00E-05** |  |  |
| **High-middle SDI** | **1997-2004** | **-0.8221 (-1.0637 to -0.58)** | **3.00E-06** |  |  |
| **High-middle SDI** | **2004-2007** | **-5.0557 (-6.4243 to -3.667)** | **2.00E-06** |  |  |
| **High-middle SDI** | **2007-2015** | **-3.295 (-3.4822 to -3.1074)** | **0** |  |  |
| **High-middle SDI** | **2015-2021** | **-2.0332 (-2.2736 to -1.7922)** | **0** |  |  |
| **High SDI** | **1990-1999** | **-2.7824 (-2.8616 to -2.7031)** | **0** | **-3.0336 (-3.1635 to -2.9035)** | **0** |
| **High SDI** | **1999-2002** | **-3.5205 (-4.3729 to -2.6604)** | **0** |  |  |
| **High SDI** | **2002-2011** | **-3.0282 (-3.1216 to -2.9347)** | **0** |  |  |
| **High SDI** | **2011-2014** | **-3.7586 (-4.606 to -2.9038)** | **0** |  |  |
| **High SDI** | **2014-2019** | **-3.1646 (-3.4326 to -2.8958)** | **0** |  |  |
| **High SDI** | **2019-2021** | **-2.0305 (-2.8867 to -1.1667)** | **1.64E-04** |  |  |
| **Low-middle SDI** | **1990-2005** | **-0.9498 (-1.0219 to -0.8777)** | **0** | **-1.0538 (-1.1974 to -0.91)** | **0** |
| **Low-middle SDI** | **2005-2010** | **-0.36 (-0.9023 to 0.1852)** | **0.183775** |  |  |
| **Low-middle SDI** | **2010-2014** | **-1.9108 (-2.7449 to -1.0694)** | **1.23E-04** |  |  |
| **Low-middle SDI** | **2014-2021** | **-1.2783 (-1.5015 to -1.0547)** | **0** |  |  |
| **Low SDI** | **1990-1998** | **-0.7638 (-0.9183 to -0.609)** | **0** | **-1.3443 (-1.4422 to -1.2463)** | **0** |
| **Low SDI** | **1998-2005** | **-2.255 (-2.4902 to -2.0191)** | **0** |  |  |
| **Low SDI** | **2005-2010** | **-0.6735 (-1.1226 to -0.2223)** | **0.005411** |  |  |
| **Low SDI** | **2010-2021** | **-1.4867 (-1.582 to -1.3914)** | **0** |  |  |
| **Middle SDI** | **1990-1999** | **-2.1395 (-2.2434 to -2.0355)** | **0** | **-2.4069 (-2.5737 to -2.2399)** | **0** |
| **Middle SDI** | **1999-2004** | **-0.5488 (-0.9129 to -0.1833)** | **0.005996** |  |  |
| **Middle SDI** | **2004-2007** | **-4.8365 (-5.9298 to -3.7305)** | **0** |  |  |
| **Middle SDI** | **2007-2010** | **-2.4021 (-3.5302 to -1.2608)** | **4.61E-04** |  |  |
| **Middle SDI** | **2010-2015** | **-3.5337 (-3.887 to -3.1791)** | **0** |  |  |
| **Middle SDI** | **2015-2021** | **-2.169 (-2.363 to -1.9747)** | **0** |  |  |

**Supplemental Table10. RRs of Gastric cancer (due to high sodium diet) Mortality and DALYs for female and male due to age, period, and birth cohort effects.**

| **Mortality** | | | | | | | **DALYs** | | | | | |
| --- | --- | --- | --- | --- | --- | --- | --- | --- | --- | --- | --- | --- |
|  | **Both** | | **Female** | | **Male** | | **Both** | | **Female** | | **Male** | |
| **variable** | **RR (95% CI)** | **P** | **RR (95% CI)** | **P** | **RR (95% CI)** | **P** | **RR (95% CI)** | **P** | **RR (95% CI)** | **P** | **RR (95% CI)** | **P** |
| **Age (years)** | | | | | | | | | | | | |
| **25-29** | **0.066 (0.063 to 0.069)** | **<0.001** | **0.093 (0.087 to 0.099)** | **<0.001** | **0.05 (0.046 to 0.053)** | **<0.001** | **0.12 (0.119 to 0.121)** | **<0.001** | **0.168 (0.166 to 0.17)** | **<0.001** | **0.09 (0.088 to 0.091)** | **<0.001** |
| **30-34** | **0.136 (0.132 to 0.14)** | **<0.001** | **0.167 (0.159 to 0.174)** | **<0.001** | **0.118 (0.113 to 0.123)** | **<0.001** | **0.239 (0.238 to 0.24)** | **<0.001** | **0.29 (0.287 to 0.293)** | **<0.001** | **0.205 (0.203 to 0.207)** | **<0.001** |
| **35-39** | **0.236 (0.23 to 0.242)** | **<0.001** | **0.261 (0.252 to 0.271)** | **<0.001** | **0.219 (0.212 to 0.226)** | **<0.001** | **0.396 (0.395 to 0.398)** | **<0.001** | **0.435 (0.431 to 0.438)** | **<0.001** | **0.365 (0.361 to 0.368)** | **<0.001** |
| **40-44** | **0.382 (0.375 to 0.39)** | **<0.001** | **0.375 (0.364 to 0.388)** | **<0.001** | **0.38 (0.37 to 0.39)** | **<0.001** | **0.61 (0.608 to 0.612)** | **<0.001** | **0.593 (0.589 to 0.597)** | **<0.001** | **0.6 (0.595 to 0.604)** | **<0.001** |
| **45-49** | **0.548 (0.539 to 0.557)** | **<0.001** | **0.476 (0.463 to 0.49)** | **<0.001** | **0.575 (0.563 to 0.588)** | **<0.001** | **0.821 (0.818 to 0.823)** | **<0.001** | **0.707 (0.702 to 0.711)** | **<0.001** | **0.852 (0.847 to 0.857)** | **<0.001** |
| **50-54** | **0.837 (0.825 to 0.849)** | **<0.001** | **0.691 (0.674 to 0.709)** | **<0.001** | **0.898 (0.881 to 0.914)** | **<0.001** | **1.166 (1.164 to 1.169)** | **<0.001** | **0.955 (0.949 to 0.96)** | **<0.001** | **1.238 (1.232 to 1.244)** | **<0.001** |
| **55-59** | **1.154 (1.139 to 1.168)** | **<0.001** | **0.919 (0.899 to 0.94)** | **<0.001** | **1.258 (1.238 to 1.278)** | **<0.001** | **1.477 (1.473 to 1.48)** | **<0.001** | **1.167 (1.16 to 1.173)** | **<0.001** | **1.594 (1.588 to 1.601)** | **<0.001** |
| **60-64** | **1.516 (1.498 to 1.533)** | **<0.001** | **1.231 (1.207 to 1.255)** | **<0.001** | **1.648 (1.625 to 1.672)** | **<0.001** | **1.749 (1.745 to 1.753)** | **<0.001** | **1.41 (1.403 to 1.417)** | **<0.001** | **1.887 (1.879 to 1.895)** | **<0.001** |
| **65-69** | **1.904 (1.884 to 1.925)** | **<0.001** | **1.598 (1.569 to 1.627)** | **<0.001** | **2.061 (2.032 to 2.09)** | **<0.001** | **1.934 (1.93 to 1.939)** | **<0.001** | **1.614 (1.605 to 1.622)** | **<0.001** | **2.081 (2.072 to 2.091)** | **<0.001** |
| **70-74** | **2.385 (2.36 to 2.411)** | **<0.001** | **2.076 (2.041 to 2.113)** | **<0.001** | **2.577 (2.539 to 2.615)** | **<0.001** | **2.085 (2.079 to 2.09)** | **<0.001** | **1.81 (1.799 to 1.82)** | **<0.001** | **2.247 (2.234 to 2.26)** | **<0.001** |
| **75-79** | **2.718 (2.686 to 2.75)** | **<0.001** | **2.562 (2.517 to 2.607)** | **<0.001** | **2.886 (2.838 to 2.936)** | **<0.001** | **1.987 (1.98 to 1.993)** | **<0.001** | **1.875 (1.863 to 1.887)** | **<0.001** | **2.118 (2.103 to 2.133)** | **<0.001** |
| **80-84** | **2.977 (2.937 to 3.017)** | **<0.001** | **3.008 (2.95 to 3.067)** | **<0.001** | **3.142 (3.079 to 3.206)** | **<0.001** | **1.781 (1.774 to 1.789)** | **<0.001** | **1.816 (1.802 to 1.83)** | **<0.001** | **1.901 (1.885 to 1.918)** | **<0.001** |
| **85-89** | **3.34 (3.286 to 3.395)** | **<0.001** | **3.488 (3.409 to 3.568)** | **<0.001** | **3.626 (3.538 to 3.717)** | **<0.001** | **1.658 (1.649 to 1.666)** | **<0.001** | **1.764 (1.747 to 1.781)** | **<0.001** | **1.836 (1.816 to 1.856)** | **<0.001** |
| **90-94** | **3.635 (3.557 to 3.715)** | **<0.001** | **4.195 (4.074 to 4.32)** | **<0.001** | **3.796 (3.67 to 3.927)** | **<0.001** | **1.639 (1.627 to 1.65)** | **<0.001** | **1.94 (1.915 to 1.965)** | **<0.001** | **1.754 (1.728 to 1.782)** | **<0.001** |
| **95 plus** | **3.467 (3.342 to 3.596)** | **<0.001** | **4.713 (4.504 to 4.931)** | **<0.001** | **2.895 (2.716 to 3.086)** | **<0.001** | **1.506 (1.487 to 1.525)** | **<0.001** | **2.107 (2.063 to 2.151)** | **<0.001** | **1.294 (1.256 to 1.334)** | **<0.001** |
| **Period** | | | | | | | | | | | | |
| **1992** | **1 (0.991 to 1.008)** | **0.95** | **1.051 (1.037 to 1.065)** | **<0.001** | **0.967 (0.955 to 0.978)** | **<0.001** | **1.111 (1.109 to 1.114)** | **<0.001** | **1.163 (1.158 to 1.168)** | **<0.001** | **1.073 (1.069 to 1.078)** | **<0.001** |
| **1997** | **0.993 (0.986 to 1.001)** | **0.073** | **1.023 (1.01 to 1.036)** | **<0.001** | **0.973 (0.964 to 0.983)** | **<0.001** | **1.062 (1.061 to 1.064)** | **<0.001** | **1.094 (1.09 to 1.098)** | **<0.001** | **1.04 (1.036 to 1.043)** | **<0.001** |
| **2002** | **1.023 (1.016 to 1.03)** | **<0.001** | **1.026 (1.014 to 1.038)** | **<0.001** | **1.02 (1.012 to 1.029)** | **<0.001** | **1.049 (1.048 to 1.051)** | **<0.001** | **1.054 (1.05 to 1.057)** | **<0.001** | **1.045 (1.042 to 1.047)** | **<0.001** |
| **2007** | **0.986 (0.979 to 0.993)** | **<0.001** | **0.964 (0.952 to 0.975)** | **<0.001** | **0.999 (0.991 to 1.008)** | **0.898** | **0.967 (0.965 to 0.968)** | **<0.001** | **0.945 (0.942 to 0.949)** | **<0.001** | **0.98 (0.977 to 0.982)** | **<0.001** |
| **2012** | **0.979 (0.972 to 0.987)** | **<0.001** | **0.944 (0.932 to 0.956)** | **<0.001** | **1.002 (0.993 to 1.012)** | **0.617** | **0.916 (0.915 to 0.918)** | **<0.001** | **0.885 (0.882 to 0.888)** | **<0.001** | **0.939 (0.936 to 0.942)** | **<0.001** |
| **2017** | **1.019 (1.011 to 1.028)** | **<0.001** | **0.997 (0.983 to 1.01)** | **0.604** | **1.04 (1.027 to 1.052)** | **<0.001** | **0.911 (0.909 to 0.913)** | **<0.001** | **0.891 (0.888 to 0.895)** | **<0.001** | **0.933 (0.929 to 0.937)** | **<0.001** |
| **Birth cohort** | | | | | | | | | | | | |
| **1897-1901** | **2.178 (1.929 to 2.46)** | **<0.001** | **2.019 (1.742 to 2.34)** | **0** | **2.454 (1.978 to 3.046)** | **<0.001** | **1.458 (1.398 to 1.521)** | **<0.001** | **1.363 (1.27 to 1.462)** | **<0.001** | **1.66 (1.497 to 1.84)** | **<0.001** |
| **1902-1906** | **2.226 (2.108 to 2.35)** | **<0.001** | **2.175 (2.028 to 2.332)** | **0** | **2.311 (2.117 to 2.523)** | **<0.001** | **1.535 (1.507 to 1.564)** | **<0.001** | **1.514 (1.466 to 1.563)** | **<0.001** | **1.607 (1.543 to 1.674)** | **<0.001** |
| **1907-1911** | **2.177 (2.103 to 2.253)** | **<0.001** | **2.201 (2.103 to 2.303)** | **0** | **2.212 (2.095 to 2.336)** | **<0.001** | **1.567 (1.549 to 1.584)** | **<0.001** | **1.599 (1.567 to 1.631)** | **<0.001** | **1.601 (1.562 to 1.641)** | **<0.001** |
| **1912-1916** | **2.146 (2.09 to 2.205)** | **<0.001** | **2.171 (2.096 to 2.25)** | **0** | **2.209 (2.117 to 2.305)** | **<0.001** | **1.613 (1.599 to 1.626)** | **<0.001** | **1.641 (1.616 to 1.666)** | **<0.001** | **1.671 (1.639 to 1.703)** | **<0.001** |
| **1917-1921** | **2.058 (2.011 to 2.106)** | **<0.001** | **2.077 (2.014 to 2.142)** | **0** | **2.115 (2.039 to 2.194)** | **<0.001** | **1.61 (1.599 to 1.622)** | **<0.001** | **1.635 (1.614 to 1.656)** | **<0.001** | **1.663 (1.637 to 1.691)** | **<0.001** |
| **1922-1926** | **1.913 (1.875 to 1.952)** | **<0.001** | **1.922 (1.87 to 1.975)** | **0** | **1.97 (1.907 to 2.034)** | **<0.001** | **1.567 (1.557 to 1.577)** | **<0.001** | **1.578 (1.561 to 1.596)** | **<0.001** | **1.622 (1.599 to 1.645)** | **<0.001** |
| **1927-1931** | **1.812 (1.779 to 1.846)** | **<0.001** | **1.792 (1.747 to 1.838)** | **0** | **1.854 (1.801 to 1.909)** | **<0.001** | **1.55 (1.541 to 1.558)** | **<0.001** | **1.534 (1.519 to 1.55)** | **<0.001** | **1.591 (1.571 to 1.612)** | **<0.001** |
| **1932-1936** | **1.664 (1.636 to 1.693)** | **<0.001** | **1.619 (1.58 to 1.659)** | **0** | **1.693 (1.648 to 1.739)** | **<0.001** | **1.481 (1.474 to 1.489)** | **<0.001** | **1.444 (1.43 to 1.457)** | **<0.001** | **1.511 (1.494 to 1.528)** | **<0.001** |
| **1937-1941** | **1.462 (1.439 to 1.487)** | **<0.001** | **1.408 (1.374 to 1.443)** | **0** | **1.488 (1.451 to 1.525)** | **<0.001** | **1.361 (1.355 to 1.367)** | **<0.001** | **1.312 (1.301 to 1.323)** | **<0.001** | **1.387 (1.373 to 1.401)** | **<0.001** |
| **1942-1946** | **1.251 (1.231 to 1.272)** | **<0.001** | **1.207 (1.177 to 1.238)** | **0** | **1.259 (1.23 to 1.289)** | **<0.001** | **1.218 (1.213 to 1.223)** | **<0.001** | **1.175 (1.166 to 1.184)** | **<0.001** | **1.228 (1.217 to 1.238)** | **<0.001** |
| **1947-1951** | **1.097 (1.08 to 1.116)** | **<0.001** | **1.056 (1.028 to 1.084)** | **0** | **1.1 (1.075 to 1.125)** | **<0.001** | **1.119 (1.115 to 1.123)** | **<0.001** | **1.077 (1.069 to 1.084)** | **<0.001** | **1.121 (1.113 to 1.13)** | **<0.001** |
| **1952-1956** | **0.966 (0.95 to 0.982)** | **<0.001** | **0.933 (0.908 to 0.959)** | **0** | **0.966 (0.945 to 0.988)** | **0.002** | **1.029 (1.026 to 1.032)** | **<0.001** | **0.993 (0.986 to 0.999)** | **0.032** | **1.029 (1.022 to 1.035)** | **<0.001** |
| **1957-1961** | **0.795 (0.781 to 0.81)** | **<0.001** | **0.775 (0.752 to 0.798)** | **0** | **0.79 (0.772 to 0.809)** | **<0.001** | **0.883 (0.881 to 0.886)** | **<0.001** | **0.859 (0.853 to 0.865)** | **<0.001** | **0.875 (0.871 to 0.88)** | **<0.001** |
| **1962-1966** | **0.667 (0.654 to 0.68)** | **<0.001** | **0.668 (0.647 to 0.69)** | **0** | **0.654 (0.638 to 0.671)** | **<0.001** | **0.778 (0.776 to 0.78)** | **<0.001** | **0.776 (0.771 to 0.781)** | **<0.001** | **0.761 (0.757 to 0.765)** | **<0.001** |
| **1967-1971** | **0.599 (0.587 to 0.612)** | **<0.001** | **0.597 (0.576 to 0.618)** | **0** | **0.588 (0.572 to 0.604)** | **<0.001** | **0.73 (0.728 to 0.733)** | **<0.001** | **0.724 (0.719 to 0.729)** | **<0.001** | **0.713 (0.709 to 0.717)** | **<0.001** |
| **1972-1976** | **0.49 (0.477 to 0.502)** | **<0.001** | **0.508 (0.487 to 0.53)** | **0** | **0.469 (0.453 to 0.485)** | **<0.001** | **0.624 (0.621 to 0.626)** | **<0.001** | **0.644 (0.638 to 0.649)** | **<0.001** | **0.594 (0.59 to 0.598)** | **<0.001** |
| **1977-1981** | **0.402 (0.389 to 0.415)** | **<0.001** | **0.428 (0.407 to 0.451)** | **0** | **0.378 (0.362 to 0.394)** | **<0.001** | **0.535 (0.532 to 0.537)** | **<0.001** | **0.567 (0.562 to 0.573)** | **<0.001** | **0.499 (0.495 to 0.504)** | **<0.001** |
| **1982-1986** | **0.347 (0.332 to 0.362)** | **<0.001** | **0.374 (0.35 to 0.399)** | **0** | **0.323 (0.304 to 0.342)** | **<0.001** | **0.485 (0.482 to 0.488)** | **<0.001** | **0.519 (0.512 to 0.526)** | **<0.001** | **0.447 (0.442 to 0.453)** | **<0.001** |
| **1987-1991** | **0.321 (0.302 to 0.341)** | **<0.001** | **0.338 (0.309 to 0.371)** | **0** | **0.305 (0.28 to 0.331)** | **<0.001** | **0.469 (0.465 to 0.473)** | **<0.001** | **0.49 (0.482 to 0.499)** | **<0.001** | **0.441 (0.434 to 0.449)** | **<0.001** |
| **1992-1996** | **0.275 (0.242 to 0.313)** | **<0.001** | **0.289 (0.242 to 0.346)** | **0** | **0.262 (0.219 to 0.314)** | **<0.001** | **0.42 (0.413 to 0.427)** | **<0.001** | **0.438 (0.424 to 0.452)** | **<0.001** | **0.396 (0.383 to 0.409)** | **<0.001** |

**Abbreviations: RRs, relative risk; CI, conﬁdence interval.**

**Supplemental Table11 The predicted ASR of mortality of Gastric Cancer Attributable to High-Sodium Diet from 2022 to 2036.**

| **Location** | **Sex** | **2021** | **2022** | **2023** | **2024** | **2025** | **2026** | **2027** | **2028** | **2029** | **2030** | **2031** | **2032** | **2033** | **2034** | **2035** | **2036** |
| --- | --- | --- | --- | --- | --- | --- | --- | --- | --- | --- | --- | --- | --- | --- | --- | --- | --- |
| **Global** | **Female** | **0.996±0.006** | **0.981±0.013** | **0.968±0.021** | **0.956±0.031** | **0.943±0.042** | **0.930±0.054** | **0.917±0.067** | **0.905±0.080** | **0.892±0.094** | **0.880±0.108** | **0.868±0.122** | **0.856±0.137** | **0.844±0.152** | **0.833±0.167** | **0.821±0.183** | **0.810±0.199** |
| **Global** | **Male** | **2.349±0.010** | **2.309±0.030** | **2.272±0.050** | **2.235±0.073** | **2.197±0.099** | **2.159±0.128** | **2.122±0.158** | **2.086±0.189** | **2.049±0.221** | **2.012±0.254** | **1.977±0.288** | **1.942±0.322** | **1.908±0.357** | **1.874±0.391** | **1.841±0.426** | **1.808±0.461** |
| **High-income_North_America** | **Female** | **0.282±0.008** | **0.278±0.010** | **0.274±0.012** | **0.271±0.015** | **0.268±0.018** | **0.265±0.021** | **0.263±0.025** | **0.260±0.028** | **0.258±0.032** | **0.255±0.036** | **0.253±0.040** | **0.252±0.044** | **0.250±0.048** | **0.248±0.053** | **0.246±0.057** | **0.245±0.062** |
| **High-income_North_America** | **Male** | **0.568±0.013** | **0.558±0.017** | **0.548±0.021** | **0.539±0.026** | **0.530±0.031** | **0.522±0.037** | **0.514±0.043** | **0.506±0.049** | **0.498±0.056** | **0.491±0.062** | **0.484±0.069** | **0.478±0.076** | **0.472±0.083** | **0.466±0.090** | **0.460±0.098** | **0.454±0.105** |
| **Caribbean** | **Female** | **0.741±0.038** | **0.733±0.046** | **0.726±0.054** | **0.718±0.063** | **0.711±0.073** | **0.705±0.083** | **0.698±0.094** | **0.692±0.106** | **0.685±0.118** | **0.680±0.130** | **0.674±0.143** | **0.669±0.156** | **0.663±0.170** | **0.658±0.183** | **0.654±0.198** | **0.649±0.212** |
| **Caribbean** | **Male** | **1.425±0.060** | **1.415±0.074** | **1.406±0.089** | **1.398±0.106** | **1.389±0.124** | **1.381±0.144** | **1.373±0.165** | **1.366±0.187** | **1.358±0.211** | **1.351±0.235** | **1.344±0.260** | **1.338±0.285** | **1.331±0.312** | **1.325±0.339** | **1.319±0.367** | **1.313±0.396** |
| **Andean_Latin_America** | **Female** | **2.697±0.085** | **2.672±0.113** | **2.647±0.144** | **2.621±0.179** | **2.596±0.219** | **2.571±0.260** | **2.546±0.305** | **2.521±0.351** | **2.497±0.399** | **2.473±0.448** | **2.449±0.499** | **2.425±0.551** | **2.402±0.604** | **2.379±0.658** | **2.356±0.713** | **2.334±0.769** |
| **Andean_Latin_America** | **Male** | **3.622±0.105** | **3.604±0.140** | **3.586±0.179** | **3.568±0.224** | **3.550±0.273** | **3.532±0.327** | **3.514±0.384** | **3.496±0.444** | **3.478±0.507** | **3.461±0.573** | **3.443±0.640** | **3.426±0.710** | **3.409±0.782** | **3.392±0.856** | **3.375±0.931** | **3.359±1.009** |
| **Central_Latin_America** | **Female** | **1.335±0.029** | **1.315±0.039** | **1.295±0.050** | **1.277±0.063** | **1.260±0.076** | **1.243±0.091** | **1.227±0.107** | **1.212±0.123** | **1.198±0.140** | **1.185±0.157** | **1.173±0.175** | **1.161±0.194** | **1.150±0.213** | **1.139±0.232** | **1.129±0.252** | **1.120±0.272** |
| **Central_Latin_America** | **Male** | **2.114±0.042** | **2.100±0.060** | **2.090±0.079** | **2.080±0.102** | **2.071±0.127** | **2.063±0.155** | **2.055±0.184** | **2.048±0.215** | **2.042±0.248** | **2.037±0.282** | **2.032±0.318** | **2.028±0.356** | **2.025±0.395** | **2.022±0.435** | **2.020±0.477** | **2.018±0.520** |
| **Southern_Latin_America** | **Female** | **0.958±0.036** | **0.936±0.044** | **0.913±0.053** | **0.892±0.062** | **0.870±0.073** | **0.850±0.084** | **0.830±0.095** | **0.810±0.106** | **0.791±0.117** | **0.773±0.129** | **0.755±0.140** | **0.737±0.152** | **0.720±0.163** | **0.704±0.174** | **0.688±0.186** | **0.672±0.196** |
| **Southern_Latin_America** | **Male** | **2.432±0.070** | **2.367±0.090** | **2.301±0.110** | **2.237±0.133** | **2.174±0.158** | **2.113±0.183** | **2.054±0.209** | **1.996±0.235** | **1.940±0.261** | **1.885±0.287** | **1.832±0.313** | **1.780±0.339** | **1.729±0.363** | **1.680±0.388** | **1.632±0.411** | **1.585±0.434** |
| **Tropical_Latin_America** | **Female** | **0.894±0.022** | **0.879±0.029** | **0.865±0.037** | **0.852±0.045** | **0.840±0.055** | **0.827±0.064** | **0.816±0.075** | **0.804±0.086** | **0.793±0.097** | **0.783±0.108** | **0.773±0.120** | **0.763±0.132** | **0.754±0.145** | **0.745±0.157** | **0.736±0.170** | **0.728±0.183** |
| **Tropical_Latin_America** | **Male** | **2.109±0.041** | **2.071±0.056** | **2.032±0.072** | **1.993±0.089** | **1.955±0.109** | **1.918±0.129** | **1.881±0.150** | **1.845±0.172** | **1.811±0.195** | **1.777±0.218** | **1.743±0.241** | **1.711±0.264** | **1.679±0.288** | **1.648±0.311** | **1.618±0.335** | **1.588±0.358** |
| **Western_Europe** | **Female** | **0.483±0.009** | **0.473±0.012** | **0.463±0.016** | **0.453±0.020** | **0.443±0.024** | **0.434±0.029** | **0.425±0.033** | **0.416±0.038** | **0.407±0.043** | **0.398±0.048** | **0.390±0.053** | **0.382±0.058** | **0.374±0.063** | **0.366±0.068** | **0.359±0.073** | **0.351±0.078** |
| **Western_Europe** | **Male** | **1.075±0.016** | **1.050±0.022** | **1.024±0.029** | **1.000±0.037** | **0.976±0.045** | **0.952±0.054** | **0.930±0.063** | **0.908±0.073** | **0.886±0.082** | **0.865±0.092** | **0.845±0.102** | **0.825±0.111** | **0.806±0.121** | **0.787±0.130** | **0.768±0.140** | **0.750±0.149** |
| **Central_Europe** | **Female** | **0.795±0.022** | **0.776±0.028** | **0.759±0.034** | **0.742±0.041** | **0.725±0.049** | **0.709±0.057** | **0.693±0.066** | **0.677±0.075** | **0.662±0.083** | **0.647±0.092** | **0.632±0.101** | **0.617±0.110** | **0.603±0.118** | **0.589±0.127** | **0.575±0.135** | **0.561±0.143** |
| **Central_Europe** | **Male** | **1.938±0.042** | **1.888±0.056** | **1.839±0.070** | **1.791±0.086** | **1.743±0.103** | **1.697±0.121** | **1.651±0.140** | **1.606±0.159** | **1.562±0.177** | **1.518±0.196** | **1.475±0.214** | **1.433±0.232** | **1.392±0.250** | **1.351±0.267** | **1.311±0.284** | **1.272±0.300** |
| **Eastern_Europe** | **Female** | **1.023±0.021** | **1.002±0.029** | **0.983±0.038** | **0.964±0.048** | **0.946±0.059** | **0.928±0.071** | **0.911±0.083** | **0.894±0.095** | **0.877±0.108** | **0.861±0.121** | **0.845±0.134** | **0.829±0.147** | **0.814±0.160** | **0.799±0.173** | **0.785±0.187** | **0.770±0.200** |
| **Eastern_Europe** | **Male** | **2.732±0.049** | **2.660±0.081** | **2.590±0.117** | **2.522±0.159** | **2.454±0.205** | **2.386±0.253** | **2.320±0.302** | **2.257±0.352** | **2.195±0.403** | **2.134±0.454** | **2.074±0.504** | **2.016±0.554** | **1.960±0.604** | **1.905±0.652** | **1.852±0.700** | **1.799±0.746** |
| **North_Africa_and_Middle_East** | **Female** | **0.485±0.013** | **0.477±0.017** | **0.469±0.021** | **0.461±0.026** | **0.454±0.031** | **0.446±0.036** | **0.439±0.042** | **0.431±0.047** | **0.424±0.053** | **0.417±0.059** | **0.410±0.066** | **0.404±0.072** | **0.397±0.078** | **0.391±0.084** | **0.385±0.091** | **0.379±0.097** |
| **North_Africa_and_Middle_East** | **Male** | **1.156±0.022** | **1.134±0.031** | **1.111±0.040** | **1.089±0.050** | **1.068±0.061** | **1.046±0.073** | **1.025±0.085** | **1.005±0.098** | **0.985±0.111** | **0.965±0.124** | **0.945±0.137** | **0.926±0.150** | **0.908±0.164** | **0.890±0.177** | **0.872±0.190** | **0.854±0.203** |
| **Central_Asia** | **Female** | **0.968±0.038** | **0.936±0.046** | **0.906±0.054** | **0.876±0.063** | **0.848±0.072** | **0.820±0.082** | **0.793±0.091** | **0.767±0.101** | **0.742±0.111** | **0.717±0.120** | **0.694±0.130** | **0.671±0.139** | **0.648±0.148** | **0.627±0.156** | **0.606±0.164** | **0.586±0.172** |
| **Central_Asia** | **Male** | **2.519±0.076** | **2.434±0.097** | **2.351±0.118** | **2.270±0.142** | **2.191±0.167** | **2.115±0.192** | **2.040±0.218** | **1.968±0.244** | **1.898±0.269** | **1.831±0.294** | **1.765±0.318** | **1.701±0.341** | **1.640±0.363** | **1.580±0.385** | **1.523±0.405** | **1.468±0.424** |
| **South_Asia** | **Female** | **0.568±0.009** | **0.557±0.014** | **0.546±0.019** | **0.535±0.025** | **0.523±0.032** | **0.512±0.039** | **0.501±0.046** | **0.491±0.053** | **0.480±0.061** | **0.469±0.068** | **0.458±0.076** | **0.448±0.083** | **0.438±0.091** | **0.427±0.098** | **0.417±0.105** | **0.407±0.112** |
| **South_Asia** | **Male** | **1.095±0.014** | **1.080±0.023** | **1.064±0.032** | **1.048±0.043** | **1.032±0.054** | **1.016±0.067** | **1.000±0.081** | **0.984±0.095** | **0.968±0.109** | **0.952±0.123** | **0.936±0.138** | **0.920±0.153** | **0.904±0.169** | **0.888±0.184** | **0.872±0.199** | **0.856±0.214** |
| **Southeast_Asia** | **Female** | **0.726±0.014** | **0.716±0.020** | **0.706±0.026** | **0.697±0.033** | **0.687±0.040** | **0.677±0.048** | **0.667±0.057** | **0.657±0.066** | **0.648±0.075** | **0.638±0.084** | **0.628±0.094** | **0.619±0.104** | **0.609±0.113** | **0.600±0.123** | **0.590±0.133** | **0.581±0.142** |
| **Southeast_Asia** | **Male** | **1.370±0.022** | **1.358±0.031** | **1.346±0.041** | **1.334±0.052** | **1.322±0.065** | **1.310±0.079** | **1.298±0.094** | **1.286±0.109** | **1.275±0.125** | **1.263±0.142** | **1.251±0.159** | **1.239±0.176** | **1.227±0.194** | **1.215±0.212** | **1.203±0.231** | **1.190±0.249** |
| **East_Asia** | **Female** | **1.802±0.016** | **1.777±0.041** | **1.754±0.071** | **1.732±0.107** | **1.710±0.147** | **1.687±0.191** | **1.665±0.238** | **1.643±0.287** | **1.621±0.338** | **1.599±0.390** | **1.578±0.444** | **1.557±0.500** | **1.536±0.556** | **1.516±0.613** | **1.497±0.672** | **1.478±0.731** |
| **East_Asia** | **Male** | **4.885±0.030** | **4.797±0.104** | **4.708±0.174** | **4.620±0.258** | **4.533±0.352** | **4.447±0.453** | **4.363±0.559** | **4.280±0.671** | **4.198±0.785** | **4.118±0.902** | **4.041±1.021** | **3.966±1.142** | **3.894±1.264** | **3.823±1.388** | **3.755±1.512** | **3.690±1.637** |
| **High-income_Asia_Pacific** | **Female** | **1.154±0.019** | **1.119±0.027** | **1.086±0.035** | **1.054±0.044** | **1.023±0.054** | **0.993±0.064** | **0.963±0.075** | **0.934±0.085** | **0.905±0.095** | **0.877±0.106** | **0.850±0.116** | **0.824±0.126** | **0.798±0.136** | **0.772±0.145** | **0.748±0.154** | **0.724±0.163** |
| **High-income_Asia_Pacific** | **Male** | **2.998±0.039** | **2.902±0.060** | **2.817±0.082** | **2.734±0.107** | **2.651±0.133** | **2.569±0.160** | **2.488±0.188** | **2.408±0.216** | **2.330±0.243** | **2.253±0.270** | **2.178±0.296** | **2.104±0.321** | **2.031±0.346** | **1.960±0.369** | **1.891±0.391** | **1.823±0.413** |
| **Australasia** | **Female** | **0.278±0.022** | **0.274±0.026** | **0.270±0.029** | **0.266±0.033** | **0.263±0.038** | **0.259±0.042** | **0.255±0.047** | **0.252±0.051** | **0.248±0.056** | **0.244±0.061** | **0.241±0.066** | **0.237±0.071** | **0.234±0.076** | **0.231±0.081** | **0.227±0.087** | **0.224±0.092** |
| **Australasia** | **Male** | **0.646±0.039** | **0.636±0.046** | **0.626±0.053** | **0.617±0.061** | **0.608±0.070** | **0.599±0.080** | **0.590±0.090** | **0.581±0.100** | **0.573±0.110** | **0.565±0.121** | **0.557±0.132** | **0.549±0.143** | **0.541±0.154** | **0.534±0.165** | **0.527±0.177** | **0.520±0.188** |
| **Oceania** | **Female** | **1.357±0.147** | **1.351±0.166** | **1.346±0.186** | **1.341±0.208** | **1.336±0.233** | **1.332±0.259** | **1.327±0.287** | **1.323±0.316** | **1.320±0.347** | **1.317±0.379** | **1.314±0.412** | **1.312±0.447** | **1.310±0.483** | **1.309±0.520** | **1.309±0.559** | **1.310±0.599** |
| **Oceania** | **Male** | **2.273±0.188** | **2.254±0.215** | **2.235±0.244** | **2.216±0.276** | **2.197±0.311** | **2.179±0.348** | **2.161±0.388** | **2.143±0.429** | **2.126±0.471** | **2.109±0.515** | **2.092±0.561** | **2.076±0.607** | **2.060±0.655** | **2.044±0.703** | **2.029±0.753** | **2.015±0.803** |
| **Western_Sub-Saharan_Africa** | **Female** | **0.755±0.025** | **0.746±0.032** | **0.737±0.040** | **0.728±0.049** | **0.718±0.058** | **0.709±0.068** | **0.699±0.079** | **0.689±0.090** | **0.679±0.101** | **0.669±0.112** | **0.660±0.124** | **0.650±0.136** | **0.640±0.148** | **0.631±0.159** | **0.621±0.171** | **0.612±0.183** |
| **Western_Sub-Saharan_Africa** | **Male** | **1.054±0.032** | **1.042±0.041** | **1.029±0.051** | **1.016±0.062** | **1.004±0.074** | **0.991±0.088** | **0.978±0.102** | **0.966±0.116** | **0.954±0.131** | **0.942±0.146** | **0.930±0.162** | **0.918±0.178** | **0.906±0.194** | **0.895±0.210** | **0.884±0.227** | **0.872±0.243** |
| **Central_Sub-Saharan_Africa** | **Female** | **0.708±0.040** | **0.702±0.047** | **0.695±0.055** | **0.688±0.064** | **0.682±0.074** | **0.675±0.084** | **0.668±0.094** | **0.661±0.106** | **0.654±0.117** | **0.648±0.129** | **0.641±0.141** | **0.634±0.153** | **0.627±0.166** | **0.620±0.178** | **0.613±0.191** | **0.607±0.204** |
| **Central_Sub-Saharan_Africa** | **Male** | **1.226±0.062** | **1.212±0.073** | **1.198±0.086** | **1.184±0.100** | **1.170±0.115** | **1.156±0.131** | **1.142±0.149** | **1.128±0.166** | **1.114±0.185** | **1.100±0.204** | **1.087±0.223** | **1.073±0.243** | **1.060±0.263** | **1.047±0.283** | **1.033±0.303** | **1.020±0.324** |
| **Eastern_Sub-Saharan_Africa** | **Female** | **0.904±0.029** | **0.896±0.038** | **0.888±0.047** | **0.880±0.058** | **0.871±0.069** | **0.863±0.082** | **0.854±0.095** | **0.845±0.108** | **0.835±0.122** | **0.826±0.137** | **0.816±0.151** | **0.806±0.166** | **0.796±0.181** | **0.786±0.196** | **0.776±0.212** | **0.766±0.227** |
| **Eastern_Sub-Saharan_Africa** | **Male** | **1.040±0.034** | **1.023±0.043** | **1.007±0.053** | **0.990±0.065** | **0.974±0.077** | **0.958±0.090** | **0.941±0.104** | **0.925±0.118** | **0.910±0.132** | **0.894±0.147** | **0.879±0.162** | **0.864±0.176** | **0.849±0.192** | **0.834±0.207** | **0.820±0.222** | **0.806±0.237** |
| **Southern_Sub-Saharan_Africa** | **Female** | **0.654±0.037** | **0.641±0.045** | **0.628±0.054** | **0.616±0.064** | **0.603±0.075** | **0.591±0.086** | **0.579±0.098** | **0.567±0.110** | **0.555±0.122** | **0.544±0.134** | **0.532±0.146** | **0.521±0.158** | **0.509±0.170** | **0.498±0.182** | **0.486±0.194** | **0.475±0.205** |
| **Southern_Sub-Saharan_Africa** | **Male** | **1.012±0.054** | **0.987±0.065** | **0.963±0.078** | **0.938±0.091** | **0.915±0.106** | **0.891±0.122** | **0.868±0.137** | **0.845±0.153** | **0.823±0.169** | **0.801±0.185** | **0.779±0.200** | **0.757±0.216** | **0.736±0.230** | **0.715±0.245** | **0.695±0.259** | **0.675±0.273** |

**Abbreviations: ASR, age-standardized rate.**

**Supplemental Table12 The predicted ASR of DALYs of Gastric Cancer Attributable to High-Sodium Diet from 2022 to 2036.**

| **Location** | **Sex** | **2021** | **2022** | **2023** | **2024** | **2025** | **2026** | **2027** | **2028** | **2029** | **2030** | **2031** | **2032** | **2033** | **2034** | **2035** | **2036** |
| --- | --- | --- | --- | --- | --- | --- | --- | --- | --- | --- | --- | --- | --- | --- | --- | --- | --- |
| **Global** | **Female** | **22.892±0.030** | **22.433±0.297** | **22.096±0.463** | **21.762±0.670** | **21.425±0.903** | **21.090±1.156** | **20.763±1.427** | **20.443±1.711** | **20.125±2.005** | **19.805±2.306** | **19.489±2.614** | **19.183±2.929** | **18.882±3.248** | **18.583±3.570** | **18.283±3.893** | **17.988±4.218** |
| **Global** | **Male** | **54.265±0.049** | **53.190±0.776** | **52.234±1.190** | **51.266±1.704** | **50.267±2.282** | **49.297±2.907** | **48.367±3.575** | **47.471±4.272** | **46.564±4.986** | **45.626±5.708** | **44.716±6.443** | **43.858±7.194** | **43.043±7.955** | **42.225±8.715** | **41.378±9.464** | **40.550±10.211** |
| **High-income_North_America** | **Female** | **6.945±0.054** | **6.878±0.096** | **6.811±0.140** | **6.746±0.193** | **6.686±0.253** | **6.628±0.318** | **6.572±0.388** | **6.519±0.462** | **6.469±0.539** | **6.420±0.620** | **6.374±0.703** | **6.329±0.790** | **6.285±0.879** | **6.242±0.971** | **6.200±1.065** | **6.160±1.161** |
| **High-income_North_America** | **Male** | **13.541±0.082** | **13.317±0.166** | **13.120±0.248** | **12.930±0.346** | **12.750±0.456** | **12.583±0.574** | **12.425±0.701** | **12.274±0.835** | **12.129±0.973** | **11.992±1.117** | **11.866±1.266** | **11.749±1.420** | **11.637±1.579** | **11.529±1.741** | **11.427±1.907** | **11.335±2.078** |
| **Caribbean** | **Female** | **18.105±0.255** | **17.903±0.377** | **17.706±0.508** | **17.513±0.662** | **17.326±0.831** | **17.144±1.015** | **16.969±1.210** | **16.800±1.415** | **16.636±1.629** | **16.476±1.850** | **16.321±2.078** | **16.171±2.313** | **16.027±2.554** | **15.886±2.801** | **15.748±3.052** | **15.614±3.308** |
| **Caribbean** | **Male** | **34.713±0.393** | **34.433±0.642** | **34.146±0.905** | **33.872±1.218** | **33.604±1.568** | **33.341±1.948** | **33.087±2.355** | **32.845±2.784** | **32.615±3.233** | **32.391±3.702** | **32.172±4.187** | **31.960±4.689** | **31.757±5.206** | **31.562±5.738** | **31.372±6.283** | **31.186±6.840** |
| **Andean_Latin_America** | **Female** | **59.058±0.560** | **57.032±1.867** | **55.204±3.554** | **53.429±5.498** | **51.701±7.606** | **50.024±9.820** | **48.403±12.103** | **46.830±14.421** | **45.303±16.749** | **43.816±19.065** | **42.373±21.354** | **40.976±23.607** | **39.622±25.811** | **38.305±27.953** | **37.020±30.022** | **35.773±32.017** |
| **Andean_Latin_America** | **Male** | **81.616±0.677** | **79.860±1.972** | **78.248±3.609** | **76.656±5.533** | **75.080±7.660** | **73.538±9.942** | **72.037±12.346** | **70.568±14.843** | **69.116±17.407** | **67.678±20.018** | **66.270±22.665** | **64.897±25.339** | **63.550±28.024** | **62.217±30.704** | **60.894±33.367** | **59.597±36.013** |
| **Central_Latin_America** | **Female** | **31.693±0.184** | **31.218±0.413** | **30.791±0.653** | **30.386±0.941** | **29.991±1.262** | **29.613±1.611** | **29.252±1.985** | **28.914±2.380** | **28.592±2.792** | **28.277±3.218** | **27.974±3.660** | **27.684±4.115** | **27.412±4.584** | **27.150±5.064** | **26.892±5.553** | **26.643±6.051** |
| **Central_Latin_America** | **Male** | **51.058±0.260** | **50.941±0.703** | **50.931±1.209** | **50.942±1.829** | **50.975±2.540** | **51.010±3.330** | **51.044±4.193** | **51.104±5.125** | **51.182±6.120** | **51.277±7.178** | **51.374±8.293** | **51.470±9.465** | **51.585±10.695** | **51.713±11.981** | **51.853±13.321** | **51.992±14.713** |
| **Southern_Latin_America** | **Female** | **20.462±0.234** | **19.718±0.415** | **18.962±0.617** | **18.235±0.844** | **17.534±1.083** | **16.860±1.330** | **16.212±1.579** | **15.587±1.827** | **14.986±2.071** | **14.405±2.309** | **13.846±2.541** | **13.308±2.765** | **12.789±2.980** | **12.290±3.186** | **11.807±3.381** | **11.343±3.566** |
| **Southern_Latin_America** | **Male** | **53.697±0.445** | **51.565±0.926** | **49.472±1.444** | **47.469±2.029** | **45.541±2.648** | **43.691±3.284** | **41.922±3.927** | **40.229±4.567** | **38.610±5.196** | **37.054±5.811** | **35.564±6.407** | **34.140±6.985** | **32.778±7.541** | **31.478±8.073** | **30.232±8.581** | **29.041±9.064** |
| **Tropical_Latin_America** | **Female** | **21.531±0.145** | **21.362±0.296** | **21.220±0.460** | **21.085±0.658** | **20.958±0.881** | **20.837±1.125** | **20.718±1.388** | **20.604±1.668** | **20.496±1.962** | **20.393±2.271** | **20.294±2.593** | **20.196±2.927** | **20.099±3.272** | **20.006±3.628** | **19.916±3.994** | **19.828±4.370** |
| **Tropical_Latin_America** | **Male** | **49.693±0.251** | **49.011±0.605** | **48.237±0.969** | **47.483±1.406** | **46.732±1.893** | **45.992±2.418** | **45.282±2.976** | **44.611±3.562** | **43.965±4.170** | **43.329±4.795** | **42.706±5.437** | **42.112±6.095** | **41.555±6.770** | **41.028±7.457** | **40.514±8.155** | **40.017±8.863** |
| **Western_Europe** | **Female** | **10.201±0.060** | **10.020±0.145** | **9.724±0.200** | **9.432±0.265** | **9.149±0.337** | **8.878±0.413** | **8.615±0.492** | **8.354±0.572** | **8.096±0.652** | **7.845±0.731** | **7.605±0.810** | **7.371±0.888** | **7.140±0.964** | **6.909±1.037** | **6.686±1.108** | **6.470±1.176** |
| **Western_Europe** | **Male** | **23.173±0.097** | **22.636±0.250** | **21.988±0.362** | **21.352±0.494** | **20.733±0.640** | **20.133±0.794** | **19.550±0.955** | **18.982±1.118** | **18.424±1.282** | **17.879±1.446** | **17.351±1.609** | **16.839±1.770** | **16.339±1.929** | **15.846±2.083** | **15.365±2.233** | **14.899±2.379** |
| **Central_Europe** | **Female** | **18.224±0.142** | **17.829±0.250** | **17.477±0.361** | **17.129±0.490** | **16.790±0.631** | **16.462±0.782** | **16.138±0.941** | **15.813±1.104** | **15.490±1.270** | **15.173±1.439** | **14.867±1.610** | **14.564±1.782** | **14.260±1.954** | **13.960±2.124** | **13.664±2.294** | **13.376±2.463** |
| **Central_Europe** | **Male** | **44.835±0.261** | **43.722±0.547** | **42.636±0.828** | **41.558±1.155** | **40.498±1.511** | **39.462±1.890** | **38.452±2.285** | **37.447±2.688** | **36.455±3.094** | **35.484±3.501** | **34.540±3.909** | **33.621±4.316** | **32.714±4.718** | **31.822±5.111** | **30.953±5.498** | **30.112±5.877** |
| **Eastern_Europe** | **Female** | **25.846±0.156** | **25.321±0.709** | **25.052±1.058** | **24.787±1.496** | **24.507±1.993** | **24.222±2.534** | **23.952±3.113** | **23.705±3.726** | **23.461±4.366** | **23.208±5.026** | **22.951±5.706** | **22.706±6.406** | **22.478±7.127** | **22.250±7.861** | **22.012±8.604** | **21.772±9.355** |
| **Eastern_Europe** | **Male** | **67.567±0.299** | **65.262±1.714** | **63.651±2.996** | **62.060±4.524** | **60.422±6.200** | **58.778±7.978** | **57.224±9.841** | **55.798±11.774** | **54.397±13.742** | **52.971±15.713** | **51.554±17.680** | **50.207±19.656** | **48.953±21.645** | **47.714±23.611** | **46.463±25.528** | **45.224±27.400** |
| **North_Africa_and_Middle_East** | **Female** | **12.387±0.081** | **12.176±0.151** | **11.980±0.220** | **11.790±0.301** | **11.600±0.391** | **11.412±0.488** | **11.234±0.592** | **11.069±0.701** | **10.911±0.814** | **10.754±0.930** | **10.600±1.049** | **10.455±1.172** | **10.320±1.298** | **10.190±1.427** | **10.058±1.556** | **9.927±1.688** |
| **North_Africa_and_Middle_East** | **Male** | **27.229±0.130** | **26.627±0.311** | **26.098±0.492** | **25.582±0.708** | **25.069±0.947** | **24.566±1.203** | **24.078±1.474** | **23.609±1.755** | **23.153±2.045** | **22.701±2.339** | **22.262±2.639** | **21.838±2.943** | **21.430±3.250** | **21.033±3.559** | **20.644±3.869** | **20.267±4.179** |
| **Central_Asia** | **Female** | **24.313±0.250** | **23.537±0.426** | **22.820±0.575** | **22.132±0.750** | **21.473±0.941** | **20.831±1.141** | **20.201±1.347** | **19.584±1.555** | **18.990±1.763** | **18.422±1.971** | **17.871±2.178** | **17.330±2.381** | **16.802±2.580** | **16.292±2.775** | **15.803±2.965** | **15.329±3.151** |
| **Central_Asia** | **Male** | **63.455±0.493** | **61.531±1.107** | **59.621±1.717** | **57.807±2.429** | **56.047±3.199** | **54.294±4.006** | **52.587±4.841** | **50.945±5.687** | **49.382±6.537** | **47.871±7.384** | **46.383±8.221** | **44.938±9.050** | **43.544±9.864** | **42.214±10.663** | **40.930±11.443** | **39.681±12.201** |
| **South_Asia** | **Female** | **14.712±0.057** | **14.264±0.345** | **13.913±0.460** | **13.565±0.604** | **13.223±0.767** | **12.887±0.942** | **12.556±1.125** | **12.230±1.313** | **11.907±1.504** | **11.589±1.695** | **11.278±1.886** | **10.973±2.076** | **10.672±2.263** | **10.374±2.447** | **10.082±2.626** | **9.795±2.800** |
| **South_Asia** | **Male** | **27.087±0.081** | **26.566±0.603** | **26.158±0.792** | **25.749±1.035** | **25.341±1.315** | **24.935±1.624** | **24.531±1.953** | **24.125±2.297** | **23.717±2.653** | **23.308±3.016** | **22.900±3.386** | **22.493±3.761** | **22.084±4.137** | **21.671±4.512** | **21.258±4.886** | **20.845±5.257** |
| **Southeast_Asia** | **Female** | **17.773±0.085** | **17.544±0.198** | **17.301±0.310** | **17.056±0.444** | **16.810±0.594** | **16.565±0.755** | **16.320±0.927** | **16.075±1.107** | **15.827±1.292** | **15.579±1.482** | **15.331±1.676** | **15.084±1.873** | **14.836±2.072** | **14.586±2.271** | **14.334±2.470** | **14.084±2.669** |
| **Southeast_Asia** | **Male** | **33.838±0.129** | **33.593±0.354** | **33.280±0.549** | **32.971±0.788** | **32.656±1.058** | **32.335±1.352** | **32.015±1.669** | **31.697±2.002** | **31.382±2.351** | **31.061±2.713** | **30.735±3.085** | **30.409±3.468** | **30.082±3.859** | **29.757±4.257** | **29.428±4.660** | **29.096±5.068** |
| **East_Asia** | **Female** | **40.033±0.081** | **38.425±0.960** | **37.795±1.606** | **37.179±2.400** | **36.574±3.295** | **35.976±4.265** | **35.390±5.297** | **34.820±6.378** | **34.267±7.501** | **33.729±8.658** | **33.203±9.843** | **32.694±11.054** | **32.204±12.288** | **31.733±13.543** | **31.278±14.813** | **30.837±16.098** |
| **East_Asia** | **Male** | **111.777±0.143** | **108.533±2.804** | **106.263±4.354** | **104.046±6.277** | **101.874±8.437** | **99.774±10.771** | **97.753±13.240** | **95.815±15.813** | **93.941±18.465** | **92.117±21.178** | **90.369±23.945** | **88.712±26.766** | **87.154±29.635** | **85.667±32.540** | **84.227±35.468** | **82.855±38.425** |
| **High-income_Asia_Pacific** | **Female** | **24.371±0.146** | **23.831±0.466** | **23.234±0.629** | **22.648±0.829** | **22.071±1.054** | **21.508±1.293** | **20.954±1.543** | **20.411±1.797** | **19.879±2.053** | **19.357±2.310** | **18.848±2.567** | **18.349±2.822** | **17.860±3.074** | **17.381±3.321** | **16.912±3.563** | **16.455±3.800** |
| **High-income_Asia_Pacific** | **Male** | **60.422±0.229** | **59.017±0.791** | **57.894±1.322** | **56.792±1.947** | **55.694±2.632** | **54.615±3.365** | **53.460±4.136** | **52.324±4.931** | **51.216±5.742** | **50.131±6.564** | **49.085±7.399** | **47.997±8.233** | **46.927±9.067** | **45.879±9.895** | **44.853±10.717** | **43.874±11.537** |
| **Australasia** | **Female** | **6.549±0.149** | **6.445±0.200** | **6.335±0.254** | **6.226±0.316** | **6.120±0.383** | **6.015±0.455** | **5.912±0.530** | **5.811±0.607** | **5.711±0.686** | **5.613±0.767** | **5.516±0.849** | **5.421±0.931** | **5.327±1.014** | **5.234±1.097** | **5.142±1.180** | **5.051±1.263** |
| **Australasia** | **Male** | **14.641±0.245** | **14.456±0.349** | **14.263±0.458** | **14.076±0.584** | **13.892±0.723** | **13.713±0.873** | **13.540±1.032** | **13.370±1.197** | **13.203±1.369** | **13.038±1.545** | **12.877±1.726** | **12.717±1.910** | **12.560±2.098** | **12.404±2.288** | **12.248±2.480** | **12.094±2.673** |
| **Oceania** | **Female** | **33.117±0.840** | **32.975±1.100** | **32.840±1.381** | **32.710±1.708** | **32.585±2.073** | **32.464±2.470** | **32.348±2.896** | **32.236±3.346** | **32.129±3.819** | **32.027±4.314** | **31.930±4.828** | **31.837±5.362** | **31.748±5.914** | **31.664±6.483** | **31.586±7.069** | **31.511±7.672** |
| **Oceania** | **Male** | **58.551±1.122** | **58.369±1.550** | **58.184±2.003** | **58.014±2.538** | **57.858±3.139** | **57.716±3.796** | **57.587±4.502** | **57.471±5.254** | **57.369±6.046** | **57.280±6.878** | **57.203±7.748** | **57.138±8.654** | **57.085±9.594** | **57.043±10.569** | **57.013±11.577** | **56.992±12.618** |
| **Western_Sub-Saharan_Africa** | **Female** | **17.145±0.141** | **16.868±0.246** | **16.598±0.353** | **16.329±0.479** | **16.061±0.619** | **15.799±0.770** | **15.540±0.929** | **15.283±1.094** | **15.027±1.265** | **14.775±1.439** | **14.528±1.616** | **14.286±1.796** | **14.048±1.979** | **13.813±2.162** | **13.582±2.345** | **13.357±2.530** |
| **Western_Sub-Saharan_Africa** | **Male** | **24.281±0.181** | **23.866±0.331** | **23.475±0.483** | **23.090±0.664** | **22.710±0.864** | **22.338±1.080** | **21.974±1.308** | **21.617±1.546** | **21.268±1.791** | **20.926±2.043** | **20.592±2.300** | **20.268±2.561** | **19.951±2.827** | **19.643±3.095** | **19.343±3.366** | **19.052±3.638** |
| **Central_Sub-Saharan_Africa** | **Female** | **17.097±0.232** | **16.942±0.341** | **16.785±0.453** | **16.629±0.585** | **16.472±0.732** | **16.315±0.890** | **16.158±1.059** | **16.001±1.236** | **15.845±1.421** | **15.690±1.611** | **15.534±1.807** | **15.380±2.008** | **15.226±2.213** | **15.074±2.422** | **14.922±2.634** | **14.771±2.848** |
| **Central_Sub-Saharan_Africa** | **Male** | **29.290±0.352** | **28.912±0.558** | **28.564±0.777** | **28.225±1.037** | **27.894±1.327** | **27.571±1.641** | **27.256±1.974** | **26.951±2.325** | **26.655±2.689** | **26.367±3.067** | **26.088±3.456** | **25.818±3.856** | **25.556±4.266** | **25.303±4.684** | **25.059±5.110** | **24.822±5.544** |
| **Eastern_Sub-Saharan_Africa** | **Female** | **22.050±0.173** | **21.883±0.319** | **21.726±0.479** | **21.565±0.671** | **21.399±0.887** | **21.230±1.123** | **21.058±1.375** | **20.885±1.641** | **20.711±1.919** | **20.535±2.208** | **20.358±2.507** | **20.181±2.815** | **20.005±3.130** | **19.830±3.452** | **19.656±3.781** | **19.483±4.115** |
| **Eastern_Sub-Saharan_Africa** | **Male** | **24.890±0.196** | **24.486±0.362** | **24.159±0.531** | **23.841±0.732** | **23.530±0.958** | **23.227±1.202** | **22.932±1.461** | **22.646±1.734** | **22.371±2.018** | **22.106±2.312** | **21.853±2.615** | **21.611±2.927** | **21.379±3.247** | **21.158±3.574** | **20.948±3.907** | **20.749±4.248** |
| **Southern_Sub-Saharan_Africa** | **Female** | **16.304±0.270** | **16.352±0.710** | **16.274±1.105** | **16.192±1.591** | **16.106±2.144** | **16.014±2.752** | **15.917±3.406** | **15.816±4.100** | **15.710±4.828** | **15.597±5.586** | **15.478±6.370** | **15.354±7.177** | **15.224±8.003** | **15.090±8.844** | **14.948±9.697** | **14.800±10.559** |
| **Southern_Sub-Saharan_Africa** | **Male** | **26.891±0.369** | **26.794±0.754** | **26.668±1.210** | **26.535±1.756** | **26.398±2.371** | **26.251±3.043** | **26.096±3.765** | **25.935±4.531** | **25.769±5.334** | **25.599±6.171** | **25.421±7.038** | **25.237±7.933** | **25.050±8.851** | **24.861±9.791** | **24.669±10.750** | **24.473±11.725** |

**Abbreviations: ASR, age-standardized rate; DALYs, disability-adjusted life-years.**

**Supplemental Table13 The predicted ASR of mortality of Gastric Cancer Attributable to High-Sodium Diet from 2022 to 2036 globally by age.**

| **Year** | **Sex** | **25_29** | **30_34** | **35_39** | **40_44** | **45_49** | **50_54** | **55_59** | **60_64** | **65_69** | **70_74** | **75_79** | **80_84** | **85_89** | **90_94** | **95_** |
| --- | --- | --- | --- | --- | --- | --- | --- | --- | --- | --- | --- | --- | --- | --- | --- | --- |
| **2022** | **Male** | **0.0388±0.00260** | **0.1060±0.00346** | **0.218±0.00562** | **0.417±0.00965** | **0.755±0.0163** | **1.49±0.0309** | **2.55±0.0518** | **3.60±0.0726** | **5.7900±0.1150** | **8.4500±0.1680** | **10.6464±0.211** | **13.4935±0.269** | **18.2783±0.368** | **21.2202±0.445** | **17.7070±0.448** |
| **2022** | **Female** | **0.0395±0.00234** | **0.0814±0.00260** | **0.145±0.00373** | **0.235±0.00546** | **0.349±0.00765** | **0.597±0.01250** | **0.928±0.0190** | **1.36±0.0275** | **2.15±0.0424** | **3.26±0.0637** | **4.53±0.0885** | **6.1000±0.1190** | **8.2900±0.163** | **11.1969±0.227** | **13.6821±0.303** |
| **2023** | **Male** | **0.0383±0.00316** | **0.1050±0.00422** | **0.218±0.00699** | **0.413±0.01220** | **0.736±0.0207** | **1.44±0.0395** | **2.52±0.0680** | **3.56±0.0956** | **5.5900±0.1490** | **8.3500±0.2220** | **10.5425±0.280** | **13.2375±0.353** | **18.0547±0.484** | **21.1682±0.580** | **17.7518±0.548** |
| **2023** | **Female** | **0.0390±0.00278** | **0.0804±0.00316** | **0.144±0.00458** | **0.233±0.00686** | **0.343±0.00971** | **0.587±0.01610** | **0.918±0.0248** | **1.35±0.0362** | **2.09±0.0553** | **3.23±0.0850** | **4.48±0.1180** | **6.0100±0.1580** | **8.1700±0.216** | **11.1365±0.299** | **13.6820±0.387** |
| **2024** | **Male** | **0.0378±0.00380** | **0.1030±0.00528** | **0.218±0.00893** | **0.410±0.01580** | **0.719±0.0269** | **1.40±0.0514** | **2.46±0.0897** | **3.57±0.1290** | **5.3700±0.1940** | **8.2300±0.2980** | **10.4696±0.378** | **13.0175±0.471** | **17.7862±0.645** | **21.1207±0.776** | **17.7790±0.700** |
| **2024** | **Female** | **0.0385±0.00330** | **0.0793±0.00393** | **0.143±0.00578** | **0.231±0.00884** | **0.338±0.01260** | **0.577±0.02110** | **0.905±0.0327** | **1.35±0.0486** | **2.03±0.0728** | **3.19±0.1140** | **4.44±0.1580** | **5.9300±0.2120** | **8.0400±0.287** | **11.0635±0.399** | **13.6685±0.508** |
| **2025** | **Male** | **0.0373±0.00452** | **0.1020±0.00663** | **0.217±0.01130** | **0.409±0.02040** | **0.705±0.0344** | **1.35±0.0655** | **2.40±0.1150** | **3.59±0.1720** | **5.1500±0.2460** | **8.1200±0.3880** | **10.3886±0.496** | **12.8184±0.613** | **17.4544±0.836** | **21.0062±1.010** | **17.7497±0.892** |
| **2025** | **Female** | **0.0380±0.00388** | **0.0783±0.00487** | **0.141±0.00727** | **0.229±0.01130** | **0.333±0.01610** | **0.566±0.02700** | **0.891±0.0422** | **1.35±0.0638** | **1.98±0.0933** | **3.14±0.1470** | **4.41±0.2070** | **5.8600±0.2750** | **7.9000±0.371** | **10.9637±0.518** | **13.6298±0.655** |
| **2026** | **Male** | **0.0369±0.00531** | **0.1010±0.00823** | **0.215±0.01410** | **0.409±0.02580** | **0.693±0.0430** | **1.31±0.0810** | **2.33±0.1430** | **3.61±0.2210** | **4.9700±0.3040** | **7.9800±0.4880** | **10.3021±0.630** | **12.6499±0.774** | **17.0858±1.050** | **20.8191±1.280** | **17.6746±1.120** |
| **2026** | **Female** | **0.0376±0.00451** | **0.0773±0.00597** | **0.140±0.00898** | **0.227±0.01410** | **0.329±0.02010** | **0.556±0.03370** | **0.876±0.0528** | **1.35±0.0811** | **1.94±0.1170** | **3.08±0.1850** | **4.38±0.2620** | **5.8000±0.3470** | **7.7600±0.465** | **10.8408±0.651** | **13.5725±0.825** |
| **2027** | **Male** | **0.0365±0.00615** | **0.0994±0.01000** | **0.214±0.01720** | **0.410±0.03190** | **0.683±0.0526** | **1.28±0.0977** | **2.26±0.1720** | **3.60±0.2750** | **4.8600±0.3700** | **7.8000±0.5930** | **10.2053±0.776** | **12.5028±0.952** | **16.7296±1.270** | **20.5977±1.570** | **17.6083±1.370** |
| **2027** | **Female** | **0.0372±0.00519** | **0.0763±0.00719** | **0.138±0.01090** | **0.226±0.01720** | **0.325±0.02450** | **0.547±0.04090** | **0.862±0.0642** | **1.34±0.0999** | **1.92±0.1420** | **3.01±0.2240** | **4.35±0.3220** | **5.7400±0.4260** | **7.6400±0.567** | **10.7063±0.796** | **13.5118±1.010** |
| **2028** | **Male** | **0.0361±0.00704** | **0.0982±0.01200** | **0.211±0.02060** | **0.410±0.03860** | **0.677±0.0631** | **1.25±0.1160** | **2.19±0.2020** | **3.56±0.3290** | **4.8200±0.4450** | **7.5500±0.6970** | **10.0977±0.932** | **12.3956±1.140** | **16.4318±1.520** | **20.3700±1.890** | **17.5861±1.650** |
| **2028** | **Female** | **0.0367±0.00592** | **0.0754±0.00851** | **0.137±0.01290** | **0.224±0.02050** | **0.322±0.02920** | **0.538±0.04860** | **0.847±0.0763** | **1.33±0.1200** | **1.91±0.1710** | **2.94±0.2640** | **4.31±0.3870** | **5.6900±0.5100** | **7.5300±0.676** | **10.5620±0.949** | **13.4537±1.220** |
| **2029** | **Male** | **0.0358±0.00799** | **0.0971±0.01420** | **0.209±0.02420** | **0.410±0.04570** | **0.674±0.0744** | **1.22±0.1340** | **2.12±0.2330** | **3.49±0.3840** | **4.8300±0.5300** | **7.2600±0.7970** | **9.9700±1.090** | **12.3275±1.350** | **16.1818±1.780** | **20.0957±2.210** | **17.5716±1.950** |
| **2029** | **Female** | **0.0364±0.00668** | **0.0745±0.00993** | **0.135±0.01510** | **0.222±0.02400** | **0.320±0.03430** | **0.530±0.05660** | **0.834±0.0889** | **1.31±0.1400** | **1.91±0.2030** | **2.86±0.3040** | **4.26±0.4530** | **5.6400±0.6000** | **7.4500±0.792** | **10.4057±1.110** | **13.3830±1.430** |
| **2030** | **Male** | **0.0355±0.00900** | **0.0961±0.01650** | **0.207±0.02800** | **0.410±0.05320** | **0.673±0.0868** | **1.20±0.1540** | **2.06±0.2640** | **3.41±0.4370** | **4.8700±0.6240** | **6.9700±0.8940** | **9.8500±1.260** | **12.2525±1.570** | **15.9608±2.050** | **19.7537±2.540** | **17.5054±2.260** |
| **2030** | **Female** | **0.0360±0.00749** | **0.0737±0.01140** | **0.134±0.01750** | **0.220±0.02770** | **0.318±0.03970** | **0.523±0.06510** | **0.820±0.1020** | **1.29±0.1610** | **1.91±0.2370** | **2.79±0.3470** | **4.20±0.5220** | **5.6100±0.6960** | **7.3700±0.915** | **10.2403±1.270** | **13.2826±1.650** |
| **2031** | **Male** | **0.0353±0.01010** | **0.0951±0.01890** | **0.204±0.03220** | **0.408±0.06110** | **0.674±0.1000** | **1.18±0.1750** | **2.00±0.2960** | **3.31±0.4900** | **4.9000±0.7250** | **6.7400±0.9970** | **9.7000±1.430** | **12.1736±1.800** | **15.7810±2.330** | **19.3732±2.870** | **17.3825±2.580** |
| **2031** | **Female** | **0.0357±0.00834** | **0.0729±0.01300** | **0.132±0.02000** | **0.218±0.03160** | **0.316±0.04540** | **0.517±0.07410** | **0.807±0.1150** | **1.27±0.1820** | **1.91±0.2730** | **2.74±0.3920** | **4.13±0.5910** | **5.5800±0.7970** | **7.3000±1.040** | **10.0774±1.440** | **13.1565±1.880** |
| **2032** | **Male** | **0.0351±0.01120** | **0.0943±0.02150** | **0.202±0.03670** | **0.405±0.06920** | **0.676±0.1150** | **1.17±0.1970** | **1.95±0.3290** | **3.22±0.5430** | **4.9100±0.8280** | **6.6000±1.1100** | **9.5000±1.600** | **12.0850±2.040** | **15.6310±2.640** | **19.0102±3.210** | **17.2346±2.920** |
| **2032** | **Female** | **0.0354±0.00923** | **0.0722±0.01470** | **0.131±0.02260** | **0.216±0.03560** | **0.314±0.05140** | **0.512±0.08360** | **0.795±0.1290** | **1.26±0.2040** | **1.90±0.3100** | **2.71±0.4410** | **4.05±0.6590** | **5.5500±0.9020** | **7.2400±1.180** | **9.9300±1.620** | **13.0187±2.120** |
| **2033** | **Male** | **0.0350±0.01240** | **0.0936±0.02430** | **0.200±0.04150** | **0.402±0.07760** | **0.679±0.1300** | **1.16±0.2210** | **1.90±0.3630** | **3.13±0.5960** | **4.8700±0.9280** | **6.5600±1.2500** | **9.2200±1.760** | **11.9863±2.280** | **15.5340±2.960** | **18.7163±3.570** | **17.0848±3.270** |
| **2033** | **Female** | **0.0352±0.01020** | **0.0716±0.01640** | **0.129±0.02540** | **0.214±0.03980** | **0.313±0.05770** | **0.509±0.09360** | **0.783±0.1440** | **1.24±0.2270** | **1.89±0.3470** | **2.70±0.4960** | **3.96±0.7260** | **5.5100±1.0100** | **7.1900±1.320** | **9.8200±1.800** | **12.8713±2.370** |
| **2034** | **Male** | **0.0349±0.01370** | **0.0930±0.02720** | **0.198±0.04660** | **0.399±0.08630** | **0.681±0.1460** | **1.15±0.2470** | **1.87±0.4000** | **3.04±0.6500** | **4.7800±1.0200** | **6.6000±1.4100** | **8.8900±1.900** | **11.8694±2.530** | **15.4892±3.310** | **18.4800±3.950** | **16.8990±3.620** |
| **2034** | **Female** | **0.0350±0.01120** | **0.0711±0.01820** | **0.128±0.02830** | **0.212±0.04420** | **0.311±0.06410** | **0.506±0.10400** | **0.774±0.1590** | **1.22±0.2510** | **1.87±0.3840** | **2.71±0.5560** | **3.86±0.7930** | **5.4600±1.1200** | **7.1500±1.470** | **9.7300±2.000** | **12.7113±2.610** |
| **2035** | **Male** | **0.0349±0.01510** | **0.0926±0.03030** | **0.197±0.05200** | **0.395±0.09550** | **0.682±0.1630** | **1.16±0.2750** | **1.84±0.4380** | **2.96±0.7040** | **4.6800±1.1100** | **6.6700±1.5800** | **8.5600±2.030** | **11.7570±2.790** | **15.4391±3.670** | **18.2799±4.340** | **16.6590±3.970** |
| **2035** | **Female** | **0.0348±0.01220** | **0.0706±0.02020** | **0.127±0.03140** | **0.210±0.04880** | **0.309±0.07090** | **0.504±0.11500** | **0.765±0.1750** | **1.20±0.2750** | **1.85±0.4220** | **2.72±0.6210** | **3.78±0.8630** | **5.4100±1.2300** | **7.1300±1.630** | **9.6600±2.200** | **12.5418±2.870** |
| **2036** | **Male** | **0.0349±0.01660** | **0.0922±0.03350** | **0.196±0.05790** | **0.392±0.10500** | **0.681±0.1800** | **1.16±0.3060** | **1.82±0.4780** | **2.88±0.7580** | **4.5600±1.2000** | **6.7400±1.7700** | **8.3000±2.180** | **11.6192±3.050** | **15.3873±4.040** | **18.1301±4.770** | **16.3890±4.320** |
| **2036** | **Female** | **0.0347±0.01330** | **0.0702±0.02220** | **0.126±0.03470** | **0.209±0.05360** | **0.307±0.07780** | **0.503±0.12700** | **0.759±0.1920** | **1.19±0.3000** | **1.82±0.4600** | **2.73±0.6880** | **3.72±0.9390** | **5.3300±1.3400** | **7.1100±1.790** | **9.5900±2.420** | **12.3774±3.130** |

**Supplemental Table14 The predicted ASR of DALYs of Gastric Cancer Attributable to High-Sodium Diet from 2022 to 2036 globally by age.**

| **Year** | **Sex** | **25_29** | **30_34** | **35_39** | **40_44** | **45_49** | **50_54** | **55_59** | **60_64** | **65_69** | **70_74** | **75_79** | **80_84** | **85_89** | **90_94** | **95_** |
| --- | --- | --- | --- | --- | --- | --- | --- | --- | --- | --- | --- | --- | --- | --- | --- | --- |
| **2022** | **Male** | **2.31±0.0903** | **6.2300±0.1850** | **11.7392±0.3400** | **20.1272±0.5770** | **32.8249±0.935** | **57.9425±1.6400** | **86.0140±2.4400** | **104.5567±2.9700** | **141.6754±4.0200** | **170.5122±4.8300** | **172.1910±4.8800** | **171.3462±4.8500** | **183.8556±5.2200** | **185.4343±5.2800** | **142.1832±4.1200** |
| **2022** | **Female** | **2.40±0.0835** | **4.7600±0.1300** | **7.8300±0.2090** | **11.2820±0.2980** | **15.0041±0.3940** | **22.8755±0.5990** | **31.2111±0.817** | **39.0736±1.020** | **52.2202±1.3700** | **65.1764±1.7000** | **72.2964±1.8900** | **76.3350±2.0000** | **82.8571±2.1700** | **97.2903±2.5600** | **109.1033±2.8900** |
| **2023** | **Male** | **2.24±0.1140** | **6.0900±0.2110** | **11.7804±0.4010** | **19.9239±0.6710** | **31.9619±1.070** | **56.2270±1.8800** | **84.9429±2.8300** | **103.6201±3.4600** | **136.7147±4.5600** | **168.3353±5.6100** | **170.3421±5.6800** | **167.7649±5.6000** | **181.1771±6.0500** | **184.5454±6.1800** | **142.0659±4.8200** |
| **2023** | **Female** | **2.35±0.1040** | **4.6600±0.1490** | **7.7600±0.2440** | **11.1930±0.3480** | **14.7094±0.4560** | **22.4768±0.6950** | **30.8028±0.950** | **38.8308±1.200** | **50.6751±1.5600** | **64.4441±1.9900** | **71.4776±2.2000** | **75.0565±2.3200** | **81.5037±2.5200** | **96.5612±2.9900** | **108.7243±3.3900** |
| **2024** | **Male** | **2.18±0.1450** | **5.9300±0.2520** | **11.7730±0.4930** | **19.8338±0.8240** | **31.2498±1.290** | **54.4598±2.2500** | **83.1816±3.4300** | **103.7164±4.2800** | **131.0052±5.4000** | **165.8746±6.8400** | **168.9103±6.9700** | **164.6441±6.7900** | **178.1595±7.3500** | **183.7491±7.6000** | **141.9099±5.9200** |
| **2024** | **Female** | **2.30±0.1300** | **4.5600±0.1790** | **7.6600±0.2970** | **11.1302±0.4280** | **14.4363±0.5540** | **22.0677±0.8450** | **30.2808±1.160** | **38.8317±1.480** | **49.0832±1.8800** | **63.5703±2.4300** | **70.7901±2.7100** | **73.9604±2.8300** | **80.0444±3.0600** | **95.7534±3.6700** | **108.3075±4.1700** |
| **2025** | **Male** | **2.12±0.1800** | **5.7500±0.3050** | **11.7003±0.6110** | **19.8192±1.0300** | **30.5587±1.580** | **52.5794±2.7200** | **81.0762±4.1800** | **104.1026±5.3700** | **125.3612±6.4600** | **163.2589±8.4200** | **167.3584±8.6300** | **161.9055±8.3500** | **174.7156±9.0200** | **182.5635±9.4300** | **141.4806±7.3500** |
| **2025** | **Female** | **2.24±0.1610** | **4.4600±0.2190** | **7.5500±0.3650** | **11.0782±0.5330** | **14.1919±0.6820** | **21.6291±1.0400** | **29.7279±1.420** | **38.8732±1.860** | **47.6087±2.2800** | **62.5345±3.0000** | **70.1398±3.3600** | **72.9638±3.5000** | **78.5014±3.7600** | **94.7362±4.5500** | **107.7362±5.1800** |
| **2026** | **Male** | **2.06±0.2180** | **5.5900±0.3700** | **11.5665±0.7460** | **19.8798±1.2800** | **29.9210±1.920** | **50.7207±3.2500** | **78.8277±5.0400** | **104.4330±6.6800** | **120.9036±7.7300** | **160.1825±10.2460** | **165.7242±10.5989** | **159.6130±10.2094** | **170.9327±10.9371** | **180.7734±11.5764** | **140.7399±9.0400** |
| **2026** | **Female** | **2.19±0.1950** | **4.3600±0.2680** | **7.4300±0.4450** | **11.0274±0.6580** | **13.9890±0.8330** | **21.1799±1.2600** | **29.1937±1.740** | **38.8152±2.310** | **46.4530±2.7600** | **61.2618±3.6400** | **69.5071±4.1300** | **72.0443±4.2800** | **76.9584±4.5800** | **93.5183±5.5700** | **107.0323±6.3800** |
| **2027** | **Male** | **2.00±0.2580** | **5.4300±0.4460** | **11.3737±0.8950** | **19.9913±1.5700** | **29.4482±2.300** | **49.1218±3.8400** | **76.5778±5.9800** | **104.2020±8.1400** | **118.1687±9.2300** | **156.0770±12.1937** | **163.9470±12.8045** | **157.6449±12.3135** | **167.1851±13.0615** | **178.5445±13.9571** | **139.9776±10.9674** |
| **2027** | **Female** | **2.15±0.2310** | **4.2600±0.3230** | **7.3000±0.5340** | **10.9680±0.7990** | **13.8427±1.0100** | **20.7570±1.5100** | **28.7018±2.090** | **38.5624±2.800** | **45.7581±3.3200** | **59.7381±4.3400** | **68.8559±5.0000** | **71.1892±5.1700** | **75.5528±5.4900** | **92.1879±6.7000** | **106.2967±7.7400** |
| **2028** | **Male** | **1.95±0.3000** | **5.2700±0.5320** | **11.1288±1.0500** | **20.0849±1.8900** | **29.1848±2.740** | **47.8863±4.4900** | **74.3973±6.9700** | **103.0246±9.6500** | **117.2470±10.9834** | **150.7879±14.1295** | **162.0429±15.1803** | **156.1343±14.6280** | **163.8819±15.3562** | **176.1488±16.5127** | **139.4693±13.0948** |
| **2028** | **Female** | **2.10±0.2690** | **4.1700±0.3850** | **7.1600±0.6270** | **10.8792±0.9500** | **13.7475±1.2000** | **20.3698±1.7800** | **28.2302±2.460** | **38.0964±3.320** | **45.5198±3.9700** | **58.0292±5.0600** | **68.1511±5.9400** | **70.4541±6.1400** | **74.3625±6.4800** | **90.7738±7.9100** | **105.6068±9.2100** |
| **2029** | **Male** | **1.90±0.3430** | **5.1300±0.6250** | **10.8485±1.2000** | **20.1004±2.2300** | **29.0933±3.220** | **46.8848±5.1800** | **72.1597±7.9800** | **101.0291±11.1663** | **117.5199±12.9881** | **144.6922±15.9923** | **159.8971±17.6715** | **155.0380±17.1361** | **161.0579±17.8032** | **173.4567±19.1799** | **139.0613±15.3941** |
| **2029** | **Female** | **2.06±0.3080** | **4.0800±0.4520** | **7.0200±0.7240** | **10.7582±1.1100** | **13.6869±1.4100** | **20.0159±2.0600** | **27.7499±2.850** | **37.4960±3.850** | **45.5759±4.6800** | **56.2742±5.7800** | **67.3083±6.9200** | **69.8608±7.1800** | **73.3652±7.5400** | **89.2563±9.1800** | **104.8500±10.7893** |
| **2030** | **Male** | **1.85±0.3870** | **4.9900±0.7230** | **10.5533±1.3600** | **20.0086±2.5800** | **29.1191±3.750** | **45.9223±5.9100** | **69.7813±8.9700** | **98.6319±12.6787** | **118.1491±15.1871** | **138.6834±17.8254** | **157.6314±20.2619** | **153.8632±19.7785** | **158.6363±20.3936** | **170.3801±21.9088** | **138.3886±17.8104** |
| **2030** | **Female** | **2.02±0.3490** | **3.9900±0.5220** | **6.8700±0.8240** | **10.6183±1.2700** | **13.6421±1.6300** | **19.7047±2.3600** | **27.2366±3.260** | **36.8633±4.410** | **45.6888±5.4600** | **54.6606±6.5300** | **66.3049±7.9300** | **69.3166±8.2900** | **72.4786±8.6700** | **87.6590±10.4828** | **103.8823±12.4295** |
| **2031** | **Male** | **1.81±0.4330** | **4.8600±0.8260** | **10.2641±1.5200** | **19.8166±2.9300** | **29.2624±4.330** | **45.0475±6.6600** | **67.4395±9.9600** | **96.0747±14.1942** | **118.7443±17.5436** | **134.0004±19.7954** | **154.9483±22.8927** | **152.6437±22.5516** | **156.6805±23.1496** | **167.0007±24.6788** | **137.2861±20.3013** |
| **2031** | **Female** | **1.98±0.3910** | **3.9100±0.5950** | **6.7200±0.9280** | **10.4683±1.4400** | **13.6014±1.8700** | **19.4542±2.6700** | **26.7138±3.670** | **36.2592±4.980** | **45.6941±6.2800** | **53.4195±7.3400** | **65.0601±8.9400** | **68.8018±9.4500** | **71.6804±9.8500** | **86.0743±11.8276** | **102.7118±14.1198** |
| **2032** | **Male** | **1.77±0.4800** | **4.7300±0.9330** | **9.9900±1.7000** | **19.5271±3.2900** | **29.4882±4.960** | **44.4285±7.4700** | **65.4503±11.0051** | **93.5280±15.7254** | **118.7297±19.9630** | **131.2435±22.0642** | **151.2932±25.4389** | **151.3230±25.4417** | **155.0726±26.0736** | **163.6813±27.5248** | **135.8774±22.8613** |
| **2032** | **Female** | **1.94±0.4350** | **3.8300±0.6710** | **6.5800±1.0400** | **10.3063±1.6100** | **13.5527±2.1200** | **19.2857±3.0100** | **26.2280±4.100** | **35.7129±5.580** | **45.4788±7.1100** | **52.7157±8.2400** | **63.5569±9.9300** | **68.2808±10.6710** | **70.9580±11.0900** | **84.6555±13.2320** | **101.4343±15.8600** |
| **2033** | **Male** | **1.73±0.5280** | **4.6200±1.0400** | **9.7300±1.8800** | **19.1510±3.6400** | **29.6952±5.630** | **44.1336±8.3700** | **63.9526±12.1278** | **91.0764±17.2710** | **117.6614±22.3112** | **130.5230±24.7479** | **146.5064±27.7822** | **149.9136±28.4261** | **153.9442±29.1919** | **160.8208±30.4991** | **134.3662±25.4929** |
| **2033** | **Female** | **1.91±0.4800** | **3.7600±0.7500** | **6.4500±1.1500** | **10.1287±1.7900** | **13.4700±2.3700** | **19.1916±3.3800** | **25.7906±4.540** | **35.1968±6.200** | **45.0199±7.9300** | **52.5470±9.2600** | **61.8632±10.8991** | **67.7182±11.9305** | **70.3669±12.3975** | **83.4897±14.7107** | **100.0797±17.6387** |
| **2034** | **Male** | **1.69±0.5780** | **4.5100±1.1600** | **9.4800±2.0700** | **18.7163±3.9700** | **29.7942±6.330** | **44.1080±9.3600** | **62.7755±13.3221** | **88.5633±18.7939** | **115.6778±24.5455** | **131.1618±27.8303** | **140.9437±29.9072** | **148.3072±31.4690** | **153.2547±32.5207** | **158.4543±33.6267** | **132.6515±28.1606** |
| **2034** | **Female** | **1.88±0.5260** | **3.6900±0.8320** | **6.3300±1.2700** | **9.9400±1.9600** | **13.3498±2.6300** | **19.1494±3.7700** | **25.3986±5.010** | **34.6747±6.830** | **44.4086±8.7500** | **52.7285±10.3900** | **60.1253±11.8477** | **67.0291±13.2084** | **69.9291±13.7800** | **82.5527±16.2687** | **98.6249±19.4404** |
| **2035** | **Male** | **1.66±0.6310** | **4.4100±1.2800** | **9.2500±2.2700** | **18.2576±4.3100** | **29.7411±7.020** | **44.2704±10.4453** | **61.6586±14.5460** | **85.8836±20.2598** | **113.2487±26.7133** | **132.2328±31.1914** | **135.4681±31.9537** | **146.6145±34.5841** | **152.5187±35.9782** | **156.5082±36.9218** | **130.6629±30.8334** |
| **2035** | **Female** | **1.85±0.5740** | **3.6300±0.9170** | **6.2100±1.4000** | **9.7500±2.1400** | **13.2080±2.8900** | **19.1330±4.1900** | **25.0643±5.490** | **34.1157±7.470** | **43.7649±9.5800** | **52.9871±11.6003** | **58.5426±12.8159** | **66.1897±14.4909** | **69.5523±15.2269** | **81.7525±17.8990** | **97.0944±21.2617** |
| **2036** | **Male** | **1.63±0.6860** | **4.3200±1.4000** | **9.0400±2.4800** | **17.8109±4.6500** | **29.5451±7.710** | **44.6233±11.6383** | **60.6676±15.8220** | **83.2532±21.7105** | **110.6473±28.8537** | **133.3022±34.7622** | **131.2909±34.2356** | **144.5564±37.6976** | **151.7689±39.5785** | **155.0477±40.4360** | **128.4599±33.5097** |
| **2036** | **Female** | **1.82±0.6240** | **3.5700±1.0000** | **6.1000±1.5300** | **9.5700±2.3200** | **13.0558±3.1600** | **19.1261±4.6300** | **24.8107±6.000** | **33.5488±8.110** | **43.1607±10.4378** | **53.1324±12.8499** | **57.3637±13.8723** | **65.1176±15.7485** | **69.2171±16.7397** | **81.0645±19.6059** | **95.5896±23.1221** |

**Supplemental Table15: Average Daily Salt Intake Comparison (g/day per adult, approximate values based on recent estimates)**

| **Country/Region** | **Average Salt Intake (g/day)** | **Key Sources of Intake** |
| --- | --- | --- |
| China | 12.7 | High use of soy sauce, pickled vegetables, and table salt in home cooking |
| Mongolia | 11.06 | Elevated table salt in preserved meats and traditional diets |
| Global Average | 10.8 g | Primarily processed foods, varying by region |
